# Supplementary material for: Evaluating recovery following hip fracture: a qualitative interview study of what is important to patients
Source: BMJ Open. 2015 Jan 6;5(1):e005406. doi: 10.1136/bmjopen-2014-005406 (PMC4289715; doi:10.1136/bmjopen-2014-005406)
Supplement: Author's manuscript [file bmjopen-2014-005406.draft_revisions.pdf]

BMJ Open

Evaluating recovery following hip fracture: a qualitative study of what is important to patients

Journal:	*BMJ Open*
Manuscript ID:	bmjopen-2014-005406
Article Type:	Research
Date Submitted by the Author:	04-Apr-2014
Complete List of Authors:	Griffiths, Frances; University of Warwick, Warwick Medical School<br>Mason, Victoria; University of Warwick, Warwick Medical School<br>Boardman, Felicity; University of Warwick, Warwick Medical School<br>Dennick, Katherine; Kings College, London, Florence Nightingale School of Nursing and Midwifery<br>Haywood, Kirstie; University of Warwick, Warwick Medical School<br>Achten, Juul; University of Warwick, Warwick Medical School<br>Parsons, Nicholas; University of Warwick, Warwick Medical School<br>Griffin, Xavier; University of Warwick, Warwick Medical School<br>Costa, Matthew; University of Warwick, Warwick Medical School
**Primary Subject Heading**:	Health services research
Secondary Subject Heading:	Qualitative research, Surgery
Keywords:	Hip < ORTHOPAEDIC & TRAUMA SURGERY, Quality in health care < HEALTH SERVICES ADMINISTRATION & MANAGEMENT, QUALITATIVE RESEARCH

SCHOLARONE™
Manuscripts

For peer review only

Evaluating recovery following hip fracture: a qualitative study of what is important to patients

Frances Griffiths, Professor of Medicine in Society, Warwick Medical School, University of Warwick, Coventry, CV4 7AL, UK

Victoria Mason, Research Fellow, Warwick Medical School, University of Warwick, Coventry, CV4 7AL, UK

Felicity Boardman, Mildred Blaxter Post-Doctoral Fellow, Warwick Medical School, University of Warwick, Coventry, CV4 7AL, UK

Kathyrn Dennick, Research Associate, Florence Nightingale School of Nursing and Midwifery, King's College London, SE1 8WA

Kirstie Haywood, Senior Research Fellow, Royal College of Nursing Research Institute, University of Warwick, CV4 7AL, UK

Juul Achten, Research Manager Warwick Orthopaedics, Warwick Medical School, University of Warwick, Coventry, CV4 7AL, UK

Nick Parsons, Senior Research Fellow, Statistics and Epidemiology, Warwick Medical School, University of Warwick, Coventry, CV4 7AL, UK

Xavier Griffin, Specialist Trainee Trauma and Orthopaedic Surgery, Warwick Medical School, University of Warwick, Clinical Sciences Building, Clifford Bridge Road, Coventry, CV2 2DX, UK

Matt Costa, Professor Trauma and Orthopaedics, Warwick Clinical Trials Unit, Warwick Medical School, University of Warwick, Coventry, CV4 7AL, UK

Correspondence to: F.E.Griffiths@warwick.ac.uk

For peer review only

Copyright

The Corresponding Author has the right to grant on behalf of all authors and does grant on behalf of all authors, an exclusive licence (or non exclusive for government employees) on a worldwide basis to the BMJ Publishing Group Ltd to permit this article (if accepted) to be published in BMJ editions and any other BMJPGL products and sublicences such use and exploit all subsidiary rights, as set out in our licence.

For peer review only

Abstract

Objective

To explore what patients consider important when evaluating their recovery from hip fracture and to consider how these priorities could be used in the evaluation of the quality of hip fracture services.

Design

Semi-structured interviews exploring the experience of recovery from hip fracture at two time points - four weeks and four months post-operative hip fixation. Two approaches to analysis: thematic analysis of data specifically related to recovery from hip fracture; extraction of data into template to capture the participant's experience overall.

Participants

31 participants recruited, of whom 20 were female and 12 were cognitively impaired. Mean age 81.5 years. Interviews provided by 19 patients, 14 carers, and 8 patient/carer dyad; 10 participants were interviewed twice.

Setting

Single major trauma centre in the West Midlands of the UK.

Results

Stable mobility for valued activities was considered most important by participants who had some pre-fracture mobility and were able to articulate what they valued during recovery. Mobility was important for managing personal care, for day-to-day activities such as shopping and gardening, and maintenance of mental well-being. Some participants used assistive mobility devices or adapted to their limitations. Others maintained their previous limited function through increased care provision. Many participants were unable to articulate what they valued as hip fracture was perceived as part of their decline with age. The fracture and problems from other health conditions were an inseparable part of one health experience.

Conclusions

Patients consistently valued stable mobility and its role in other basic health domains. While no one patient-reported outcome measure (PROM) could consistently evaluate recovery for all patients with hip fracture, general health-related quality of life tools may provide useful information for the majority of patients. These may need to be supplemented by specific tools for selected groups, especially those patients with high-levels of pre-injury function.

Key words: Hip fractures, Outcome assessment (Health Care), Interview, Frail older adults

For peer review only

Article summary

Article focus

- The UK NHS has identified the need to evaluate service provision for patients with a hip fracture
- There is increasing expectation that patient-reported outcome measures (PROM) are used within health service evaluation
- We asked the question: what do patients who have recently experienced a hip fracture consider important when evaluating their recovery?

Key messages

- Patients active before their fracture value stable mobility to undertake valued activities but many patients consider fracture to be part of their decline with age.
- While no one PROM could evaluate all aspects of recovery for patients with hip fracture, general health-related quality of life tools may provide useful information for the majority of patients.

Strengths and limitations

- The study sample was representative of the age profile, gender balance and dementia levels of NHS patients experiencing hip fractures
- It is possible that those not agreeing to be interviewed were struggling most with recovery.
- The data is limited by the difficulty the more physically and cognitively impaired patients had in giving a detailed account of their health experience.

For peer review only

Introduction

Fragility fracture of the proximal femur (hip fracture) is one of the greatest challenges facing the healthcare community. In 1990, a global incidence of 1.31 million was reported and was associated with 740,000 deaths (1). Hip fractures constitute a heavy socioeconomic burden worldwide. The cost of this clinical problem is estimated at 1.75 million disability adjusted life years lost; 1.4% of the total healthcare burden in established market economies (1). Among those experiencing fragility hip fracture in England, Wales and Northern Ireland, 70% are aged 80 years or older, 73% are female and 34% are cognitively impaired pre-operation. The mortality rate within 30 days of operation was 8.2% in 2013 (2)

The NHS has identified the need to evaluate the quality of service provision for patients with a hip fracture; this evaluation is conducted through the National Hip Fracture Audit Database (NHFD)(2). Currently, aspects of care such as time to surgery, length of patient stay and patient mortality in hospital and 30 day and 120 day follow up are recorded in the NHFD. These data are now used to guide payments to healthcare providers; the payment being increased if the provider supplies 'best practice' care (3). However, while important, there is interest from policy makers in the potential to enhance these currently reported data fields by including and an assessment of outcome as reported by patients. It is increasingly expected that healthcare evaluations should include domains of health that are important to patients (4), captured by well-developed patient-reported outcome measures (PROMs) which aim to assess how patients function and feel in relation to a health condition or associated treatment (5). PROMS capture information that cannot be obtained by other means (5, 6) complementing more traditional performance or process-based measures.

Our aim was to establish whether or not one PROM could be used with all patients who experience a fragility hip fracture as part of the evaluation of the quality of health care for hip fracture delivered by the NHS. For this patient group we were unable to identify a PROM specific to the assessment of hip fracture, and robust evidence of the quality and acceptability of non-hip fracture specific PROMs following completion by patients sustaining a hip fracture is limited (7). Moreover, clarity with regards to the outcomes of healthcare that these patients considers relevant and important does not exist. Appropriate and relevant PROM-based assessment should be underpinned by an understanding of what is important to patients in terms of the outcomes of healthcare. We therefore designed an interview study to explore

For peer review only

with patients and, where appropriate, their carers, what they consider to be important outcomes and to explore variation across this patient group. Our research questions were:

1. What do patients who have recently experienced a hip fracture consider important when evaluating their recovery?
2. Is there variation within this population about what is considered important?

These research questions are framed by the desire of policy makers to evaluate the quality of care for hip fracture through assessment of recovery from the perspective of the patient.

Method

Study Design

We conducted semi-structured interviews with patients and, where appropriate, their carers at two time points, at approximately four weeks and then again at four months after they had sustained a fragility hip fracture.

Identification of patients with a hip fracture

We recruited participants from an existing cohort study, the Warwick Hip Trauma Evaluation (8), that commenced January 2012. This is a cohort of all patients admitted with a hip fracture to a single major trauma centre in the West Midlands of the United Kingdom. As part of their pre-operative assessment, patients were assessed for their capacity to consent using clinical assessment and the Abbreviated Mental Test Score (AMTS) (9). The AMTS is a 10-item measure used to rapidly assess the possibility of cognitive impairment in elderly people. A score below 8 suggests cognitive impairment (10). Scores less than 8 were taken to indicate that a patient was unlikely to be able to consent for themselves. Those deemed to have capacity for consenting to surgery, based on clinical assessment and AMTS, were considered able to consent for this study. Following the emergency surgery for their fracture, those with capacity gave written consent to be approached for interview. For those deemed not to have capacity due to cognitive impairment, verbal consent was obtained from their consultee (11). Ethical approval was granted by NHS REC London - Camberwell and St Giles (11/LO/0927) on the 18th August 2011.

Sampling

During the data collection period for this study, February to August 2012, we purposefully sampled cohort participants who had reached 4 weeks or 4 months following their hip

For peer review only - http://bmjopen.bmj.com/site/about/guidelines.xhtml

fracture and had consented to be approached for interview. The time points were chosen to be the same as those used for data collection for the NHFD (12). If a PROM were to be used with this patient population to assess quality of care, patients would be asked to complete the PROM at these time points. Our sampling strategy ensured a diverse mix of patients with respect to the following factors: age, gender, AMTS (9) and EQ-5D score (13).

Interview recruitment and consent process

We contacted eligible patients and carers just prior to 4 weeks and/or 4 months following hip fracture to arrange an interview. If patients declined to participate, the reasons offered were recorded. Patients with capacity to consent were contacted directly. For those patients deemed not to have capacity, we contacted their consultee. Patients able to consent for themselves signed their own consent forms. For those unable to consent the consultee signed an agreement form and we aimed to interview a carer as well as the patient (patient/carer dyad). Carers who were interviewed signed a consent form. The study flow diagram is at Figure 1.

Interview process

We interviewed participants at their current residence (own home, residential or nursing home) or in hospital. The interviewer was trained in interviewing but did not have clinical knowledge of hip fracture, its treatment or prognosis. Where possible, patients and carers were interviewed alone, however where the carer and patient requested a joint interview (whether or not the patient had cognitive impairment), they were interviewed together. The aim of the interviews was to understand each participant's lived experience of hip fracture (14) and the influence of their social context and pre-fracture health. The interviewer encouraged participants to talk about the experience in whatever order they chose and using terms meaningful to them. Later in the interview we prompted, where necessary, for clarification about what in the patient experience was related to the hip fracture. Towards the end of the interview we directly asked what was important to them in terms of recovery if this had not already been talked about by the participant. Consideration was given to the potential challenges associated with interviewing older adults, for example by giving potential participants sufficient time to decide whether or not to participate and minimising burden and fatigue through streamlining questions (15). The interview process, questions and prompts were refined by the study team during the initial stage of data collection. Interviews were

For peer review only

audio recorded and transcribed verbatim. For one interview, audio recording was not feasible due to the noisy environment so extensive field notes were taken and transcribed.

Analysis

Interview transcripts were checked, anonymised and uploaded into Nvivo software (16). Initial analysis involved data immersion, reading and re-reading each transcript, discussion of the interview transcripts by the research team. All team members read at least five transcripts. The key issues crystallised from this process (17). We found that the interviews at four weeks and four months covered very similar issues, although at four months reporting of fracture specific recovery was more advanced. For analysis, we therefore treated all the interviews related to one participant as one set of data. During data interpretation we took account of whether the interview data was from a patient or carer or patient/carer dyad. Two different approaches to analysis were then undertaken in response to our research questions.

To answer our first research question, we searched the transcripts for any mention of what was important during recovery from hip fracture and coded this text. As coding proceeded, we reviewed these codes and combined them into themes. After coding ten transcripts no additional themes were identified in the data. Double coding was undertaken for one in four transcripts and coding compared and discussed.

To answer our second research question, from close reading of the interview transcripts, we developed and refined a template for summarising the key issues of relevance to recovery from the hip fracture. The template included: current and recent past living arrangements and environment, day-to-day life now and in the recent past, the impact of the hip fracture and its management, what was changing in day-to-day life as they recovered, the extent to which the patient referred specifically to the fracture and their ability to engage in the interview. The data from each patient or patient/carer dyad was summarised with a second research team member reviewing each summary against the data. The summaries were then compared.

Results

Twenty one patients were interviewed on one occasion and 10 were interviewed twice giving a total of 31 patient participants and 41 interviews. Of the 31 patient participants, 20 (64.5%) were female, the mean age was 81.5 years (SD 9.2, range 61-96) and 12 scored less than eight on the AMTS. Of the 41 interviews, 24 were conducted three to nine weeks, and 17

For peer review only

were conducted 14 to 23 weeks after the hip fracture. Nineteen interviews were with the patient only, 14 with carer only, and eight with patient/carer dyads. Despite framing the interview for interviewees as exploring the experience of hip fracture, many interviewees talked about general health issues. Although we prompted to clarify what was related to their fracture, in many interviews it was difficult to disentangle the impact of the fracture from the impact of other health problems. Some interviews contained almost no data that was clearly related to the fracture. From the perspective of the patient, all their health problems were part of one experience. The absence of data clearly related to the fracture was more marked in the four month compared to two month interviews. We therefore decided not to attempt interviews at 12 months post fracture as originally planned (8). The following sections report our analysis. Illustrative quotations from data are labelled with the age and gender of the patient, time since hip fracture and whether the quotation was from the patient or carer.

What is important to patients when evaluating their recovery

From our systematic search of the interviews for data related to recovery from the hip fracture we identified the following themes: mobility, valued day-to-day activities, self-care, pain, mental wellbeing, fear of falling and leg shortening. When talking about mobility, day-to-day activities or self-care participants also talked about their level of independence.

Mobility

This was the most prominent theme, although when talking about mobility the interviewees often mentioned other themes. Mobile participants reported limited mobility in the weeks post operation and valued any improvement.

> I'm walking with a walking stick at the moment. I've been down the park and back…I can usually get around [the house] without the walking stick, and I can get up and down stairs no problem. I get upstairs with my good leg and downstairs with my bad leg. (Participant 6, male, age 78, 5 weeks post operation)

By four months, for many participants mobility had improved, and they were happy that they were returning to normal mobility.

> I can't rush round like I did, but eventually that will come…I mean it's pretty normal now, but I think it's going to be a while before I can actually walk as I did and I probably won't walk as I did… when I came home [from hospital] I was still hobbling... but now I'm more or less…walking normal, especially with the stick (Participant 10, female, age 83, 18 weeks post operation)

For peer review only

For those with limited mobility before hip fracture any unaided improvement was limited to the pre-fracture level but also valued.

> The operation was successful and got him back to normal right from the start, right from the very first day that he had it done. He was able to then walk pain free with a Zimmer frame to the toilet. The staff were all saying it was amazing how well he was walking and he would soon be back to normal, but what they didn't realise was that he was walking normally. (Carer of participant 1, male, age 84, 16 weeks post operation)

Other participants were using mobility aids that they had not been using regularly before the fracture. For some, the addition of mobility aids enabled greater security of mobility than prior to their fracture.

> Her mobility's getting better. I think she'll cope with the frame. She's had a couple of falls in the home, earlier when she was forgetting that she had to use the frame. She'd get out of bed and not use the frame and consequently fall. But she's got in the habit of using it now… she's not falling, which is a bonus. (Carer of participant 13, female, age 87, 14 weeks post operation)

Valued day-to-day activities

Those who were active prior to their fracture talked about the frustration of the restriction in their activities particularly in the weeks following the fracture.

> I'm back on what I call domestic duties – washing up! But the thing that is frustrating is that I can't get outside and do any gardening. (Participant 12, male, age 78, 6 weeks post operation)

> I just miss getting up and getting out. I never stayed in. I'd go out in the morning and come back and then I'd go out again, I just used to go out looking round the shops. I just get these crossword books and I do those. (Participant 20, female, age 92, 5 weeks post operation)

Participants who were active before their fracture were usually able to resume valued activities but had some limitations which remained a frustration.

> I can do little (gardening) jobs but because I haven't got as much movement in the hip joints, I find it difficult to go down on my hands and knees…If I go down on one knee it's difficult to get up again so that's not possible but I can do things that are higher up, I can trim. (Participant 15, female, age 61, 15 weeks post operation)

> I'm tackling a little bit of cooking now. I started to cook myself some nice lunches and I haven't got round to the… scones … I made one lot when I came home and I thought, I can't be bothered anymore. (Participant 10, female, age 83, 18 weeks post operation)

Some participants returned to valued activities through adapting how they did them, this participant using a wheelchair for the first time.

For peer review only

> Over the last three weeks, when we go out shopping now, I can't go down the aisles, so [daughter] gets me a (wheel)chair and I can sit in the chair and then say what shopping I need, that is very good. (Participant 9, female, age 92, 18 weeks post operation)

Participants who no longer undertook valued activities that involved significant mobility were content to continue as they were, for example, occupying themselves with visits from family and reading.

Personal care

Washing, dressing and getting to the toilet was talked about in interviews, but in many cases it was not clear whether difficulties with personal care were specifically due to the fracture. A few interviewees talked about problems with incontinence but again it was unclear whether this was specific to the fracture. Most patients had a commode or had arranged to sleep near the bathroom in the weeks immediately after the fracture. Some participants were able to describe problems with self-care specific to the hip fracture.

> I'm …not able to put a sock or anything on my injured leg. I can manage now with my trouser leg and throw these jogging trousers and hook my leg into them but I have to ask my husband if I need to put a sock or a shoe, or my slipper on that foot. (Participant 15, female, age 61, 6 weeks post operation)

At the second interview this participant was pleased to report that she now needed very little help with self-care, at least in part through wearing alternative footwear.

> I still have to throw my clothes and hook them onto the foot to get dressed. I couldn't wear lace-up shoes or anything like that because I couldn't tie them up, but things like slip-ons and sandals I can get on quite easily, so I'm fairly independent – I am independent really, I just need help with cutting my toenails and that – those on the right foot that's all. (Participant 15, female, age 61, 15 weeks post operation)

Pain

Although pain was talked about by some interviewees it was not considered a major problem.

> So here I am, four or five weeks [post operation], I get a little bit of pain, not a lot. (Participant 7, female, age 70, 5 weeks post operation)

> The pain was so bad before I had it done, and I just couldn't believe the relief after the operation when I was walking in the hospital and I had one of those pushers you know. And there was no pain. And I kept thinking, I can't believe this, and that's how it's been. I've never had any pain, not at all.
> (Participant 10, female, age 83, 18 weeks post operation)

For peer review only

> There's several times, like when I have got to get up those steps. I put my right foot first and bring my left foot up, and once or twice… you step on your left, and it's still there, lets you know it's still tender. (Participant 12, male, age 78, 16 weeks post operation)

Mental wellbeing

Low mood or depression associated with the reduced mobility due to the fracture was reported by a few interviewees, emphasising the great value placed by interviewees on being independently mobile.

> He can't walk and that, to him he'd rather die. I'll be honest with you he's said it once or twice, "Let me go". And I said, "No you're not going no-where". And then the other day for the first time, but he hasn't said it since, "I'm going to commit suicide", I said, "No you're not, you're not". (Carer of participant 31, male, age 84, 5 week post operation)

> For me it was a massive problem and caused me depression. To me is the most important thing, the mental aspect of taking away somebody's freedom to be able to move around and go to the shops and do all that sort of thing.
> (Participant 7, female, age 70, 23 weeks post operation)

Fear of falling

The experience of the fracture left a few participants with a fear of falling and sustaining a further fracture.

> I think it frightened him more than anything else. He's frightened he'll fall over again and do it again, that bothers him more than anything else. Because now when he stands up at all to try and walk he's frightened he's going to fall over and the same thing will happen all over again. (Carer of participant 11, male, age 84, 7 weeks post operation)

> I've got to watch what I'm doing. If I catch my foot on [paving stone], I can go over again. (Participant 12, male, age 78, 16 weeks post operation)

The fear of falling was sometimes expressed by a family member. When talking about his frustration at not being able to work in the garden, participant 6 added

> All the rain has made it very slippery, and [wife] says, "No way do you go out there." (Participant 12, male, age 78, 6 weeks post operation)

This emphasises the value given to stable mobility by interviewees.

Leg shortening

This is a problem that is common following extra-capsular fracture of the proximal femur. One interviewee described her concerns about this.

> One leg is now shorter than the other so that makes walking a bit difficult because it gives me back pain. (Participant 15, female, age 61, 15 weeks post operation)

For peer review only

Variation in how patients talk about recovery from a hip fracture

Our sample included patients from across a spectrum that extended from those who were physically and mentally active prior to their fracture through to those who, pre-fracture, had been immobile due to conditions such as multiple sclerosis, chronic obstructive airways disease and arthritis, and those with severe cognitive impairment. In Box 1 we present condensed versions of the interview summaries for participants chosen to represent the whole spectrum of patients. We indicate whether the data was provided by patient, carer or both.

Recovery as a return to pre-fracture state or as part of aging and decline

Every patient interviewed had experienced a hip fracture and surgery, so in physical terms all of them had, for a period of time, been somewhat impaired compared to their pre-fracture state. Four weeks post-operation, those who were active pre-fracture talked in terms of regaining a recovered state that was similar to their pre-fracture state although with some minor adaptations (participants 15 and 20 in box 1). Whilst these participants expressed worry about how well they might function in the future, there was, nevertheless, determination to progress to as full a recovery as possible. Four months post-operation many of these participants had all but regained their pre-fracture level of activity. Among participants with severely limited mobility pre-fracture, some were able to identify specific activities which were more difficult post-fracture than pre-fracture, such as putting on socks and getting in and out of bed. Some were also able to identify specific improvements in mobility post operation (see participants 9 and 15 in box 1). These participants described a process of recovery although it was very limited.

In contrast, for other participants, the fracture was just one part of a process of aging and decline. For example, participant 11 (see box 1) had been very limited in his activities before the fracture. Post fracture he needed adaptations to his home and increased care support post fracture to enable him to continue to manage at home. The mobility of participant 18 had declined and she had started using a wheelchair instead of her mobility scooter to get out of the house. However, it was unclear whether the decline was due to the concurrent heart failure or the fracture. Those who were the most physically or cognitively impaired pre-fracture did not talk about regaining a recovered state but about a state of no change. They continued with their limited activities as before (for example: participants 23 and 26 in box 1). For one participant, the only change was her move to a new nursing home (participant 5 in

For peer review only

box 1). Participants with cognitive impairment were often unaware of having experienced a fracture (Participant 1 box 1).

Recovery through adaptation

In the face of their physical limitations, most participants made adaptations that mitigated the effect of the fracture; for example employing a cleaner, moving to a nursing home or using a walking aid or other assistive device. For those who were active pre-fracture, adaptation was mostly considered temporary, although at 4 months there was some evidence that active patients had adapted to some limitations such as being unable to kneel for gardening or limiting time spent shopping to avoid exhaustion. For some participants who had been experiencing decline in their mobility pre-fracture, the fracture precipitated adaptations that they had not previously considered but made their life easier. These included using a wheelchair for shopping, having a new ramp built for getting in and out of the house in a wheelchair, using a walking aid or employing professional carers to assist with personal care. For some, their own or their carer's fear of further falls limited their mobility or at least limited how far they tested their ability to walk. Poor weather conditions exacerbated this fear, but adaptations to the environment such as walking aids or handrails lessened the fear.

Discussion

Following hip fracture, for those who had some pre-fracture mobility and able to articulate what they value during recovery, stable mobility, that is, mobility without the experience of or fear of falling, and mobility that that allows people to undertake valued activities are most valued. The ability to walk is important but so too are other leg movements needed for activities such as gardening or using transport. For some participants, maintaining mobility, however limited, was achieved by using assistive devices or working out new ways of doing an activity. Some participants adapted to their limitations, for example wearing different footwear or adjusting their expectations of what they could achieve. Others maintained their previous limited function through increased care provision.

Patients also consistently valued certain basic domains of health, such as pain (or lack of it), day-to-day activities, personal care and mental well-being. However, many participants in this study were unable to articulate what was important to them in terms of recovery from hip fracture. The hip fracture was just one part of their decline with age and its impact could not be disentangled from the impact of other health issues.. The level of recovery perceived by a

For peer review only

participant was influenced by their pre-fracture state and their ability to make adaptions during recovery.

Strengths and weaknesses of the study

When the mortality rate post operation is taken into account, including the higher mortality amongst older females, the study sample was broadly representative of the age profile and gender balance of the population of England, Wales and Northern Ireland experiencing hip fractures (2). We used a higher cut off for assessment of cognitive impairment (score of 8 on AMTS) compared to the NHFD (score of 6 on AMTS). This is likely to explain our higher proportion of participants with cognitive impairment compared to the average in the NHFD.

More research time was spent on recruitment than any other aspect of the study as it proved difficult. When contacted about the interview study, potential participants talked about other priorities or concerns that prevented them agreeing to interview, or they simply did not wish to be interviewed. It is possible that those not interviewed were struggling most with recovery. Our data is also limited by the difficulty some frail older adults have in giving a detailed account of their health experience (18). Interview data is jointly constructed by interviewer and interviewee (19) and our interviewer had no clinical knowledge of hip fractures. This reduced the likelihood of the interviewer influencing the data. A clinician undertaking the interviews would have the knowledge to help the patient tease out whether health problems were fracture related or not. However, this would have obscured the important finding, that participants often experienced their fracture as part of, rather than separate to, their other existing health problems. For those with cognitive impairment, some carers were unable to give a detailed account of recovery due to limited day-to-day contact with the participant.

Comparison with other studies

There are similarities between our findings and other qualitative studies of similar populations. A Swedish team that explored engagement with rehabilitation post hip fracture found a similar spectrum of participants (20). They classified their participants as: those who were frail and in need of support but did not request it; those who were dependent and took no active part in rehabilitation and those who were self-sufficient. Another Swedish study, undertaken with people 12 months after their hip fracture found that mobility and a return to normal activities were key outcomes for patients (21). An Australian study of mobility post-

For peer review only

fracture found that reduced level of mobility was associated with fear of falling, physical limitations from other illness and social/environmental factors (22). Our results also echo findings from across the research literature on the experience of health and illness. For example, the difficulty disentangling the impact of one health condition from other co-morbidities has been found for mental health conditions (23). The acceptance of an acute health problem as being part of the aging process has been found for conditions such as stroke (24). Recalibration to altered circumstances in response to a sudden injury has also been described (25), as have the adaptations- both physical and psychological- that people make in order to maintain their quality of life (26). Reduced expectations of health and acceptance of limited function have been described among elderly women (27). Fear of falling is common among older people generally (28). The consistency between our findings and other studies suggests that we now have sufficient qualitative evidence to inform policy decisions about the choice of appropriate PROMS for assessing recovery from hip fracture.

Implications for clinicians and policymakers

This study was undertaken in response to a potential policy change involving the use of a PROM to assess patient recovery from hip fracture, the results of which would form part of the evaluation of the quality of care provided for hip fracture. For the population experiencing fragility hip fractures, it is unlikely that a single PROM specific to hip fracture could be developed which is relevant to the whole spectrum of patients. Several of the themes described by our more active interviewees - mobility, day-to-day activities, self-care, pain and mental wellbeing, are similar to the domains included in currently available generic measures including the EuroQoL EQ-5D (13), the Short Form 36-item Health Survey (SF-36) (29) and the WHOQoL-BREF (30). Both the EQ-5D (3L) and the SF-36 (version 1) have been widely used in trials of people sustaining hip fractures, but for both measures evidence of essential measurement and practical properties is limited (7). In the context of a clinical trial where patients are randomised to an intervention and control arm, these measures may be appropriate but they may need to be supplemented by specific tools for selected groups, especially those patients with high-levels of pre-injury function,

In the context of assessing quality of care for a patient population as diverse as those experiencing hip fracture, it may be impossible to devise a single PROM that will be appropriate for all patients. Although quality of care may be one factor that will influence recovery as perceived by a patient, their pre-fracture state, adaptations that they or their carers

For peer review only

make to their reduced mobility, and their perception of whether or not they are at the stage in life where decline is inevitable will all influence how they answer questions contained within a PROM.

For peer review only

Figure 1 Flow chart of study recruitment

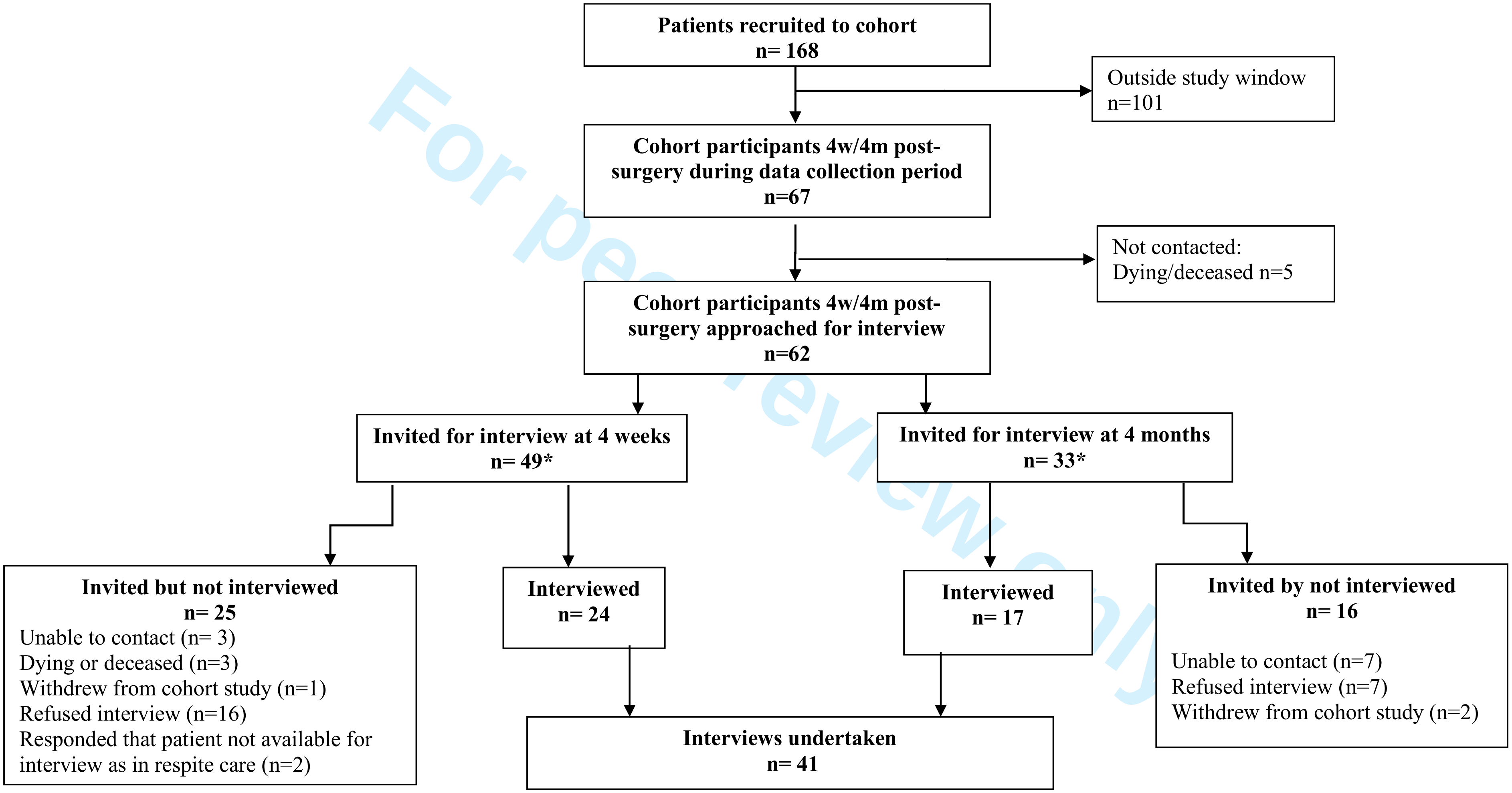

Notes: * 20 participants were invited for interview at both 4 weeks and 4 months post operation

For peer review only

For peer review only - http://bmjopen.bmj.com/site/about/guidelines.xhtml

61 year old female social worker who lives with her husband. Before her fracture she was working full time and, for recreation, taking country walks, undertaking all types of gardening activities and playing with her grandchildren. Post fracture fixation (6 weeks) she described using crutches to get around the garden and shops, needing help with putting on socks and cutting toe nails, and was unable to climb stairs. She talked in terms of improvement and expectation of returning to work and full activity including cleaning and gardening. By the second interview she was frustrated that recovery was so slow but she could identify the ways in which she had continued to recover. (Participant 15, interviewed 6 weeks and 15 weeks post operation)

92 year old female who lives alone in her own flat within a sheltered housing complex. Prior to the hip fracture she looked after herself and did her own washing, but had a cleaner to undertake heavy household chores. She spent most of each day out and about at the shops, engaging in social activities, bingo and on outings. She had no other illnesses. Post-fracture fixation she talked about having some initial pain and problems lifting her leg after the operation but was now mobile about her home with a walking frame. The housing complex has a lift which she now used. She was intending to return to getting out and about as she was before her fracture. (Participant 20, interviewed 5 weeks post operation)

92 year old female lives alone with husband. Daughter visits several times a week to help. Poor hearing. Difficult to disentangle what was before and after fracture. Seems to have been able to walk around house, undertake self-care and microwave own meals pre-fracture. Post fixation of the hip fracture, patient slowly improved walking. Life seems very similar to before fracture except need for walking aid, inability to put on socks and husband now microwaves the meals. (Participant 9, interviewed 9 weeks post operation)

70 year old male retired painter and decorator who lives with his wife and enjoys almost daily visits from his grandchildren. Mobility restricted to 5-6 metres for more than two years prior to fracture due to knee pain and chronic obstructive pulmonary disease. When interviewed he describes struggling to get up the stairs, get in and out of bed, put his shoes and socks on, and bend down. Although his mobility was severely restricted prior to his fracture, he described being unable get around as much as he had done before the fracture. He noted some improvement over recent weeks, as he no longer needed two sticks for walking, only one. (Participant 3, interviewed 15 weeks post operation)

84 year old male with dementia, who has some lucid moments and some recall of falling and hurting himself. He lives with his wife who looks after him and they have a cleaner to do heavy housework. Wife provided interview, involving the patient in the latter half when he woke up. Patient's walking was gradually slowing and he had a number of falls before his fracture. Fracture occurred while walking in shopping area with his wife. Since fixation of the fracture patient has required assistance with personal care, has professional carers four times a day, and the bathroom has been adapted for his limited mobility. The interviewee had difficulty distinguishing decline due to old age and change due to the fracture. The patient complained of some pain but it was unclear whether this was from the fracture or previously established osteoarthritis. Before the fracture both patient and wife had ceased all non-essential activities except for a weekly trip to the shops so daily life had changed little except for more care provision. (Participant 11, interviewed 7 weeks post operation)

74 year old female who lives with husband. Patient lived with severe rheumatoid arthritis for

For peer review only

30 years. Developed heart failure and admitted to hospital with shortness of breath and confusion. Fell while in hospital and fractured her hip. Mobility before hip fracture very limited – able to walk slowly in house and garden, undertake light chores, and use scooter to go shopping. Became worse with breathing difficulty. Mobility remained reduced after hospital admission. Able to take step slowly in house with support. Uses wheelchair to go out of house – a new ramp improved this by second interview. Unclear how much mobility change was due to the fracture and how much due to heart failure. (Participant 18, interviewed 6 weeks and 18 weeks post operation)

88 year old female retired teacher, who lives with her son and has a diagnosis of multiple sclerosis. The patient wove together pre and post injury experience in her account, making it difficult to disentangle. She said her son does the cooking and cleaning and her daughter assists with self-care. She has a close family, feels well supported and has lots of visitors – friends, grandchildren and great grandchildren. Her main interest beyond seeing friends and family is reading. She described being content with life. Prior to her fracture she was unwell with an infection and recounts using a frame for mobility which she still uses. (Participant 23, interviewed 5 weeks post operation)

85 year old female living in a nursing home. Her daughter visits alternate days. Her daughter provided the interview data. The patient has dementia but otherwise had been well before the fracture. Patient gets up and walks about herself, and takes herself to the toilet. She enjoys sitting and chatting. The patient does not remember the injury. Her life has not changed from how it was pre injury. The daughter did not mention any fracture-specific issues related to recovery. (Participant 26, interviewed 6 weeks post operation)

84 year old female with limited English language. Pre-injury she had carers to assist her with all her personal needs. The injury had occurred whilst being hoisted. Post injury her main concern was that at discharge from hospital, after a three month stay, she was sent to a nursing home where she knew no-one. The patient repeatedly expressed distress about being in the nursing home but did not talk about the fracture.
(Participant 5, interviewed 18 weeks post operation)

84 year old male who has dementia. He lives alone but received visits three times a day from his son who provides meals. Son was interviewed. Arthritis of knee limited mobility before the fracture. Spent most of the day sitting. At weekends prior to fracture patient went to neighbour's house for evening meal. Patient fell and sustained fracture while walking to neighbour's house. Patient does not recall fracture. At time of interview, the patient was as mobile as pre operation limited by pain and stiffness from arthritis. Not yet visiting neighbour but this was because family was discouraging this in case he falls again rather than due to mobility. (Participant 1, interviewed 16 weeks post operation)

Box 1 Summaries of the data about individual patients and their recovery from a hip fracture

For peer review only

Competing interest statement
All authors have completed the Unified Competing Interest form at www.icmje.org/coi_disclosure.pdf (available on request from the corresponding author) and declare: funding from the National Institute of Health Research, University of Warwick, and University Hospitals Coventry and Warwickshire NHS trust.

Authorship
MC, FG, JA, XG, KH and FB contributed to the conception and design of the study. All authors contributed to analysis and interpretation of data. FG, KD and VM drafted the article and all authors revised it critically for important intellectual content. All authors gave final approval of the version to be published.

Acknowledgements
We thank Katie McGuinness, Kate Dennison, Zoe Buckingham, Catherine Richmond, Rebecca McKeown, Hayley Rice, Mamta Rana, Filo Eales and Gail McCloskey for their assistance in recruitment and all the patients for their time and effort in participating in this study.

Funding
This study was funded by a Programme Development Grant from the National Institute of Health Research (RP-DG-1210-10022). This trial was co-sponsored by the University of Warwick and University Hospitals Coventry and Warwickshire NHS trust. This manuscript presents independent research. The views expressed are those of the authors.

Ethical approval
Ethical approval was granted by NHS REC London - Camberwell and St Giles (11/LO/0927) on the 18th August 2011. Further approval was obtained from the research and development department of the University Hospitals Coventry and Warwickshire NHS Trust. This research complies with the Helsinki Declaration.

Access to study data
All authors had full access to all of the data in the study and can take responsibility for the integrity of the data and the accuracy of the data analysis

Data sharing: no additional data available

For peer review only

References

1. Johnell O, Kanis JA. An estimate of the worldwide prevalence, mortality and disability associated with hip fracture. . Osteoporosis international: a journal established as result of cooperation between the European Foundation for Osteoporosis and the National Osteoporosis Foundation of the USA. 2004;15:897-902.
2. Johansen A, Wakeman R, Boulton C, Plant F, Roberts J, Williams A. National Hip Fracture Database National report 2013. Clinical Effectiveness and Evaluation Unit at the Royal College of Physicians, 2013.
3. Department of Health. Update to Best Practice Tariffs: Department of Health; 2011 [5th November 2012]. Available from: http://www.dh.gov.uk/health/2011/12/bpt-update/.
4. Devlin N, Appleby J. Getting the Most Out of PROMs. Putting health outcomes at the heart of NHS decision-making. London: The King's Fund, 2010.
5. Patrick DL, Burke LB, Powers JH, Scott JA, Rock EP, Dawisha S, et al. Patient-reported outcomes to support medical product labeling claims: FDA perspective. Value Health. 2007;10:S125-S37.
6. Fitzpatrick R. Patient-reported outcome measures and performance measurement. In: Smith P, Mossialos E, Papnicolas I, Leatherman S, editors. Performance measurement for health system improvement: experiences, challenges and prospects. Cambridge: Cambridge University Press; 2009.
7. Haywood K, Brett J, Tutton E, Staniszewska S, editors. How well are we measuring patient-centred outcomes following traumatic hip fracture? A systematic review of patient-reported outcome measures (PROMs). Royal College of Nursing International Research Conference; 2012 23-25th April 2012; London: Royal College of Nursing.
8. Griffin X, Achten J, Parsons N, Boardman F, Griffiths F, Costa M. The Warwick Hip Trauma Evaluation - an abridged protocol for the WHiTE Study. A multiple embedded randomised controlled trial cohort study. . Bone Joint Res. 2012;1(11):310-4.
9. Hodkinson HM. Evaluation of a mental test score for the assessment of mental impairment in the elderly. Age and Ageing. 1972;1(4):233-8.
10. MacKenzie DM, Copp P, Shaw RJ, Goodwin GM. Brief cognitive screening of the elderly: a comparison of the Mini-Mental State Examination (MMSE), Abbreviated Mental Test (AMT) and Mental Status Questionnaire (MSQ). Psychol Med. 1996;26(2):427-30.
11. Mental Capacity Act 2005, (2005).
12. Currie C, Fleming S, Partridge M, Plant F, Wakeman R, Williams A. National Hip Fracture Database National Report. 2010.
13. The EuroQoL Group. EuroQoL: a new facility for the measurement of health-related quality of life. Health Policy. 1990;16(3):199-208.
14. McConnell-Henry T, Chapman Y, Francis K. Husserl and Heidegger: Exploring the disparity. International Journal of Nursing Practice. 2009;15(1):7-15.
15. Jacelon C. Older adults' participation in research. Nurse Researcher. 2007;14(4):64-73.
16. NVivo qualitative data analysis software. 10 ed. Melbourne, Australia: QSR International Pty Ltd; 2012.
17. Borkan J. In: Crabtree BF, Miller WL, editors. Doing Qualitative Research. Second ed. London: Sage Publications; 1999.
18. Kvigne K, Gjengedal E, Kirkevold M. Gaining access to the life-world of women suffering from stroke: methodological issues in empirical phenomenological studies. Journal of Advanced Nursing. 2002;40(1):61-8.
19. Mishler E. Research interviewing: Context and narrative. Cambridge, MA: Harvard University Press; 1986.

20. Olsson LE, Nystrom AE, Karlsson J, Ekman I. Admitted with a hip fracture: patient perceptions of rehabilitation. J Clin Nurs. 2007;16(5):853-9.
21. Ziden L, Scherman MH, Wenestam CG. The break remains - elderly people's experiences of a hip fracture 1 year after discharge. Disabil Rehabil. 2010;32(2):103-13.
22. Taylor NF, Barelli C, Harding KE. Community ambulation before and after hip fracture: a qualitative analysis. Disabil Rehabil. 2010;32(15):1281-90.
23. Connell J, Brazier J, O'Cathain A, Lloyd-Jones M, Paisley S. Quality of life of people with mental health problems: a synthesis of qualitative research. Health and Quality of Life Outcomes. 2012;10:138.
24. Faircloth CA, Boylstein C, Rittman M, Young ME, Gubrium J. Sudden illness and biographical flow in narratives of stroke recovery. Sociology of Health and Illness. 2004;26(2):242-61.
25. Bury M. Chronic illness as biographical disruption. Sociology of Health and Illness. 1982;4(2):169-82.
26. Clarke P, Black S. Quality of Life Following Stroke: Negotiating Disability, Identity, and Resources. Journal of Applied Gerontology. 2005;24:319-36.
27. Chambers P. Getting Beyond ‘Mustn't Grumble’ and ‘What Can I Expect At My Age’: A Narrative Approach to Older Women’s Health Stories. In: Narrative, Memory and Health. Huddersfield: University of Huddersfield; 2003. p. 127-39.
28. Scheffer AC, Schuurmans MJ, van Dijk N, van der Hooft T, de Rooij SE. Fear of falling: measurement strategy, prevalence, risk factors and consequences among older persons. Age and Ageing. 2008;37(1):19-24.
29. Ware Jr. J, Gandek B. Overview of the SF-36 Health Survey and the International Quality of Life Assessment (IQOLA) Project. Journal of clinical epidemiology. 1998;51(11):1203-14.
30. Molzahn A, Kalfoss M, Schick Makaroff K, Skevington S. Comparing the importance of different aspects of quality of life to older adults across diverse cultures. Age Ageing. 2011;40(2):192-9.

For peer review only

~~The assessment of health care quality~~Evaluating recovery following hip fracture: a qualitative study of what is important to patients ~~during recovery~~

Frances Griffiths, Professor of Medicine in Society, Warwick Medical School, University of Warwick, Coventry, CV4 7AL, UK

Victoria Mason, Research Fellow, Warwick Medical School, University of Warwick, Coventry, CV4 7AL, UK

Felicity Boardman, Mildred Blaxter Post-Doctoral Fellow, Warwick Medical School, University of Warwick, Coventry, CV4 7AL, UK

Kathyrn Dennick, Research ~~Fellow, Warwick Medical School, University of Warwick, Coventry, CV4 7AL, UK~~Associate, Florence Nightingale School of Nursing and Midwifery, King's College London, SE1 8WA

Kirstie Haywood, Senior Research Fellow, Royal College of Nursing Research Institute, University of Warwick, CV4 7AL, UK

Juul Achten, Research Manager Warwick Orthopaedics, Warwick Medical School, University of Warwick, Coventry, CV4 7AL, UK

Nick Parsons, Senior Research Fellow, Statistics and Epidemiology, Warwick Medical School, University of Warwick, Coventry, CV4 7AL, UK

Xavier Griffin, Specialist Trainee Trauma and Orthopaedic Surgery, Warwick Medical School, University of Warwick, Clinical Sciences Building, Clifford Bridge Road, Coventry, CV2 2DX, UK

Matt Costa, Professor Trauma and Orthopaedics, Warwick Clinical Trials Unit, Warwick Medical School, University of Warwick, Coventry, CV4 7AL, UK

Correspondence to: F.E.Griffiths@warwick.ac.uk

Formatted: Line spacing: Multiple 1.15 li

For peer review only

Formatted: English (U.K.)

Copyright
The Corresponding Author has the right to grant on behalf of all authors and does grant on behalf of all authors, an exclusive licence (or non exclusive for government employees) on a worldwide basis to the BMJ Publishing Group Ltd to permit this article (if accepted) to be published in BMJ editions and any other BMJPGL products and sublicences such use and exploit all subsidiary rights, as set out in our licence.

Formatted: English (U.K.)

Competing interest statement
All authors have completed the Unified Competing Interest form at www.icmje.org/coi_disclosure.pdf (available on request from the corresponding author) and declare: funding from the National Institute of Health Research, University of Warwick, and University Hospitals Coventry and Warwickshire NHS trust.

Formatted: English (U.K.)

Formatted: English (U.K.)

Formatted: English (U.K.)

Authorship
MC, FG, JA, XG, KH and FB contributed to the conception and design of the study. All authors contributed to analysis and interpretation of data. FG, KD and VM drafted the article and all authors revised it critically for important intellectual content. All authors gave final approval of the version to be published.

Formatted: English (U.K.)

Acknowledgements
We thank Katie McGuinness, Kate Dennison, Zoe Buckingham, Catherine Richmond, Rebecca McKeown, Hayley Rice, Mamta Rana, Filo Eales and Gail McCloskey for their assistance in recruitment and all the patients for their time and effort in participating in this study.

Formatted: English (U.K.)

Funding
This study was funded by a Programme Development Grant from the National Institute of Health Research (RP-DG-1210-10022). This trial was co-sponsored by the University of Warwick and University Hospitals Coventry and Warwickshire NHS trust. This manuscript presents independent research. The views expressed are those of the authors.

Formatted: English (U.K.)

Formatted: English (U.K.)

Formatted: English (U.K.)

Ethical approval
Ethical approval was granted by NHS REC London - Camberwell and St Giles (11/LO/0927) on the 18th August 2011. Further approval was obtained from the research and development department of the University Hospitals Coventry and Warwickshire NHS ~~trust~~Trust. This research complies with the Helsinki Declaration.

Formatted: English (U.K.)

Access to study data
All authors had full access to all of the data in the study and can take responsibility for the integrity of the data and the accuracy of the data analysis

Data sharing: no additional data available

Formatted: English (U.K.)

For peer review only

Abstract

Formatted: Font: Not Bold

Objective

To explore what patients who have recently experienced a fracture of the proximal femur (hip fracture) consider important when evaluating their recovery from hip fracture and to consider how these priorities could be used in the evaluation of the quality of hip fracture services.

Design

Semi-structured interviews exploring the experience of recovery from hip fracture at two time points - four weeks and four months post-operative hip fixation. Two approaches to analysis: thematic analysis of data specifically related to recovery from hip fracture; extraction of data into template to capture the participant's experience overall; thematic analysis of data specifically related to recovery from hip fracture.

Participants

31 participants recruited, of whom 20 were female and 12 were cognitively impaired. Mean age was 81.5 years. Interviews were provided by 19 patients with hip fracture, 14 carers, and 8 patient/carer dyad; 10 participants were interviewed twice.

Setting

Participants recruited from a singleSingle major trauma centre in the West Midlands of the UK.

Results

Active and frail patients talk about the experience of recovery from a hip fracture very differently. Active patients provided detailed descriptions of their recovery and what was important to them. For frail patients, all their health problems were part of one experience and they were unable to separate out what was due to the fracture and what was due to other conditions. Active patients saw recovery as a return to their pre-fracture state whereas for frailer patients the fracture was considered part of their decline with age. Post fracture, many patients made adaptations to their daily living. Among those able to articulate what was important to them during recovery the following themes were most prominent: mobility, return to activities, self -care, pain, mental wellbeing and fear of falling.

Stable mobility for valued activities was considered most important by participants who had some pre-fracture mobility and were able to articulate what they valued during recovery. Mobility was important for managing personal care, for day-to-day activities such as shopping and gardening, and maintenance of mental well-being. Some participants used

assistive mobility devices or adapted to their limitations. Others maintained their previous limited function through increased care provision. Many participants were unable to articulate what they valued as hip fracture was perceived as part of their decline with age. The fracture and problems from other health conditions were an inseparable part of one health experience.

Conclusions

There is no evidence that frail and active patients consider the same outcomes as important. Adaptation by the patient complicates assessment of recovery from hip fracture including using assistive devices, recalibration of expectations of health, protective response of carers and changes to the patient's environment.

Patients consistently valued stable mobility and its role in other basic health domains. While no one patient-reported outcome measure (PROM) could consistently evaluate recovery for all patients with hip fracture, general health-related quality of life tools may provide useful information for the majority of patients. These may need to be supplemented by specific tools for selected groups, especially those patients with high-levels of pre-injury function.

Key words: Hip fractures, Outcome assessment (Health Care), Interview, Frail older adults

Article summary

Article focus

- The UK NHS has identified the need to evaluate service provision for patients with a hip fracture
- There is increasing expectation that patient--reported outcomesoutcome measures (PROM) are used within health service evaluation
- We asked the question: what do patients who have recently experienced a hip fracture consider important when evaluating their recovery?

Key messages

- Patients active before their fracture consider recovery as return to their pre-fracture state whereas more frailvalue stable mobility to undertake valued activities but many patients consider fracture to be part of their decline with age.
- It would be possible to develop a PROM based on what active patients articulate as important during recovery but there is no evidence that frail and active patients consider the same outcomes as importantWhile no one PROM could evaluate all aspects of recovery for patients with hip fracture, general health-related quality of life tools may provide useful information for the majority of patients.

Formatted: Font color: Black

Formatted: Indent: Left: 0.5", No bullets or numbering

Strengths and limitations

- The study sample was representative of the age profile, gender balance and dementia levels of ~~the UK population~~NHS patients experiencing hip fractures
- It is possible that those not agreeing to be interviewed were struggling most with recovery.
- The data is limited by the difficulty ~~some frail older adults have~~the more physically and cognitively impaired patients had in giving a detailed account of their health experience.

For peer review only

Introduction

Fragility fracture of the proximal femur (hip fracture) is one of the greatest challenges facing the healthcare community. In 1990, a global incidence of 1.31 million was reported and was associated with 740,000 deaths (1). Hip fractures constitute a heavy socioeconomic burden worldwide. The cost of this clinical problem is estimated at 1.75 million disability adjusted life years lost; 1.4% of the total healthcare burden in established market economies (1). Among those experiencing fragility hip fracture in England, Wales and Northern Ireland, 70% are aged 80 years or older, 73% are female and 34% are cognitively impaired pre-operation. The mortality rate within 30 days of operation was 8.2% in 2013 (2)

The NHS has identified the need to evaluate the quality of service provision for patients with a hip fracture; this evaluation is conducted through the National Hip Fracture Audit Database (NHFD)(2).(2). Currently, aspects of care such as time to surgery, length of patient stay and patient mortality in hospital and 30 day and 120 day follow up are recorded in the NHFD. Furthermore, theseThese data are now used to guide payments to healthcare providers; the payment being increased if the provider supplies 'best practice' care (3). However, while important, we suggest thatthere is interest from policy makers in the potential to enhance these currently reported data fields areby including and an incomplete assessment of outcome.

as reported by patients. It is increasingly expected that healthcare evaluations should include domains of health that are important to patients, (4), captured by well-developed patient-reported outcome measures (PROMs) which aim to assess how patients function and feel in relation to a health condition or associated treatment (5). PROMS capture information that cannot be obtained by other means (5, 6) complementing more traditional performance or process-based measures. However, for

Our aim was to establish whether or not one PROM could be used with all patients who have sustainedexperience a fragility hip fracture, guidance for PROM-based assessment is not available, as part of the evaluation of the quality of health care for hip fracture delivered by the NHS. For this patient group we were unable to identify a PROM specific to the assessment of hip fractures does not existfracture, and robust evidence of the quality and acceptability of non-hip fracture specific PROMs following completion by patients sustaining

a hip fracture is limited (7). Moreover, clarity with regards to the outcomes of healthcare that ~~this group of~~these patients considers relevant and important does not exist. Appropriate and relevant PROM-based assessment ~~must~~should be underpinned by an understanding of ~~what's~~what is important to patients in terms of the outcomes of healthcare~~ following a hip fracture. ~~. We therefore designed an interview study to explore with patients and, where appropriate, their carers, what they consider to be important outcomes and to explore variation across this patient group. Our research questions were:

1. ~~In response to these gaps in understanding, we proposed the following research question:~~ What do patients who have recently experienced a hip fracture consider important when evaluating their recovery?~~ To answer this question, we undertook an interview study with patients and, where appropriate, their carers to explore the key outcomes of healthcare that they regarded as important following a hip fracture, and which may inform subsequent PROM-based assessment.~~
2. Is there variation within this population about what is considered important?

These research questions are framed by the desire of policy makers to evaluate the quality of care for hip fracture through assessment of recovery from the perspective of the patient.

Formatted: List Paragraph, Indent: Left: 0", Numbered + Level: 1 + Numbering Style: 1, 2, 3, … + Start at: 1 + Alignment: Left + Aligned at: 0.25" + Indent at: 0.5"

Method

Study Design

~~Semi~~We conducted semi-structured interviews ~~were conducted~~ with patients and, where appropriate, their carers at two time points, at approximately four weeks and then again at four months after ~~sustaining~~they had sustained a fragility hip fracture~~ of the hip~~.

Identification of patients with a hip fracture

~~Participants were~~We recruited participants from an existing cohort study, the Warwick Hip Trauma Evaluation (8), that commenced January 2012. This is a cohort of all patients admitted with a hip fracture to a single major trauma centre in the West Midlands of the United Kingdom. As part of their pre-operative assessment, patients were assessed for their capacity to consent using clinical assessment and the Abbreviated Mental Test Score (AMTS) (9). The AMTS is a 10-item measure used to rapidly assess the possibility of cognitive impairment in elderly people. A score below 8 suggests cognitive impairment (10). Scores less than 8 were taken to indicate that a patient was unlikely to be able to consent for

For peer review only

themselves. Those deemed to have capacity for consenting to surgery, based on clinical assessment and AMTS, were considered able to consent for this study. Following the emergency surgery for their fracture, those with capacity gave written consent to be approached for interview. ~~For those deemed not to have capacity due to cognitive impairment, verbal consent was obtained from their consultee (11).~~For those deemed not to have capacity due to cognitive impairment, verbal consent was obtained from their consultee (11). Ethical approval was granted by NHS REC London - Camberwell and St Giles (11/LO/0927) on the 18th August 2011.

Sampling

During the data collection period for this study, February to August 2012, we purposefully sampled cohort participants who had reached 4 weeks or 4 months following their hip fracture and had consented to be approached for interview. The time points were chosen to be the same as those used for data collection for the NHFD (12). ~~The~~If a PROM were to be used with this patient population to assess quality of care, patients would be asked to complete the PROM at these time points. Our sampling strategy ensured a diverse mix of patients with respect to the following factors: age, gender, AMTS (9) and EQ-5D score (13).

Interview recruitment and consent process

~~Eligible~~We contacted eligible patients and carers ~~were contacted~~ just prior to 4 weeks and/or 4 months following hip fracture to arrange an interview. If patients declined to participate, the reasons offered were recorded. Patients with capacity to consent were contacted directly. For those patients deemed not to have capacity, we contacted their consultee ~~was contacted. Where patients were~~ . Patients able to consent for themselves ~~they~~ signed their own consent forms. For those unable to consent the consultee signed an agreement form~~. For these patients~~ and we aimed to interview a carer as well as the patient (patient/carer dyad~~) and for these interviews the carer~~). Carers who were interviewed signed a consent form. The study flow diagram is at Figure 1.

Interview process

~~Participants were~~We interviewed participants at their current residence (own home, residential or nursing home) or in hospital. ~~Interviews were undertaken by a researcher who~~The interviewer was trained in interviewing but did not have clinical knowledge of hip fracture, its treatment or prognosis. Where possible, patients and carers were interviewed

For peer review only

alone, however where the carer and patient requested a joint interview (whether or not the patient had cognitive impairment) they were interviewed together. The semi-structured interviews at both time points focused on the experience of the fracture and the patient's recovery.), they were interviewed together. The aim of the interviews was to understand each participant's lived experience of hip fracture (14) and the influence of their social context and pre-fracture health. The interviewer encouraged participants to talk about the experience in whatever order they chose and using terms meaningful to them. AsLater in the interview proceeded, the interviewerwe prompted participants to clarify , where necessary, for clarification about what in the patient experience was ablerelated to do just before the hip fracture, what was changing during their recovery, and. Towards the end of the interview we directly asked what was important to them in terms of recovery. Where the participants if this had not already been talked about health issues,by the interviewer prompted them to clarify whether these related to the fracture. During the interview process, considerationparticipant. Consideration was given to the potential challenges associated with interviewing older adults, for example by giving potential participants sufficient time to decide whether or not to participate and minimising the burden and fatigue through streamlining questions (14). During the initial stage of data collection the interview process, questions and prompts were refined by the study team using a process of iterative review and adjustment.(15). The interview process, questions and prompts were refined by the study team during the initial stage of data collection. Interviews were audio recorded and transcribed verbatim. For one interview, audio recording was not feasible due to the noisy environment so extensive field notes were taken and transcribed.

Formatted: English (U.S.)

Analysis

Interviews transcripts were checked, anonymised and uploaded into NVivo software (15). Initial analysis involved data immersion, reading and re-reading the transcripts, and discussion of interview transcripts by the research team. All team members read at least five transcripts. Issues and themes crystallised from this process (16). For data collection and analysis we took a phenomenological approach in that we sought to understand participant's experience of hip fracture and the influence of their context on this (17) and concurrently we took a selective realist position (18) in that we recognised hip fracture as an event identifiable by means other than through the participant's account.

For peer review only

Some interviewees had difficulty articulating anything about their fracture and their interviews contained almost no data that could be clearly identified as related to the fracture itself. In many interviews the interviewees talked mostly about general health issues and required considerable prompting to clarify what was related to their fracture. We therefore followed two different analysis processes reflecting our dual theoretical approaches. The first captured the overall sense of the interviewee's experience and how they talked about it, and the second focused on data specifically related to recovery from the hip fracture.

For the first analysis, through comparison of the transcripts Interview transcripts were checked, anonymised and uploaded into Nvivo software (16). Initial analysis involved data immersion, reading and re-reading each transcript, discussion of the interview transcripts by the research team. All team members read at least five transcripts. The key issues crystallised from this process (17). We found that the interviews at four weeks and four months covered very similar issues, although at four months reporting of fracture specific recovery was more advanced. For analysis, we therefore treated all the interviews related to one participant as one set of data. During data interpretation we took account of whether the interview data was from a patient or carer or patient/carer dyad. Two different approaches to analysis were then undertaken in response to our research questions.

To answer our first research question, we searched the transcripts for any mention of what was important during recovery from hip fracture and coded this text. As coding proceeded, we reviewed these codes and combined them into themes. After coding ten transcripts no additional themes were identified in the data. Double coding was undertaken for one in four transcripts and coding compared and discussed.

To answer our second research question, from close reading of the interview transcripts, we developed and refined a template for summarising the key issues of relevance to recovery from the hip fracture based on the interview data and refined the template after using it with four interviews. The template included: current and recent past living arrangements and environment, day-to-day life now and in the recent past, the impact of the hip fracture and its management, what was changing in day-to-day life as they recovered, the extent to which the patient referred specifically to the fracture and their ability to engage in the interview. The data from each patient or patient/carer dyad was summarised with a second research team member reviewing each summary against the data. The summaries were then compared.

Results

Twenty one patients were interviewed on one occasion and 10 were interviewed twice giving a total of 31 patient participants and 41 interviews. Of the 31 patient participants, 20 (64.5%) were female, the mean age was 81.5 years (SD 9.2, range 61-96) and 12 scored less than eight on the AMTS. Of the 41 interviews, 24 were conducted three to nine weeks, and 17 were conducted 14 to 23 weeks after the hip fracture. ~~One team member then extracted and summarised into the template the data from each patient or patient/carer dyad. The wider team reviewed the extractions with the transcripts. This analysis involved very close reading of the interviews and piecing the data together from across all of the interviews related to each patient. For the second analysis, we searched the transcripts for any mention of what was important during recovery from the hip fracture and coded this text. The coding framework was reviewed and finalised after coding ten transcripts. Double coding was undertaken for one in four transcripts. The whole team reviewed the extracted data and the coded data. As analysis proceeded we found that the interviews at four weeks and four months covered very similar issues, although at four months fracture specific recovery, where this was articulated, was more advanced. We therefore treated the interviews from each time point as one dataset for analysis. In the four month interviews we found that the ability of participants to articulate hip specific recovery was even less than in the interviews undertaken at four weeks.~~Nineteen interviews were with the patient only, 14 with carer only, and eight with patient/carer dyads. Despite framing the interview for interviewees as exploring the experience of hip fracture, many interviewees talked about general health issues. Although we prompted to clarify what was related to their fracture, in many interviews it was difficult to disentangle the impact of the fracture from the impact of other health problems. Some interviews contained almost no data that was clearly related to the fracture. From the perspective of the patient, all their health problems were part of one experience. The absence of data clearly related to the fracture was more marked in the four month compared to two month interviews. We therefore decided not to attempt interviews at 12 months post fracture as originally planned (8). ~~During~~ The following sections report our analysis. Illustrative quotations from data ~~interpretation we took account~~are labelled with the age and gender of the patient, time since hip fracture and whether the ~~interview data~~quotation was from ~~a~~the patient or carer ~~or patient/carer dyad~~.

What is important to patients when evaluating their recovery

For peer review only

From our systematic search of the interviews for data related to recovery from the hip fracture we identified the following themes: mobility, valued day-to-day activities, self-care, pain, mental wellbeing, fear of falling and leg shortening. When talking about mobility, day-to-day activities or self-care participants also talked about their level of independence.

Mobility

This was the most prominent theme, although when talking about mobility the interviewees often mentioned other themes. Mobile participants reported limited mobility in the weeks post operation and valued any improvement.

> I'm walking with a walking stick at the moment. I've been down the park and back…I can usually get around [the house] without the walking stick, and I can get up and down stairs no problem. I get upstairs with my good leg and downstairs with my bad leg. (Participant 6, male, age 78, 5 weeks post operation)

By four months, for many participants mobility had improved, and they were happy that they were returning to normal mobility.

> I can't rush round like I did, but eventually that will come…I mean it's pretty normal now, but I think it's going to be a while before I can actually walk as I did and I probably won't walk as I did… when I came home [from hospital] I was still hobbling... but now I'm more or less…walking normal, especially with the stick (Participant 10, female, age 83, 18 weeks post operation)

For those with limited mobility before hip fracture any unaided improvement was limited to the pre-fracture level but also valued.

> The operation was successful and got him back to normal right from the start, right from the very first day that he had it done. He was able to then walk pain free with a Zimmer frame to the toilet. The staff were all saying it was amazing how well he was walking and he would soon be back to normal, but what they didn't realise was that he was walking normally. (Carer of participant 1, male, age 84, 16 weeks post operation)

Other participants were using mobility aids that they had not been using regularly before the fracture. For some, the addition of mobility aids enabled greater security of mobility than prior to their fracture.

> Her mobility's getting better. I think she'll cope with the frame. She's had a couple of falls in the home, earlier when she was forgetting that she had to use the frame. She'd get out of bed and not use the frame and consequently fall. But she's got in the habit of using it now… she's not falling, which is a bonus. (Carer of participant 13, female, age 87, 14 weeks post operation)

For peer review only

Valued day-to-day activities

Those who were active prior to their fracture talked about the frustration of the restriction in their activities particularly in the weeks following the fracture.

> I'm back on what I call domestic duties – washing up! But the thing that is frustrating is that I can't get outside and do any gardening. (Participant 12, male, age 78, 6 weeks post operation)

> I just miss getting up and getting out. I never stayed in. I'd go out in the morning and come back and then I'd go out again, I just used to go out looking round the shops. I just get these crossword books and I do those. (Participant 20, female, age 92, 5 weeks post operation)

Participants who were active before their fracture were usually able to resume valued activities but had some limitations which remained a frustration.

> I can do little (gardening) jobs but because I haven't got as much movement in the hip joints, I find it difficult to go down on my hands and knees…If I go down on one knee it's difficult to get up again so that's not possible but I can do things that are higher up, I can trim. (Participant 15, female, age 61, 15 weeks post operation)

> I'm tackling a little bit of cooking now. I started to cook myself some nice lunches and I haven't got round to the… scones … I made one lot when I came home and I thought, I can't be bothered anymore. (Participant 10, female, age 83, 18 weeks post operation)

Some participants returned to valued activities through adapting how they did them, this participant using a wheelchair for the first time.

> Over the last three weeks, when we go out shopping now, I can't go down the aisles, so [daughter] gets me a (wheel)chair and I can sit in the chair and then say what shopping I need, that is very good. (Participant 9, female, age 92, 18 weeks post operation)

Participants who no longer undertook valued activities that involved significant mobility were content to continue as they were, for example, occupying themselves with visits from family and reading.

Personal care

Formatted: Font: Italic

Washing, dressing and getting to the toilet was talked about in interviews, but in many cases it was not clear whether difficulties with personal care were specifically due to the fracture. A few interviewees talked about problems with incontinence but again it was unclear whether this was specific to the fracture. Most patients had a commode or had arranged to sleep near

For peer review only

the bathroom in the weeks immediately after the fracture. Some participants were able to describe problems with self-care specific to the hip fracture.

> I'm …not able to put a sock or anything on my injured leg. I can manage now with my trouser leg and throw these jogging trousers and hook my leg into them but I have to ask my husband if I need to put a sock or a shoe, or my slipper on that foot. (Participant 15, female, age 61, 6 weeks post operation)

At the second interview this participant was pleased to report that she now needed very little help with self-care, at least in part through wearing alternative footwear.

> I still have to throw my clothes and hook them onto the foot to get dressed. I couldn't wear lace-up shoes or anything like that because I couldn't tie them up, but things like slip-ons and sandals I can get on quite easily, so I'm fairly independent – I am independent really, I just need help with cutting my toenails and that – those on the right foot that's all. (Participant 15, female, age 61, 15 weeks post operation)

Pain

Formatted: Font: Italic

Although pain was talked about by some interviewees it was not considered a major problem.

> So here I am, four or five weeks [post operation], I get a little bit of pain, not a lot. (Participant 7, female, age 70, 5 weeks post operation)

> The pain was so bad before I had it done, and I just couldn't believe the relief after the operation when I was walking in the hospital and I had one of those pushers you know. And there was no pain. And I kept thinking, I can't believe this, and that's how it's been. I've never had any pain, not at all.
> (Participant 10, female, age 83, 18 weeks post operation)

> There's several times, like when I have got to get up those steps. I put my right foot first and bring my left foot up, and once or twice… you step on your left, and it's still there, lets you know it's still tender. (Participant 12, male, age 78, 16 weeks post operation)

Mental wellbeing

Formatted: Font: Italic

Low mood or depression associated with the reduced mobility due to the fracture was reported by a few interviewees, emphasising the great value placed by interviewees on being independently mobile.

> He can't walk and that, to him he'd rather die. I'll be honest with you he's said it once or twice, "Let me go". And I said, "No you're not going no-where". And then the other day for the first time, but he hasn't said it since, "I'm going to commit suicide", I said, "No you're not, you're not". (Carer of participant 31, male, age 84, 5 week post operation)

> For me it was a massive problem and caused me depression. To me is the most important thing, the mental aspect of taking away somebody's freedom to be able to move around and go to the shops and do all that sort of thing.

For peer review only

(Participant 7, female, age 70, 23 weeks post operation)

Fear of falling

The experience of the fracture left a few participants with a fear of falling and sustaining a further fracture.

> I think it frightened him more than anything else. He's frightened he'll fall over again and do it again, that bothers him more than anything else. Because now when he stands up at all to try and walk he's frightened he's going to fall over and the same thing will happen all over again. (Carer of participant 11, male, age 84, 7 weeks post operation)

> I've got to watch what I'm doing. If I catch my foot on [paving stone], I can go over again. (Participant 12, male, age 78, 16 weeks post operation)

The fear of falling was sometimes expressed by a family member. When talking about his frustration at not being able to work in the garden, participant 6 added

> All the rain has made it very slippery, and [wife] says, "No way do you go out there." (Participant 12, male, age 78, 6 weeks post operation)

This emphasises the value given to stable mobility by interviewees.

Leg shortening

This is a problem that is common following extra-capsular fracture of the proximal femur. One interviewee described her concerns about this.

> One leg is now shorter than the other so that makes walking a bit difficult because it gives me back pain. (Participant 15, female, age 61, 15 weeks post operation)

Variation in how~~**Results**~~

~~Twenty one patients were interviewed on one occasion and 10 were interviewed twice giving a total of 31 individual patients. Of these, 20 (64.5%) were female. Mean age of the patients was 81.5 years (SD 9.2, range 61-96). Of the 31 patients, 12 scored less than eight on the AMTS. Of the 41 interviews, 24 were conducted three to nine weeks, and 17 were conducted 14 to 23 weeks after the hip fracture. Nineteen patient only interviews, 14 carer only, and eight patient/carer dyad interviews were conducted.~~

~~*Active and frail*~~, patients talk about recovery from a hip fracture ~~*very differently*~~

Formatted: Font: Not Italic, Underline

~~Patients~~ Our sample included patients from across a spectrum that extended from those who~~,~~ ~~prior to the fracture,~~ were ~~relatively~~ physically and mentally active ~~were able to provide a detailed description of their recovery, including what was important to them. At the opposite~~

For peer review only

~~end of the spectrum, physically and/or mentally frail patients said almost nothing about their~~ prior to their fracture through to those who, pre-fracture~~. They talked about many health problems but with little clarity about when these health problems occurred. Even with probing, it was difficult to distinguish what experiences were related to their fracture and what to other health problems~~, had been immobile due to conditions such as multiple sclerosis, chronic obstructive airways disease and arthritis ~~or difficulty with breathing. From the perspective of the patient, all their health problems were part of one experience.~~

~~Within the frail group we include~~, and those with severe cognitive impairment~~, many of whom were unable to report on their experience~~. In Box 1 we present condensed versions of the interview summaries for ~~themselves. A few carers were able to give a clear account of mobility and how it had changed since the hip fracture. However, most carers provided more limited accounts from which it was difficult to discern what problems related specifically to the hip fracture. These carers included relatives who provided some care for the patient but did not live with them, and professional carers in nursing or residential homes.~~

~~The~~participants chosen to represent the whole spectrum of patients ~~from active through to frail is illustrated in table 1 where we provide condensed versions of interview summaries~~. We indicate whether the data was provided by patient, carer or both.

Recovery as a return to pre-fracture state or ~~subsumed within the~~as part of aging ~~trajectory~~and decline

Every patient interviewed had experienced a hip fracture and surgery ~~with the associated reduced mobility and pain~~, so in physical terms~~,~~ all ~~the patients~~of them had, for a period of time, been somewhat ~~frailer than~~impaired compared to their pre-fracture ~~status for a period of time. However, the way the participants talked about their recovery varied.~~

state. Four weeks post-operation, those who were active pre-fracture talked in terms of regaining a recovered state that was similar to their pre-fracture state although with some minor adaptations (participants 15 and 20 in ~~table~~box 1~~) although they also~~). Whilst these participants expressed worry about how well they might function in the future~~. However,~~ there was, nevertheless, determination to progress to as full a recovery as possible. Four ~~weeks~~months post-operation many of these participants had all but regained their pre-fracture level of activity. Among participants with severely limited mobility pre-fracture, some were

For peer review only

For peer review only - http://bmjopen.bmj.com/site/about/guidelines.xhtml

able to identify specific activities which were more difficult post-fracture than pre-fracture, such as putting on socks and getting in and out of bed. Some were also able to identify specific improvements in mobility post operation (see participants 9 and 15 in box 1). These participants described a process of recovery although it was very limited.

In contrast, for other participants, the fracture was just one part of a process of aging and decline. For example, participant 11 (see box 1) had been very limited in his activities before the fracture. Post fracture he needed adaptations to his home and increased care support post fracture to enable him to continue to manage at home. The mobility of participant 18 had declined and she had started using a wheelchair instead of her mobility scooter to get out of the house. However, it was unclear whether the decline was due to the concurrent heart failure or the fracture. Those who were frailthe most physically or cognitively impaired pre-fracture did not talk about regaining a recovered state but about a state of no change. They continued with their limited activities as before (for example: participants 23 and 26 in table 1), or box 1). For one participant, the only change only in external circumstances (for example was her move to a new nursing home (participant 5 in table 1) at both interview time points.box 1). Participants with cognitive impairment were often unaware of having experienced a fracture. Some of the frail participants mentioned a specific aspect of daily living that had changed. For example, one participant described being able to walk with one stick instead of two, another had regained the ability to dress herself and another mentioned that the initial post-operative pain had disappeared.

For those between the two extremes of the spectrum it was often difficult to disentangle the impact of the fracture from the impact of other health problems at both interview time points. Participants with severely limited mobility pre-fracture were able to identify specific actions that were difficult post-fracture (for example: participants 3 and 9 in table 1). Other participants tended to see themselves or the person they cared for, as on an aging trajectory with the fracture being just one part of this (for example: participant 11 in table 1). They were often unclear as to what was due to the ageing process or concurrent health problems and what was due to the fracture (for example: participant 18 in tableParticipant 1 box 1).

Recovery through adaptation

In the face of their physical limitations, most participants made adaptations that mitigated against the effect of the fracture; for example employing a cleaner, moving to a residential or

For peer review only

nursing home or using a walking aid or other assistive device. For those who were active pre-fracture, adaptation was mostly considered temporary, although at 4 months there was some evidence that active patients had adapted to some limitations such as being unable to kneel for gardening or ~~finding it exhausting to walk around town.~~limiting time spent shopping to avoid exhaustion. For some participants who had been experiencing decline in their mobility pre-fracture, the fracture precipitated adaptations that ~~made life easier but~~ they had not previously ~~been~~ considered but made their life easier. These included using a wheelchair for shopping, having a new ramp built for getting in and out of the house in a wheelchair, using a walking aid or employing professional carers to assist with personal care. For some, their own or their carer's fear of further falls limited their mobility or at least limited how far they tested their ability to walk. Poor weather conditions exacerbated this fear, but adaptations to the environment such as walking aids or handrails lessened the fear.

Discussion

~~*Changes specific to recovery from hip fractures*~~

~~This section reports on the results of our second analysis approach which involved searching the interviews systematically for data related to recovery from the~~ Following hip fracture~~.~~ ~~The data was coded under the following themes: mobility, activities, self -care, pain, mental wellbeing, fear of falling and leg shortening. Each theme is illustrated with quotations in table 2 where we illustrate the range of experiences among~~ , for those ~~interviewees~~ who ~~were~~ had some pre-fracture mobility and able to articulate what ~~was specifically related to the hip fracture. For each quotation we indicate the age and gender of the patient, time since hip fracture and whether the quotation was from the patient or carer.~~

~~Mobility was the most prominent theme although when talking about~~they value during recovery, stable mobility ~~the interviewees often mentioned other themes (see table 2).~~ , that is, ~~*Personal care* – such as washing, dressing and getting to the toilet – was talked about in interviews, but in many cases it was not clear whether difficulties with personal care were specifically due to the fracture. A few interviewees talked about problems with incontinence but again it was unclear whether this was specific to the fracture. Most patients had a commode or had arranged to sleep near the bathroom in the weeks immediately after the fracture. Activities beyond personal care were talked about by those who were active prior to their fracture. Independence was commonly linked to~~ mobility ~~or ability to undertake activities or ability to self-care.~~ without the ~~*Pain* was described by fewer interviewees than~~

Formatted: Font: Italic

Formatted: Font: Italic

For peer review only

mobility, and some of these interviewees spoke about the lack of pain. *Mental wellbeing* and fear of falling were mentioned by a few interviewees, sometimes linked to mobility or activities that they were avoiding. One interviewee described experiencing shortening of the leg and her concern that she might require a raised shoe and be prone to back pain.

Formatted: Font: Italic

Discussion

The population undergoing surgery for hip fracture includes those who were becoming frail or were already frail before the fracture. These patients have difficulty articulating which aspects of their health experience relate to the fracture and which to other conditions. Those who were active prior to fracture are more able to identify outcomes of or fear of falling, and mobility that that are allows people to undertake valued activities are most valued. The ability to walk is important to them and specific to their fracture. There is no evidence from our study that we can assume that frail and active patients consider the same outcomes as important. Although but so too are other leg movements needed for activities such as gardening or using transport. For some older people see themselves as able to recover from their fracture, many of those who are becoming frail or are already frail see the fracture as part of the aging process. Those able to articulate what was important to them when recovering from their fracture identified mobility, return to activities, self-care, independence, pain and mental wellbeing. For a small number of this group, fear of falling is an issue and one participant in our study was worried about the consequences of leg shortening. Assessment of recovery from hip fracture is complicated by the adaptations patients and carers make; adapting to physical limitationsparticipants, maintaining mobility, however limited, was achieved by using assistive devices or changing activities, recalibration of working out new ways of doing an activity. Some participants adapted to their limitations, for example wearing different footwear or adjusting their expectations of health, protective response of carers includingwhat they could achieve. Others maintained their previous limited function through increased care provision, and changes to the patient's environment. These adaptations affect how patients and their carers respond to questions about outcome from a hip fracture..

Patients also consistently valued certain basic domains of health, such as pain (or lack of it), day-to-day activities, personal care and mental well-being. However, many participants in this study were unable to articulate what was important to them in terms of recovery from hip fracture. The hip fracture was just one part of their decline with age and its impact could not

For peer review only

be disentangled from the impact of other health issues.. The level of recovery perceived by a participant was influenced by their pre-fracture state and their ability to make adaptions during recovery.

Strengths and weaknesses of the study

The study sample was representative of the age profile, gender balance and dementia levels of the UK population experiencing hip fractures (2, 12), with a majority of our sample being older women and the proportion of patients with cognitive impairment being marginally higher than the age matched wider UK population.

When the mortality rate post operation is taken into account, including the higher mortality amongst older females, the study sample was broadly representative of the age profile and gender balance of the population of England, Wales and Northern Ireland experiencing hip fractures (2). We used a higher cut off for assessment of cognitive impairment (score of 8 on AMTS) compared to the NHFD (score of 6 on AMTS). This is likely to explain our higher proportion of participants with cognitive impairment compared to the average in the NHFD.

More research time was spent on recruitment than any other aspect of the study as it proved difficult. When contacted about the interview study, potential participants talked about other priorities or concerns that prevented them agreeing to interview, or they simply did not wish to be interviewed. It is possible that those not interviewed were struggling most with recovery. Our data is also limited by the difficulty some frail older adults have in giving a detailed account of their health experience (19).(18). Interview data is jointly constructed by interviewer and interviewee (20) and our interviewer had no clinical knowledge of hip fractures reducing the interviewer's influence on the data.(19) and our interviewer had no clinical knowledge of hip fractures. This reduced the likelihood of the interviewer influencing the data. A clinician undertaking the interviews would have the knowledge to help the patient tease out whether health problems were fracture related or not. However, this would obscurehave obscured the important finding, that participants often experienced their fracture as part of, rather than separate to, their other existing health problems. For those with cognitive impairment, some carers were unable to give a detailed account of recovery due to limited day-to-day contact with the participant.

Formatted: Font: Italic

Comparison with other studies

For peer review only

There are similarities between our findings and other qualitative studies of similar populations. ~~The spectrum from frail to active patients is similar to that found in a study from a Swedish team that explored engagement with rehabilitation post hip fracture (21). They classified their participants as: those who were frail and in need of support but did not request it; those who were dependent and took no active part in rehabilitation and those who were self-sufficient. Another Swedish study, undertaken with people 12 months after their hip fracture found that mobility and a return to normal activities were key outcomes for patients (22). An Australian study of mobility post-fracture found that reduced level of mobility was associated with fear of falling, physical limitations from other illness and social/environmental factors (23). Our results also echo findings from across the research literature on the experience of health and illness. For example, the difficulty disentangling the impact of one health condition from other co-morbidities has been found for mental health conditions (24). The acceptance of an acute health problem as being part of the aging process has been found for conditions such as stroke~~ A Swedish team that explored engagement with rehabilitation post hip fracture found a similar spectrum of participants (20). They classified their participants as: those who were frail and in need of support but did not request it; those who were dependent and took no active part in rehabilitation and those who were self-sufficient. Another Swedish study, undertaken with people 12 months after their hip fracture found that mobility and a return to normal activities were key outcomes for patients (21). An Australian study of mobility post-fracture found that reduced level of mobility was associated with fear of falling, physical limitations from other illness and social/environmental factors (22). Our results also echo findings from across the research literature on the experience of health and illness. For example, the difficulty disentangling the impact of one health condition from other co-morbidities has been found for mental health conditions (23). The acceptance of an acute health problem as being part of the aging process has been found for conditions such as stroke ~~(25)~~(24)~~. Recalibration to altered circumstances in response to a sudden injury has also been described (26) as have the adaptions, both physical and psychological that people make in order to maintain their quality of life (27). Reduced expectations of health and acceptance of limited function has been described among elderly women (28). Fear of falling is common among older people generally (29).~~. Recalibration to altered circumstances in response to a sudden injury has also been described (25), as have the adaptations- both physical and psychological- that people make in order to maintain their quality of life (26). Reduced expectations of health and acceptance of limited function have been described among elderly women (27). Fear of falling is common among older people

generally (28). The consistency between our findings and other studies suggests that we now have sufficient qualitative evidence to inform policy decisions about the choice of appropriate PROMS for assessing recovery from hip fracture.

Implications for clinicians and policymakers

This study was undertaken in response to a potential policy change involving the use of a PROM to assess patient recovery from hip fracture, the results of which would form part of the evaluation of the quality of care provided for hip fracture. For the population experiencing fragility hip fractures, it is unlikely that a single PROM specific to hip fracture could be developed which is relevant to the whole spectrum of patients from the active through to the frail.. Several of the themes described by our less frailmore active interviewees - mobility, day-to-day activities, self-care, pain and mental wellbeing, are similar to the domains included in currently available generic measures including the EuroQoL EQ-5D (13), the Short Form 36-item Health Survey (SF-36) (30)(29) and the WHOQoL-BREF (31).(30). Both the EQ-5D (3L) and the SF-36 (version 1) have been widely used in trials of people sustaining hip fractures, but for both measures evidence of essential measurement and practical properties is limited (7). However, these themes were most clearly derived from people representing the less frail end of the spectrum. Evaluating the relative benefit of healthcare in patients representing the frailer end of the spectrum, even with generic measures, is more challenging.In the context of a clinical trial where patients are randomised to an intervention and control arm, these measures may be appropriate but they may need to be supplemented by specific tools for selected groups, especially those patients with high-levels of pre-injury function,

Unanswered questions and future research

Our data can provide the basis for the selection of a generic PROM with potential for assessing the quality of care following hip fracture. However, further research would be needed to test its psychometric and practical properties when completed by the diversity of patients and proxies in this population, and to assess its ability to reflect variation in care quality rather than variation in patient and carers adaptability.

In the context of assessing quality of care for a patient population as diverse as those experiencing hip fracture, it may be impossible to devise a single PROM that will be appropriate for all patients. Although quality of care may be one factor that will influence recovery as perceived by a patient, their pre-fracture state, adaptations that they or their carers

For peer review only

make to their reduced mobility, and their perception of whether or not they are at the stage in life where decline is inevitable will all influence how they answer questions contained within a PROM.

For peer review only

Figure 1 Flow chart of study recruitment

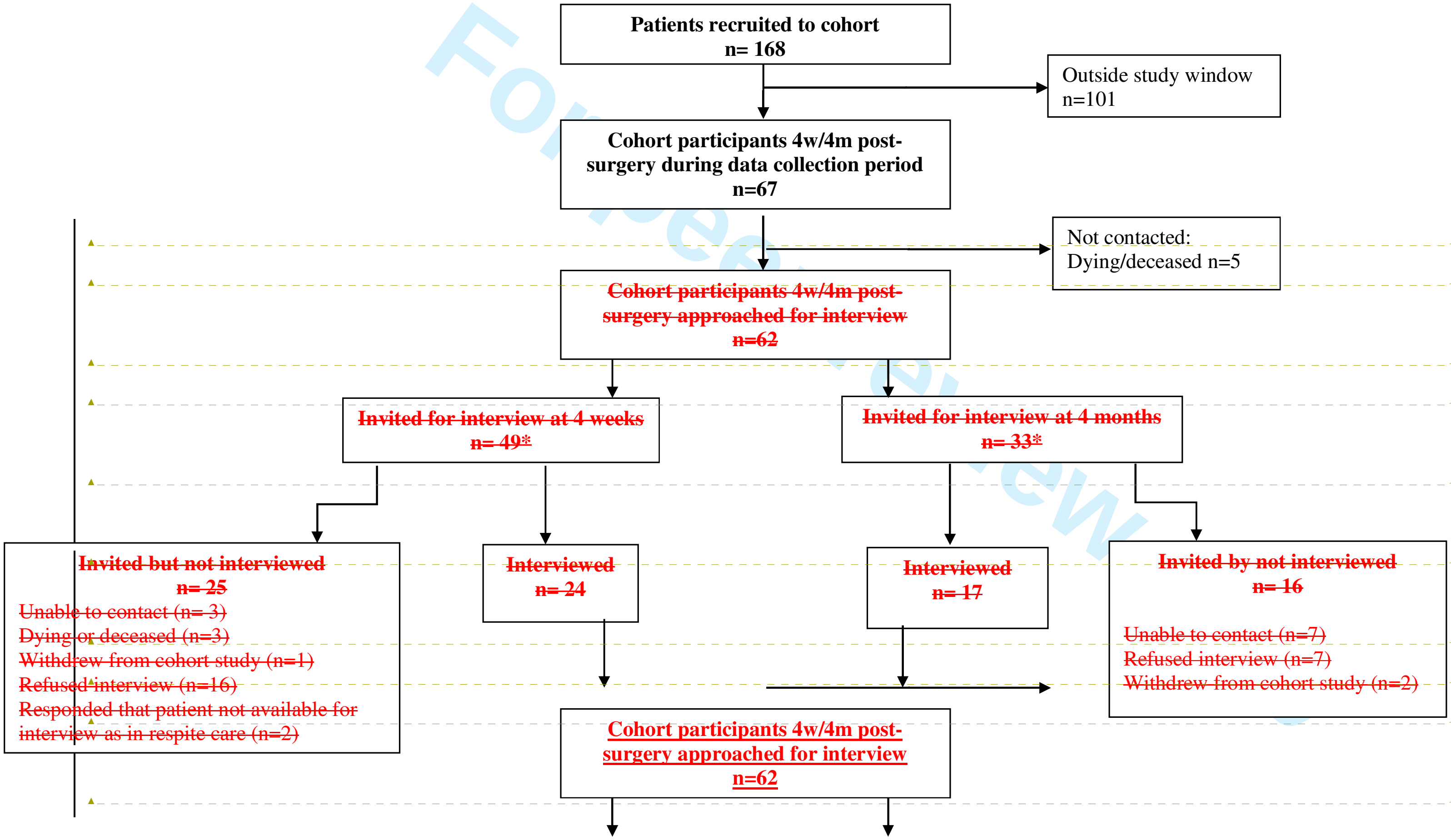

Formatted: Font: Times New Roman, 12 pt

Formatted: Font: Times New Roman, 12 pt

Formatted: Font: Times New Roman, 12 pt

Formatted: Font: Times New Roman, 12 pt

Formatted: Font: Times New Roman, 12 pt

Formatted: Font: Times New Roman, 12 pt

Formatted: Font: Times New Roman, 12 pt

Formatted: Font: Times New Roman, 12 pt

Formatted: Font: Times New Roman, 12 pt

Formatted: Font: Times New Roman, 12 pt

For peer review only

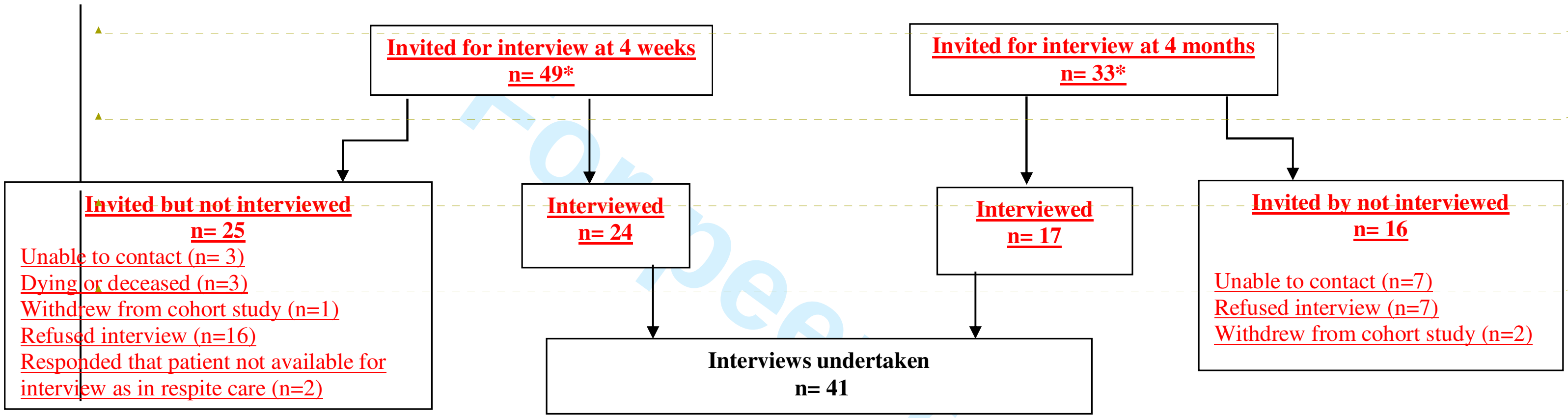

Notes: * 20 participants were invited for interview at both 4 weeks and 4 months post operation

Formatted: Font: Times New Roman, 12 pt

Formatted: Font: Times New Roman, 12 pt

Formatted: Font: Times New Roman, 12 pt

Formatted: Font: Times New Roman, 12 pt

For peer review only

Active people who provided a clear account of fracture, recovery, and intention to get back to being as active as prior to the hip fracture

61 year old female social worker who lives with her husband. Before her fracture she was working full time and, for recreation, taking country walks, undertaking all types of gardening activities and playing with her grandchildren. Post fracture fixation (6 weeks) she described using crutches to get around the garden and shops, needing help with putting on socks and cutting toe nails, and was unable to climb stairs. She had noted shortening of her fractured leg which was causing her concern for the future (back pain, need for built up shoe). She had adapted temporarily to her mobility difficulties by living downstairs in her house, making changes that enabled her to get into the garden, adapted the way she prepared food and paid a cleaner to do housework. She talked in terms of improvement and expectation of returning to work and full activity including cleaning and gardening. During the interview she was able to specifically identify the limitations due to the hip fracture and how these were changing as she recovered. By the second interview she was frustrated that recovery was so slow but she could identify the ways in which she had continued to recover. (Participant 15, interviewed 6 weeks and 15 weeks post operation)

92 year old female who lives alone in her own flat within a sheltered housing complex. Prior to the hip fracture she looked after herself and did her own washing, but had a cleaner to undertake heavy household chores. She spent most of each day out and about at the shops, engaging in social activities, bingo and on outings. She has a daughter who visits at least weekly. She had no other illnesses. Post-fracture fixation she talked about having some initial pain and problems lifting her leg after the operation but was now mobile about her home with a walking frame. The housing complex has a lift which she now used. She was intending to return to getting out and about as she was before her fracture. (Participant 20, interviewed 5 weeks post operation)

Frail people for whom the hip fracture had made almost no difference to their health experience, except where external circumstances changed

88 year old female retired teacher, who lives with her son. Other relatives live nearby. The patient wove together pre and post injury experience in her account, making it difficult to disentangle. She also spoke so softly she was difficult to hear. She said her son does the cooking and cleaning and her daughter assists with self-care. She has a close family, feels well supported and has lots of visitors – friends, grandchildren and great grandchildren. She did not feel her physical health restricted her activities before her fracture, although acknowledged that she depended on her children. Her main interest beyond seeing friends and family is reading. She described being content with life. She mentioned having a diagnosis of multiple sclerosis but its impact on her health was unclear. Prior to her fracture she was unwell with an infection and recounts using a frame for mobility which she still uses. (Participant 23, interviewed 5 weeks post operation)

85 year old female living in a nursing home. Her daughter visits alternate days. Her daughter provided the interview data. The patient has dementia but otherwise had been well before the fracture. Patient gets up and walks about herself, and takes herself to the toilet. She enjoys sitting and chatting. The patient does not remember the injury. Her life has not changed from

For peer review only

~~how it was pre injury. The daughter did not mention any fracture-specific issues related to recovery. (Participant 26, interviewed 6 weeks post operation)~~

~~84 year old female with limited English language. Pre-injury she had carers to assist her with all her personal needs. The injury had occurred whilst being hoisted. Post injury her main concern was that at discharge from hospital, after a three month stay, she was sent to a nursing home where she knew no-one. She mentioned some pain but it was unclear whether this was distinct from back pain which she had experienced for many decades. The interviewer found it difficult to engage the patient on questions about her hip fracture. The patient repeatedly expressed distress about being in the nursing home. During the interview, the nurse, who had known the patient since moving to the nursing home, provided an account of the patient's general function but nothing specific to recovery from the hip fracture. (Participant 5, interviewed 18 weeks post operation)~~

For peer review only

Formatted: Font: Not Bold

Formatted Table

61 year old female social worker who lives with her husband. Before her fracture she was working full time and, for recreation, taking country walks, undertaking all types of gardening activities and playing with her grandchildren. Post fracture fixation (6 weeks) she described using crutches to get around the garden and shops, needing help with putting on socks and cutting toe nails, and was unable to climb stairs. She talked in terms of improvement and expectation of returning to work and full activity including cleaning and gardening. By the second interview she was frustrated that recovery was so slow but she could identify the ways in which she had continued to recover. (Participant 15, interviewed 6 weeks and 15 weeks post operation)

92 year old female who lives alone in her own flat within a sheltered housing complex. Prior to the hip fracture she looked after herself and did her own washing, but had a cleaner to undertake heavy household chores. She spent most of each day out and about at the shops, engaging in social activities, bingo and on outings. She had no other illnesses. Post-fracture fixation she talked about having some initial pain and problems lifting her leg after the operation but was now mobile about her home with a walking frame. The housing complex has a lift which she now used. She was intending to return to getting out and about as she was before her fracture. (Participant 20, interviewed 5 weeks post operation)

92 year old female lives alone with husband. Daughter visits several times a week to help. Poor hearing. Difficult to disentangle what was before and after fracture. Seems to have been able to walk around house, undertake self-care and microwave own meals pre-fracture. Post fixation of the hip fracture, patient slowly improved walking. Life seems very similar to before fracture except need for walking aid~~**People in between the extremes of the spectrum from active through to frail**~~
, inability to put on socks and husband now microwaves the meals. (Participant 9, interviewed 9 weeks post operation)

70 year old male retired painter and decorator who lives with his wife and enjoys almost daily visits from his grandchildren. Mobility restricted to 5-6 metres for more than two years prior to fracture due to knee pain and chronic obstructive pulmonary disease. ~~Known to have osteoporosis pre-fracture.~~ When interviewed he describes struggling to get up the stairs, get in and out of bed, put his shoes and socks on, and bend down. ~~However, it was difficult to disentangle the specific impact of the hip fracture on his activity.~~ Although his mobility was severely restricted prior to his fracture, he described being unable get around as much~~, nor as much~~ as he had done before the fracture. ~~The fracture seems to have been a pivotal event as the patient felt his life had changed.~~ He noted some improvement over recent weeks, as he no longer needed two sticks for walking, only one.
(Participant 3, interviewed 15 weeks post operation)

84 year old male with dementia, who has some lucid moments and some recall of falling and hurting himself. He lives with his wife who looks after him and they have a cleaner to do heavy housework. Wife provided interview, involving the patient in the latter half when he woke up. Patient's walking was gradually slowing and he had a number of falls before his fracture. Fracture occurred while walking in shopping area with his wife. Since fixation of the fracture patient has required assistance with personal care, has professional carers four times a day, and the bathroom has been adapted for his limited mobility. The interviewee had difficulty distinguishing decline due to old age and change due to the fracture. The patient complained of some pain but it was unclear whether this was from the fracture or previously

For peer review only

established osteoarthritis. Before the fracture both patient and wife had ceased all non-essential activities except for a weekly trip to the shops so daily life had changed little except for more care provision. The interviewee commented that the fracture would have had more impact on their lives if the patient had been younger and fitter. (Participant 11, interviewed 7 weeks post operation)

84 year old male who has dementia. He lives alone but received visits three times a day from his son who provides meals. Son was interviewed. Arthritis of knee limited mobility before the fracture. Spent most of the day sitting. At weekends prior to fracture patient went to neighbour's house for evening meal. Patient fell and sustained fracture while walking to neighbour's house. Patient does not recall fracture. At time of interview, the patient was as mobile as pre operation limited by pain and stiffness from arthritis. Not yet visiting neighbour but this was because family was discouraging this in case he falls again rather than due to mobility. (Participant 1, interviewed 16 weeks post operation)

92 year old female lives alone with husband. Daughter visits several times a week to help her parents. Poor hearing. Interview was full of mishearing and jokes between patient and her husband. Difficult to disentangle what was about before and after fracture. Seems to have been able to walk around house, undertake self-care and microwave own meals pre-fracture. Post fixation of the hip fracture, patient slowly improved walking. Life seems very similar to before fracture except need for walking aid and inability to put on socks. Husband now microwaves the meals. (Participant 9, interviewed 9 weeks post operation)

74 year old female who lives with husband. Patient lived with severe rheumatoid arthritis for 30 years. Developed heart failure and admitted to hospital with shortness of breath and confusion. Fell while in hospital and fractured her hip. Mobility before hip fracture very limited – able to walk slowly in house and garden, undertake light chores, and use scooter to go shopping. Became worse with breathing difficulty. Mobility remained reduced after hospital admission. Able to take step slowly in house with support. Uses wheelchair to go out of house – a new ramp improved this by second interview. Unclear how much mobility change was due to the fracture and how much due to heart failure. (Participant 18, interviewed 6 weeks and 18 weeks post operation)

88 year old female retired teacher, who lives with her son and has a diagnosis of multiple sclerosis. The patient wove together pre and post injury experience in her account, making it difficult to disentangle. She said her son does the cooking and cleaning and her daughter assists with self-care. She has a close family, feels well supported and has lots of visitors – friends, grandchildren and great grandchildren. Her main interest beyond seeing friends and family is reading. She described being content with life. Prior to her fracture she was unwell with an infection and recounts using a frame for mobility which she still uses. (Participant 23, interviewed 5 weeks post operation)

85 year old female living in a nursing home. Her daughter visits alternate days. Her daughter provided the interview data. The patient has dementia but otherwise had been well before the fracture. Patient gets up and walks about herself, and takes herself to the toilet. She enjoys sitting and chatting. The patient does not remember the injury. Her life has not changed from how it was pre injury. The daughter did not mention any fracture-specific issues related to recovery. (Participant 26, interviewed 6 weeks post operation)

84 year old female with limited English language. Pre-injury she had carers to assist her with

For peer review only

all her personal needs. The injury had occurred whilst being hoisted. Post injury her main concern was that at discharge from hospital, after a three month stay, she was sent to a nursing home where she knew no-one. The patient repeatedly expressed distress about being in the nursing home but did not talk about the fracture.
(Participant 5, interviewed 18 weeks post operation)

84 year old male who has dementia. He lives alone but received visits three times a day from his son who provides meals. Son was interviewed. Arthritis of knee limited mobility before the fracture. Spent most of the day sitting. At weekends prior to fracture patient went to neighbour's house for evening meal. Patient fell and sustained fracture while walking to neighbour's house. Patient does not recall fracture. At time of interview, the patient was as mobile as pre operation limited by pain and stiffness from arthritis. Not yet visiting neighbour but this was because family was discouraging this in case he falls again rather than due to mobility. (Participant 1, interviewed 16 weeks post operation)

~~Table~~Box 1 Summaries of the data about individual patients ~~illustrating the spectrum of patients from active through to frail~~ and their recovery from a hip fracture

Formatted: Font: Not Bold, Font color: Black

For peer review only

Theme	Examples from interviews
Mobility<br>Walking and other manoeuvres with the legs.	I'm walking with a walking stick at the moment. I've been down the park and back…I can usually get around [the house] without the walking stick, and I can get up and down stairs no problem. I get upstairs with my good leg and downstairs with my bad leg.<br>(Participant 6, male, age 78, 5 weeks post operation)<br><br>I can walk with crutches, but some days it's a lot slower than other days.<br>(Participant 7, female, age 70, 5 weeks post operation)<br><br>Her mobility's getting better. I think she'll cope with the frame. She's had a couple of falls in the home, earlier when she was forgetting that she had to use the frame. She'd get out of bed and not use the frame and consequently fall. But she's got in the habit of using it now… she's not falling, which is a bonus.<br>(Carer of participant 13, female, age 87, 14 weeks post operation)<br><br>I went to see the specialist and I told him I can't put my full weight on it. I walk with a stick now.<br>(Participant 3, male, age 70, 15 weeks post operation)<br><br>The operation was successful and got him back to normal right from the start, right from the very first day that he had it done. He was able to then walk pain free with a Zimmer frame to the toilet. The staff were all saying it was amazing how well he was walking and he would soon be back to normal, but what they didn't realise was that he was walking normally and it looked slightly less good than normal because of his knees. So I think he was as right as rain right from the start. I think the operation was a fantastic success.<br>(Carer of participant 1, male, age 84, 16 weeks post operation)<br><br>I have used my stick quite a bit although I have walked a long way while I have been [on holiday]. I try sometimes to walk without the stick but I do it with a bit of a limp.<br>(Participant 6, male, age 78, 16 weeks post operation)<br><br>When I was in hospital I couldn't get out of bed… but now when I've been in bed all night I can push myself over …I can pick up my leg and just swing it out of bed… to me, [that] is a bonus, a big bonus.<br>(Participant 9, female, age 92, 16 weeks post operation)<br><br>When I get out of the car I have to release my seat and take it back, put my right foot out first, then get hold of the bottom of my trouser leg, lift that up and lift my leg out. It is a bit awkward lifting my leg up.<br>(Participant 12, male, age 78, 16 weeks post operation)<br><br>Well it is a bit different because I'm not… I can't rush round like I did, but eventually that will come…I mean it's pretty normal now, but I think it's going to be a while before I can actually walk as I did and I probably won't walk as I did… when I came home [from hospital] I was still hobbling... but now I'm more or less…walking normal, especially with the stick…I couldn't climb a step ladder or anything like that,

For peer review only

	well I'd be a bit nervous anyway, whereas before if I wanted to decorate a room I would go ahead and do it. …I would go for long walks and that's something I cannot do.<br>(Participant 10, female, age 83, 18 weeks post operation)
**Activities**<br>Activities beyond personal care.	I just miss getting up and getting out. I never stayed in. I'd go out in the morning and come back and then I'd go out again, I just used to go out looking round the shops. I just get these crossword books and I do those.<br>(Participant 20, female, age 92, 5 weeks post operation)<br><br>I'm back on what I call domestic duties – washing up! But the thing that is frustrating is that I can't get outside and do any gardening. We've got a hawthorn hedge, and for me to do it, it'd take me about an hour. And of course, I can just go along and do it. All the rain has made it very slippery, and [wife] says, "No way do you go out there."<br>(Participant 12, male, age 78, 6 weeks post operation)<br><br>Everything is affected really. I haven't been able to go out in the garden to do any gardening, even though we had nice weather, because I can't get down on my knees. I haven't been able to wash the windows. I couldn't wash my own car, my husband did that, but normally I would have done it myself. The only thing I'm still able to do that I used to do is read. I've read a lot and I've got a Kindle so I've been able to download books, so I haven't been relying on having to go out and buy books or borrow books. The other thing that remains the same is, I can watch television.<br>(Participant 15, female, age 61, 6 weeks post operation)<br><br>Whenever we fancied the day out, we would drive to [list of local UK towns]. She would hire a scooter and go into town and just have a nice day. Go and have lunch. But this has put a top hat on that up to now anyway. Because at the moment she can't get on a scooter.<br>(Husband of participant 18, female, age 74, 6 weeks post operation)<br><br>I can do little jobs but because I haven't got as much movement in the hip joints, I find it difficult to go down on my hands and knees. With gardening you need to get down on your hands and knees for planting and things, and that is not possible. If I go down on one knee it's difficult to get up again so that's not possible but I can do things that are higher up, I can trim.<br>(Participant 15, female, age 61, 15 weeks post operation)<br><br>Over the last three weeks, when we go out shopping now, I can't go down the aisles, so [daughter] gets me a chair and I can sit in the chair and then say what shopping I need, that is very good.<br>(Participant 9, female, age 92, 18 weeks post operation)<br><br>I'm tackling a little bit of cooking now. I started to cook myself some nice lunches and I haven't got round to the… scones … I made one lot when I came home and I thought, I can't be bothered anymore.<br>(Participant 10, female, age 83, 18 weeks post operation)
**Self-care**<br>Self-care (washing, dressing) limited	I can wash and dress myself, at least, up to a point I can. I mean I can't stand up for very long so I mean I have to have a wash sitting down. It is a bit difficult to try and get dressed and undressed, at the moment anyway, because I can't stand for long.<br>(Participant 28, female, age 89, 4 weeks post operation)

For peer review only - http://bmjopen.bmj.com/site/about/guidelines.xhtml

due to fracture.	I'm also worried about how much movement I will have in my hip, because I'm still not able to put a sock or anything on my injured leg. I can manage now with my trouser leg and throw these jogging trousers and hook my leg into them but I have to ask my husband if I need to put a sock or a shoe, or my slipper on that foot. I have to ask him.<br>(Participant 15, female, age 61, 6 weeks post operation)<br><br>Something that he [husband] would never do that he did. Last night I was struggling in there [bathroom] to get my clothes off and he opened the door and says, "Come on, come on, let's have your feet up". He lifted my feet up, took my socks off, because I was struggling. So he did and I thought he wouldn't have done that.<br>(Participant 9, female, age 92, 9 weeks post operation)<br><br>I still have to throw my clothes and hook them onto the foot to get dressed. I couldn't wear lace-up shoes or anything like that because I couldn't tie them up, but things like slip-ons and sandals I can get on quite easily, so I'm fairly independent – I am independent really, I just need help with cutting my toenails and that – those on the right foot that's all.<br>(Participant 15, female, age 61, 15 weeks post operation)
**Pain**<br>Pain specifically from the hip fracture.	So here I am, four or five weeks [post operation], I get a little bit of pain, not a lot.<br>(Participant 7, female, age 70, 5 weeks post operation)<br><br>When anybody asked if he was in pain he said it was his hip on both sides and his knee on both sides and the leg break was on the thigh and he said that was fine. If they asked him how he was he'd say oh that's fine no problem and I don't think he ever felt pain from that once the operation had been done.<br>(Carer of participant 1, male, age 84, 16 weeks post operation)<br><br>There's several times, like when I have got to get up those steps. I put my right foot first and bring my left foot up, and once or twice… you step on your left, and it's still there, lets you know it's still tender.<br>(Participant 12, male, age 78, 16 weeks post operation)<br><br>The pain was so bad before I had it done, and I just couldn't believe the relief after the operation when I was walking in the hospital and I had one of those pushers you know. And there was no pain. And I kept thinking, I can't believe this, and that's how it's been. I've never had any pain, not at all.<br>(Participant 10, female, age 83, 18 weeks post operation)<br><br>Not at all, not at all. I don't get any pain at all or… I'm walking quite normal now.<br>(Participant 7, female, age 70, 23 weeks post operation)
**Mental wellbeing**<br>Depression or low mood specifically attributed to the experience of	He can't walk and that, to him he'd rather die. I'll be honest with you he's said it once or twice, "Let me go". And I said, "No you're not going nowhere". And then the other day for the first time, but he hasn't said it since, "I'm going to commit suicide", I said, "No you're not, you're not". I said, "You'll get over this [patient's name], you'll beat it". And he will.<br>(Carer of participant 31, male, age 84, 5 week post operation)

fragility fracture and recovery.	Lowness of mood does come on sometimes and I think, oh god, you know, why did that happen<br>(Participant 10, female, age 83, 18 weeks post operation)<br><br>It is not just in terms of the physical aspect but in terms of the mental, that to me is much more important, especially to somebody who is active. If they're not such an active person perhaps they don't mind sitting in a chair all day and all night, some people might not find that a problem. For me it was a massive problem and caused me depression. To me is the most important thing, the mental aspect of taking away somebody's freedom to be able to move around and go to the shops and do all that sort of thing.<br>(Participant 7, female, age 70, 23 weeks post operation)
**Fear of falling**<br>Fear of a further fall expressed by patient or carer.	I think it frightened him more than anything else. He's frightened he'll fall over again and do it again, that bothers him more than anything else. Because now when he stands up at all to try and walk he's frightened he's going to fall over and the same thing will happen all over again. But I think apart from that, if he gets to the state where he can walk about by himself, alright with his stick or you know a part of his chair and walk about, he's going to be quite happy. It's going to make his life a lot better. It's just that initial, I think getting over this fear of falling and having the same thing happen all over again.<br>(Carer of participant 11, male, age 84, 7 weeks post operation)<br><br>The only thing sometimes you get worried about is falling over, it's strange falling over, you wouldn't believe it really. At one time you would fall over and pick yourself up and dust yourself off and carry on.<br>(Participant 6, male, age 78, 16 weeks post operation)<br><br>I've got to watch what I'm doing. If I catch my foot on [paving stone], I can go over again.<br>(Participant 12, male, age 78, 16 weeks post operation)
**Leg shortening**<br>A problem well known clinically following extra-capsular fracture of the proximal femur.	One leg is now shorter than the other so that makes walking a bit difficult because it gives me back pain and it gives me pain funnily enough at the bottom part of my leg, not at the top, between the knee and the ankle that's very p but I am able to walk.<br>(Participant 15, female, age 61, 15 weeks post operation)

Table 2 Themes and illustrative quotations from interviews with patients able to articulate what is important to them during recovery post hip fracture

Formatted: Space After: 10 pt, Line spacing: Multiple 1.15 li

Formatted: Font: Not Bold, Font color: Black

For peer review only

References

1. Johnell O, Kanis JA. An estimate of the worldwide prevalence, mortality and disability associated with hip fracture. . Osteoporosis international: a journal established as result of cooperation between the European Foundation for Osteoporosis and the National Osteoporosis Foundation of the USA. 2004;15:897-902.

2. ~~Executive NHFD.~~Johansen A, Wakeman R, Boulton C, Plant F, Roberts J, Williams A. National Hip Fracture Database~~. [30/11/2012]; Available from: www.nhfd.co.uk.~~ National report 2013. Clinical Effectiveness and Evaluation Unit at the Royal College of Physicians, 2013.

3. Department of Health. Update to Best Practice Tariffs~~.~~; Department of Health; 2011 [5th November 2012~~]~~;]. Available from: http://www.dh.gov.uk/health/2011/12/bpt-update/.

4. Devlin N, Appleby J. Getting the Most Out of PROMs. Putting health outcomes at the heart of NHS decision-making. London: The King's Fund, 2010.

5. Patrick DL, Burke LB, Powers JH, Scott JA, Rock EP, Dawisha S, et al. Patient-reported outcomes to support medical product labeling claims: FDA perspective. Value Health. 2007;10:S125-S37.

6. Fitzpatrick R. Patient-reported outcome measures and performance measurement. In: Smith P, Mossialos E, Papnicolas I, Leatherman S, editors. Performance measurement for health system improvement: experiences, challenges and prospects. Cambridge: Cambridge University Press; 2009.

7. Haywood K, Brett J, Tutton E, Staniszewska S, editors. How well are we measuring patient-centred outcomes following traumatic hip fracture? A systematic review of patient-reported outcome measures (PROMs). Royal College of Nursing International Research Conference; 2012 23-25th April 2012; London: Royal College of Nursing.

8. Griffin X, Achten J, Parsons N, Boardman F, Griffiths F, Costa M. The Warwick Hip Trauma Evaluation - an abridged protocol for the WHiTE Study. A multiple embedded randomised controlled trial cohort study. . Bone Joint Res. 2012;1(11):310-4.

9. Hodkinson HM. Evaluation of a mental test score for the assessment of mental impairment in the elderly. Age and Ageing. 1972;1(4):233-8.

10. MacKenzie DM, Copp P, Shaw RJ, Goodwin GM. Brief cognitive screening of the elderly: a comparison of the Mini-Mental State Examination (MMSE), Abbreviated Mental Test (AMT) and Mental Status Questionnaire (MSQ). Psychol Med. 1996;26(2):427-30. ~~Epub 1996/03/01.~~

11. Mental Capacity Act 2005, ~~Her Majesty's Stationary Office; (~~2005~~.)~~.

12. Currie C, Fleming S, Partridge M, Plant F, Wakeman R, Williams A. National Hip Fracture Database National Report. 2010.

13. The EuroQoL Group. EuroQoL: a new facility for the measurement of health-related quality of life. Health Policy. 1990;16(3):199-208.

14. McConnell-Henry T, Chapman Y, Francis K. Husserl and Heidegger: Exploring the disparity. International Journal of Nursing Practice. 2009;15(1):7-15.

15. Jacelon C. Older adults' participation in research. Nurse Researcher. 2007;14(4):64-73.

~~15~~16. NVivo qualitative data analysis software. 10 ed. Melbourne, Australia: QSR International Pty Ltd; 2012.

~~16~~17. Borkan J. In: Crabtree BF, Miller WL, editors. Doing Qualitative Research. Second ed. London: Sage Publications; 1999.

~~17.~~ 18~~McConnell-Henry T, Chapman Y, Francis K. Husserl and Heidegger: Exploring the disparity. International Journal of Nursing Practice. 2009;15(1):7-15.~~

~~18. Miller A. Realism. In: Zalta EN, editor. The Stanford Encyclopedia of Philosophy: Stanford University; 2012.~~

Formatted: Font: Calibri, 11 pt
Formatted: EndNote Bibliography
Formatted: Font: Calibri, 11 pt
Formatted: Font: Calibri, 11 pt
Formatted: Font: Calibri, 11 pt
Formatted: Font: Calibri, 11 pt
Formatted: Font: Calibri, 11 pt
Formatted: Font: Calibri, 11 pt
Formatted: Font: Calibri, 11 pt
Formatted: Font: Calibri, 11 pt
Formatted: Font: Calibri, 11 pt
Formatted: Font: Calibri, 11 pt
Formatted: Font: Calibri, 11 pt
Formatted: Font: Calibri, 11 pt
Formatted: Font: Calibri, 11 pt
Formatted: Font: Calibri, 11 pt

For peer review only

~~1~~9. Kvigne K, Gjengedal E, Kirkevold M. Gaining access to the life-world of women suffering from stroke: methodological issues in empirical phenomenological studies. Journal of Advanced Nursing. 2002;40(1):61-8.

~~20~~19. Mishler E. Research interviewing: Context and narrative. Cambridge, MA: Harvard University Press; 1986.

~~21~~20. Olsson LE, Nystrom AE, Karlsson J, Ekman I. Admitted with a hip fracture: patient perceptions of rehabilitation. J Clin Nurs. 2007;16(5):853-9. ~~Epub 2007/04/28.~~

~~22~~21. Ziden L, Scherman MH, Wenestam CG. The break remains - elderly people's experiences of a hip fracture 1 year after discharge. Disabil Rehabil. 2010;32(2):103-13. ~~Epub 2009/06/30.~~

~~23~~22. Taylor NF, Barelli C, Harding KE. Community ambulation before and after hip fracture: a qualitative analysis. Disabil Rehabil. 2010;32(15):1281-90. ~~Epub 2010/02/17.~~

~~24~~23. Connell J, Brazier J, O'Cathain A, Lloyd-Jones M, Paisley S. Quality of life of people with mental health problems: a synthesis of qualitative research. Health and Quality of Life Outcomes. 2012;10:138.

~~25~~24. Faircloth CA, Boylstein C, Rittman M, Young ME, Gubrium J. Sudden illness and biographical flow in narratives of stroke recovery. Sociology of Health and Illness. 2004;26(2):242-61.

~~26~~25. Bury M. Chronic illness as biographical disruption. Sociology of Health and Illness. 1982;4(2):169-82.

~~27~~26. Clarke P, Black S. Quality of Life Following Stroke: Negotiating Disability, Identity, and Resources. Journal of Applied Gerontology. 2005;24:319-36.

~~28~~27. Chambers P. Getting Beyond 'Mustn't Grumble' and 'What Can I Expect At My Age': A Narrative Approach to Older Women's Health Stories. In: Narrative, Memory and Health. Huddersfield: University of Huddersfield; 2003. p. 127-39.

~~29~~28. Scheffer AC, Schuurmans MJ, van Dijk N, van der Hooft T, de Rooij SE. Fear of falling: measurement strategy, prevalence, risk factors and consequences among older persons. Age and Ageing. 2008;37(1):19-24.

~~30~~29. Ware Jr. J, Gandek B. Overview of the SF-36 Health Survey and the International Quality of Life Assessment (IQOLA) Project. Journal of clinical epidemiology. 1998;51(11):1203-14.

~~31~~30. Molzahn A, Kalfoss M, Schick Makaroff K, Skevington S. Comparing the importance of different aspects of quality of life to older adults across diverse cultures. Age Ageing. 2011;40(2):192-9.

Formatted: EndNote Bibliography

Formatted: Font: Calibri, 11 pt

Formatted: Font: Calibri, 11 pt

Formatted: Font: Calibri, 11 pt

Formatted: Font: Calibri, 11 pt

Formatted: Font: Calibri, 11 pt

Formatted: Font: Calibri, 11 pt

Formatted: Font: Calibri, 11 pt

Formatted: Font: Calibri, 11 pt

Formatted: Font: Calibri, 11 pt

Formatted: Font: Calibri, 11 pt

Formatted: Font: Calibri, 11 pt

Formatted: Font: Calibri, 11 pt

Formatted: Font: Calibri, 11 pt

Formatted: Font: Calibri, 11 pt

Formatted: Font: Calibri, 11 pt

Formatted: Font: Calibri, 11 pt

For peer review only

Evaluating recovery following hip fracture: a qualitative interview study of what is important to patients

Journal:	*BMJ Open*
Manuscript ID:	bmjopen-2014-005406.R1
Article Type:	Research
Date Submitted by the Author:	08-Jul-2014
Complete List of Authors:	Griffiths, Frances; University of Warwick, Warwick Medical School<br>Mason, Victoria; University of Warwick, Warwick Medical School<br>Boardman, Felicity; University of Warwick, Warwick Medical School<br>Dennick, Katherine; Kings College, London, Florence Nightingale School of Nursing and Midwifery<br>Haywood, Kirstie; University of Warwick, Warwick Medical School<br>Achten, Juul; University of Warwick, Warwick Medical School<br>Parsons, Nicholas; University of Warwick, Warwick Medical School<br>Griffin, Xavier; University of Warwick, Warwick Medical School<br>Costa, Matthew; University of Warwick, Warwick Medical School
**Primary Subject Heading**:	Health services research
Secondary Subject Heading:	Qualitative research, Surgery
Keywords:	Hip < ORTHOPAEDIC & TRAUMA SURGERY, Quality in health care < HEALTH SERVICES ADMINISTRATION & MANAGEMENT, QUALITATIVE RESEARCH

SCHOLARONE™ Manuscripts

Evaluating recovery following hip fracture: a qualitative interview study of what is important to patients

Frances Griffiths, Professor of Medicine in Society, Warwick Medical School, University of Warwick, Coventry, CV4 7AL, UK

Victoria Mason, Research Fellow, Warwick Medical School, University of Warwick, Coventry, CV4 7AL, UK

Felicity Boardman, Mildred Blaxter Post-Doctoral Fellow, Warwick Medical School, University of Warwick, Coventry, CV4 7AL, UK

Kathyrn Dennick, Research Associate, Florence Nightingale School of Nursing and Midwifery, King's College London, SE1 8WA

Kirstie Haywood, Senior Research Fellow, Royal College of Nursing Research Institute, University of Warwick, CV4 7AL, UK

Juul Achten, Research Manager Warwick Orthopaedics, Warwick Medical School, University of Warwick, Coventry, CV4 7AL, UK

Nick Parsons, Senior Research Fellow, Statistics and Epidemiology, Warwick Medical School, University of Warwick, Coventry, CV4 7AL, UK

Xavier Griffin, Specialist Trainee Trauma and Orthopaedic Surgery, Warwick Medical School, University of Warwick, Clinical Sciences Building, Clifford Bridge Road, Coventry, CV2 2DX, UK

Matt Costa, Professor Trauma and Orthopaedics, Warwick Clinical Trials Unit, Warwick Medical School, University of Warwick, Coventry, CV4 7AL, UK

Correspondence to: F.E.Griffiths@warwick.ac.uk

For peer review only

Abstract

Objective

To explore what patients consider important when evaluating their recovery from hip fracture and to consider how these priorities could be used in the evaluation of the quality of hip fracture services.

Design

Semi-structured interviews exploring the experience of recovery from hip fracture at two time points - four weeks and four months post-operative hip fixation. Two approaches to analysis: thematic analysis of data specifically related to recovery from hip fracture; summarising the participant's experience overall.

Participants

31 participants recruited, of whom 20 were female and 12 were cognitively impaired. Mean age 81.5 years. Interviews provided by 19 patients, 14 carers, and 8 patient/carer dyad; 10 participants were interviewed twice.

Setting

Single major trauma centre in the West Midlands of the UK.

Results

Stable mobility (without falls or fear of falls), for valued activities was considered most important by participants who had some pre-fracture mobility and were able to articulate what they valued during recovery. Mobility was important for managing personal care, for day-to-day activities such as shopping and gardening, and maintenance of mental well-being. Some participants used assistive mobility devices or adapted to their limitations. Others maintained their previous limited function through increased care provision. Many participants were unable to articulate what they valued as hip fracture was perceived as part of their decline with age. The fracture and problems from other health conditions were an inseparable part of one health experience.

Conclusions

Patients consistently valued stable mobility and its role in other basic health domains. For evaluating service quality, no one patient-reported outcome measure (PROM) could consistently evaluate recovery for patients with hip fracture. General health-related quality of life tools may provide useful information within clinical trials but may need to be supplemented by specific tools for selected groups, especially those patients with high-levels of pre-injury function.

For peer review only

Key words: Hip fractures, Outcome assessment (Health Care), Interview, Frail older adults

Article summary

Article focus

- The UK NHS has identified the need to evaluate service provision for patients with a hip fracture
- There is increasing expectation that patient-reported outcome measures (PROM) are used within health service evaluation
- We asked the question: what do patients who have recently experienced a hip fracture consider important when evaluating their recovery?

Key messages

- Patients active before their fracture value mobility without falls or fear of falls, to undertake valued activities but many patients consider fracture to be part of their decline with age.
- While no one PROM could evaluate all aspects of recovery for patients with hip fracture, general health-related quality of life tools may provide useful information for the majority of patients.

Strengths and limitations

- The study sample was representative of the age profile, gender balance and dementia levels of NHS patients experiencing hip fractures
- It is possible that those not agreeing to be interviewed were struggling most with recovery.
- The data is limited by the difficulty the more physically and cognitively impaired patients had in giving a detailed account of their health experience.

For peer review only

Introduction

Fragility fracture of the proximal femur (hip fracture) is one of the greatest challenges facing the healthcare community. In 1990, a global incidence of 1.31 million was reported and was associated with 740,000 deaths (1). Hip fractures constitute a heavy socioeconomic burden worldwide. The cost of this clinical problem is estimated at 1.75 million disability adjusted life years lost; 1.4% of the total healthcare burden in established market economies (1). Among those experiencing fragility hip fracture in England, Wales and Northern Ireland, 70% are aged 80 years or older, 73% are female and 34% are cognitively impaired pre-operation. The mortality rate within 30 days of operation was 8.2% in 2013 (2).

The NHS has identified the need to evaluate the quality of service provision for patients with a hip fracture; this evaluation is conducted through the National Hip Fracture Audit Database (NHFD)(2). Currently, aspects of care such as time to surgery, length of patient stay and patient mortality in hospital and 30 day and 120 day follow up are recorded in the NHFD. These data are now used to guide payments to healthcare providers; the payment being increased if the provider supplies 'best practice' care (3). However, while important, there is interest from policy makers in the potential to enhance these currently reported data fields by including and an assessment of outcome as reported by patients. It is increasingly expected that healthcare evaluations should include domains of health that are important to patients (4), captured by well-developed patient-reported outcome measures (PROMs) which aim to assess how patients function and feel in relation to a health condition or associated treatment (5). PROMS capture information that cannot be obtained by other means (5, 6) complementing more traditional performance or process-based measures.

Our aim was to establish whether or not one PROM could be used with all patients who experience a fragility hip fracture as part of the evaluation of the quality of health care for hip fracture delivered by the NHS. For this patient group we were unable to identify a PROM specific to the assessment of hip fracture, and robust evidence of the quality and acceptability of non-hip fracture specific PROMs following completion by patients sustaining a hip fracture is limited (7). Moreover, clarity with regards to the outcomes of healthcare that these patients considers relevant and important does not exist. Appropriate and relevant PROM-based assessment should be underpinned by an understanding of what is important to patients in terms of the outcomes of healthcare. We therefore designed an interview study to explore

For peer review only

with patients and, where appropriate, their carers, what they consider to be important outcomes and to explore variation across this patient group. Our research questions were:

1. What do patients who have recently experienced a hip fracture consider important when evaluating their recovery?
2. Is there variation within this population of the experience of what is considered important in recovery from hip fracture?

These research questions are framed by the desire of policy makers to evaluate the quality of care for hip fracture through assessment of recovery from the perspective of the patient.

Method

Study Design

We conducted semi-structured interviews with patients and, where appropriate, their carers at two time points, at approximately four weeks and then again at four months after they had sustained a fragility hip fracture.

Identification of patients with a hip fracture

We recruited participants from an existing cohort study, the Warwick Hip Trauma Evaluation (8), that commenced January 2012. This is a cohort of all patients admitted with a hip fracture to a single major trauma centre in the West Midlands of the United Kingdom. As part of their pre-operative assessment, patients were assessed for their capacity to consent using clinical assessment and the Abbreviated Mental Test Score (AMTS) (9). The AMTS is a 10-item measure used to rapidly assess the possibility of cognitive impairment in elderly people. A score below 8 suggests cognitive impairment (10). Scores less than 8 were taken to indicate that a patient was unlikely to be able to consent for themselves. Those deemed to have capacity for consenting to surgery, based on clinical assessment and AMTS, were considered able to consent for this study. Following the emergency surgery for their fracture, those with capacity gave written consent to be approached for interview. For those deemed not to have capacity due to cognitive impairment, verbal consent was obtained from their consultee (11). Ethical approval was granted by NHS REC London - Camberwell and St Giles (11/LO/0927) on the 18th August 2011.

Sampling

For peer review only

During the data collection period for this study, February to August 2012, we purposefully sampled cohort participants who had reached 4 weeks or 4 months following their hip fracture and had consented to be approached for interview. The time points were chosen to be the same as those used for data collection for the NHFD (12). If a PROM were to be used with this patient population to assess quality of care, patients would be asked to complete the PROM at these time points. Our sampling strategy ensured a diverse mix of patients with respect to the following factors: age, gender, AMTS (9) and EQ-5D score (13).

Interview recruitment and consent process

We contacted eligible patients and carers by telephone just prior to 4 weeks and/or 4 months following hip fracture first to invite them to be interviewed, then to arrange an interview. If patients declined to participate, the reasons offered were recorded. Patients with capacity to consent were contacted directly. For those patients deemed not to have capacity, we contacted their consultee. Patients able to consent for themselves signed their own consent forms. For those unable to consent the consultee signed an agreement form and we aimed to interview a carer as well as the patient (patient/carer dyad). Carers who were interviewed signed a consent form. Recruitment continued to data saturation at the first time point. The study flow diagram is at Figure 1.

Interview process

We interviewed participants at their current residence (own home, residential or nursing home) or in hospital. The interviewer was trained in interviewing but did not have clinical knowledge of hip fracture, its treatment or prognosis. Where possible, patients and carers were interviewed alone, however where the carer and patient requested a joint interview (whether or not the patient had cognitive impairment), they were interviewed together. The aim of the interviews was to understand each participant's lived experience of hip fracture (14) and the influence of their social context and pre-fracture health. We use the following questions:

- What is a normal day like for you now?
- How bothersome are you finding your hip?
- What is different about your life now compared to just before your injury?
- Compared to just before your injury what has stayed the same?
- Which of these make the most difference to your life?

The interviewer encouraged participants to talk about the experience in whatever order they chose and using terms meaningful to them. Later in the interview we prompted, where necessary, for clarification about what in the patient experience was related to the hip

For peer review only

fracture. Towards the end of the interview we directly asked what was important to them in terms of recovery if this had not already been talked about by the participant, using the following questions:

- What is important to you in terms of your recovery?
- Where would you like to see yourself in the future in relation to your recovery (i.e. the next few weeks and months)?
- If a friend or neighbour were asking you now about how well you are recovering – what has been important to you that you would tell them about?
- If a doctor or nurse was asking you now about how well you are recovering – what would be important for the doctor or nurse to ask about?

Consideration was given to the potential challenges associated with interviewing older adults, for example by giving potential participants sufficient time to decide whether or not to participate and minimising burden and fatigue through streamlining questions (15). The interview process, questions and prompts were refined by the study team during the initial stage of data collection. Questions were similar for both patient and carer. Interviews were audio recorded and transcribed verbatim. For one interview, audio recording was not feasible due to the noisy environment so extensive field notes were taken and transcribed. For all interviews the researcher made field notes to assist interpretation of the interview data.

Analysis

Interview transcripts were checked, anonymised and uploaded into Nvivo software (16). Initial analysis involved data immersion, reading and re-reading each transcript and discussion of the interview transcripts by the research team. All team members read at least five transcripts so all transcripts were read by at least two team members. From the data we identified and crystallised what was important for participants that was specific to hip fracture recovery (17). We found that the interviews at four weeks and four months covered very similar issues, although, as would be expected, what the participants reported about each issue four weeks and at four months was different, as recovery was more advanced at four months. As our analysis aimed to identify what patients consider important when evaluating their recovery rather than the detail of recovery itself, we treated all the interviews related to one participant as one set of data. During data interpretation we took account of the timing of the interview, whether the interview data was from a patient or carer or patient/carer dyad, and field notes. Two different approaches to analysis were then undertaken in response to our research questions.

For peer review only

To answer our first research question, we searched the transcripts for any mention by the participants of what was important to them during recovery from hip fracture. These were discussed at team analysis meetings. Transcripts were then coded in NVivo. As coding proceeded, we reviewed these codes at our team analysis meetings and combined them into themes. After we had read, discussed and then coded ten transcripts we found no additional themes in the remaining data. Double coding was undertaken for one in four transcripts and coding compared and discussed to check consistency of final coding. During analysis we became aware that although the data from different participants could be coded under the same theme such as mobility, there was variation in the experience of recovery. This led us to our second research question and analysis approach.

To answer our second research question, from close reading of the first five interview transcripts we developed, from the data, a template for summarising the experience of hip fracture recovery for each patient carer dyad. This involved considering each set of interviews as a whole, reading and rereading the text and writing a summary of the patient/carer journey and all that influenced it. We reviewed the summaries at our data analysis meetings and from these initial summaries we developed a draft template. We refined the template as we summarised and discussed further transcripts. The template included: current and recent past living arrangements and environment, day-to-day life now and in the recent past, the impact of the hip fracture and its management, what was changing in day-to-day life as they recovered, the extent to which the patient referred specifically to the fracture and their ability to engage in the interview. The data from each patient or patient/carer dyad was summarised with a second research team member reviewing each summary against the data. To qualitatively understand the variation in the experience of what was considered important for recovery, we compared these summaries.

Results

Twenty one patients were interviewed on one occasion and 10 were interviewed twice giving a total of 31 patient participants and 41 interviews. Of the 31 patient participants, 20 (64.5%) were female, the mean age was 81.5 years (SD 9.2, range 61-96) and 12 (39%) scored less than eight on the AMTS. Of the 41 interviews, 24 were conducted three to nine weeks, and 17 were conducted 14 to 23 weeks after the hip fracture. Nineteen interviews were with the patient only, 14 with carer only, and eight with patient/carer dyads. Interviews lasted between 20 and 90 minutes. Despite framing the interview for interviewees as exploring the

For peer review only

experience of hip fracture, many interviewees talked about general health issues. Although we prompted to clarify what was related to their fracture, in many interviews it was difficult to disentangle the impact of the fracture from the impact of other health problems. Some interviews contained almost no data that was clearly related to the fracture. From the perspective of the patient, all their health problems were part of one experience. The absence of data clearly related to the fracture was more marked in the four month compared to four week interviews. We therefore decided not to attempt interviews at 12 months post fracture as originally planned (8). The following sections report our analysis. Illustrative quotations from data are labelled with the age and gender of the patient, time since hip fracture and whether the quotation was from the patient or carer.

What is important to patients when evaluating their recovery?

From our systematic search of the interviews for data related to recovery from the hip fracture we identified the following themes: mobility, valued day-to-day activities, self-care, pain, mental wellbeing, fear of falling and leg shortening. When talking about mobility, day-to-day activities or self-care participants also talked about their level of independence.

Mobility

This was the most prominent theme, although when talking about mobility the interviewees often mentioned other themes. Mobile participants reported limited mobility in the weeks post operation and valued any improvement.

> I'm walking with a walking stick at the moment. I've been down the park and back…I can usually get around [the house] without the walking stick, and I can get up and down stairs no problem. I get upstairs with my good leg and downstairs with my bad leg. (Participant 6, male, age 78, 5 weeks post operation)

By four months, for many participants mobility had improved, and they were happy that they were returning to normal mobility.

> I can't rush round like I did, but eventually that will come…I mean it's pretty normal now, but I think it's going to be a while before I can actually walk as I did and I probably won't walk as I did… when I came home [from hospital] I was still hobbling... but now I'm more or less…walking normal, especially with the stick (Participant 10, female, age 83, 18 weeks post operation)

For those with limited mobility before hip fracture any unaided improvement was limited to the pre-fracture level but also valued.

For peer review only

> The operation was successful and got him back to normal right from the start, right from the very first day that he had it done. He was able to then walk pain free with a Zimmer frame to the toilet. The staff were all saying it was amazing how well he was walking and he would soon be back to normal, but what they didn't realise was that he was walking normally. (Carer of participant 1, male, age 84, 16 weeks post operation)

Other participants were using mobility aids that they had not been using regularly before the fracture. For some, the addition of mobility aids enabled greater security of mobility than prior to their fracture.

> Her mobility's getting better. I think she'll cope with the frame. She's had a couple of falls in the home, earlier when she was forgetting that she had to use the frame. She'd get out of bed and not use the frame and consequently fall. But she's got in the habit of using it now… she's not falling, which is a bonus. (Carer of participant 13, female, age 87, 14 weeks post operation)

Valued day-to-day activities

Those who were active prior to their fracture talked about the frustration of the restriction in their activities particularly in the weeks following the fracture.

> I'm back on what I call domestic duties – washing up! But the thing that is frustrating is that I can't get outside and do any gardening. (Participant 12, male, age 78, 6 weeks post operation)

> I just miss getting up and getting out. I never stayed in. I'd go out in the morning and come back and then I'd go out again, I just used to go out looking round the shops. I just get these crossword books and I do those. (Participant 20, female, age 92, 5 weeks post operation)

Participants who were active before their fracture were usually able to resume valued activities but had some limitations which remained a frustration.

> I can do little (gardening) jobs but because I haven't got as much movement in the hip joints, I find it difficult to go down on my hands and knees…If I go down on one knee it's difficult to get up again so that's not possible but I can do things that are higher up, I can trim. (Participant 15, female, age 61, 15 weeks post operation)

> I'm tackling a little bit of cooking now. I started to cook myself some nice lunches and I haven't got round to the… scones … I made one lot when I came home and I thought, I can't be bothered anymore. (Participant 10, female, age 83, 18 weeks post operation)

Some participants returned to valued activities through adapting how they did them, this participant using a wheelchair for the first time.

> Over the last three weeks, when we go out shopping now, I can't go down the aisles, so [daughter] gets me a (wheel)chair and I can sit in the chair and then say what

For peer review only

> shopping I need, that is very good. (Participant 9, female, age 92, 18 weeks post operation)

Participants who no longer undertook valued activities that involved significant mobility were content to continue as they were, for example, occupying themselves with visits from family and reading.

Personal care

Washing, dressing and getting to the toilet was talked about in interviews, but in many cases it was not clear whether difficulties with personal care were specifically due to the fracture. A few interviewees talked about problems with incontinence but again it was unclear whether this was specific to the fracture. Most patients had a commode or had arranged to sleep near the bathroom in the weeks immediately after the fracture. Some participants were able to describe problems with self-care specific to the hip fracture.

> I'm …not able to put a sock or anything on my injured leg. I can manage now with my trouser leg and throw these jogging trousers and hook my leg into them but I have to ask my husband if I need to put a sock or a shoe, or my slipper on that foot. (Participant 15, female, age 61, 6 weeks post operation)

At the second interview this participant was pleased to report that she now needed very little help with self-care, at least in part through wearing alternative footwear.

> I still have to throw my clothes and hook them onto the foot to get dressed. I couldn't wear lace-up shoes or anything like that because I couldn't tie them up, but things like slip-ons and sandals I can get on quite easily, so I'm fairly independent – I am independent really, I just need help with cutting my toenails and that – those on the right foot that's all. (Participant 15, female, age 61, 15 weeks post operation)

Pain

Although pain was talked about by some interviewees it was not considered a major problem.

> So here I am, four or five weeks [post operation], I get a little bit of pain, not a lot. (Participant 7, female, age 70, 5 weeks post operation)

> The pain was so bad before I had it done, and I just couldn't believe the relief after the operation when I was walking in the hospital and I had one of those pushers you know. And there was no pain. And I kept thinking, I can't believe this, and that's how it's been. I've never had any pain, not at all.
> (Participant 10, female, age 83, 18 weeks post operation)

> There's several times, like when I have got to get up those steps. I put my right foot first and bring my left foot up, and once or twice… you step on your left, and it's still

For peer review only

> there, lets you know it's still tender. (Participant 12, male, age 78, 16 weeks post operation)

Mental wellbeing

Low mood or depression associated with the reduced mobility due to the fracture was reported by a few interviewees, emphasising the great value placed by interviewees on being independently mobile.

> He can't walk and that, to him he'd rather die. I'll be honest with you he's said it once or twice, "Let me go". And I said, "No you're not going no-where". And then the other day for the first time, but he hasn't said it since, "I'm going to commit suicide", I said, "No you're not, you're not". (Carer of participant 31, male, age 84, 5 week post operation)

> For me it was a massive problem and caused me depression. To me is the most important thing, the mental aspect of taking away somebody's freedom to be able to move around and go to the shops and do all that sort of thing.
> (Participant 7, female, age 70, 23 weeks post operation)

Fear of falling

The experience of the fracture left a few participants with a fear of falling and sustaining a further fracture.

> I think it frightened him more than anything else. He's frightened he'll fall over again and do it again, that bothers him more than anything else. Because now when he stands up at all to try and walk he's frightened he's going to fall over and the same thing will happen all over again. (Carer of participant 11, male, age 84, 7 weeks post operation)

> I've got to watch what I'm doing. If I catch my foot on [paving stone], I can go over again. (Participant 12, male, age 78, 16 weeks post operation)

The fear of falling was sometimes expressed by a family member. When talking about his frustration at not being able to work in the garden, participant 6 added

> All the rain has made it very slippery, and [wife] says, "No way do you go out there."
> (Participant 12, male, age 78, 6 weeks post operation)

This emphasises the value given to mobility without falls or fear of falls by interviewees.

Leg shortening

This is a problem that is common following extra-capsular fracture of the proximal femur. One interviewee described her concerns about this.

> One leg is now shorter than the other so that makes walking a bit difficult because it gives me back pain. (Participant 15, female, age 61, 15 weeks post operation)

For peer review only

<u>Is there variation within this population of the experience of what is considered important in recovery from hip fracture?</u>

Our sample included patients from across a spectrum that extended from those who were physically and mentally active prior to their fracture through to those who, pre-fracture, had been immobile due to conditions such as multiple sclerosis, chronic obstructive airways disease and arthritis, and those with severe cognitive impairment. Although when talking about what was important to them when evaluating their recovery from hip fracture, patients from across this spectrum talked about similar themes, their experiences of what was important varied. In Box 1 we present condensed versions of the interview summaries developed during our second analysis approach, for participants chosen to represent the whole spectrum of patients. We indicate whether the data was provided by patient, carer or both.

Recovery as a return to pre-fracture state or as part of aging and decline

Every patient interviewed had experienced a hip fracture and surgery, so in physical terms all of them had, for a period of time, been somewhat impaired compared to their pre-fracture state. Four weeks post-operation, those who were active pre-fracture talked in terms of regaining a recovered state that was similar to their pre-fracture state although with some minor adaptations (participants 15 and 20 in box 1). Whilst these participants expressed worry about how well they might function in the future, there was, nevertheless, determination to progress to as full a recovery as possible. Four months post-operation many of these participants had all but regained their pre-fracture level of activity. Among participants with severely limited mobility pre-fracture, some were able to identify specific activities which were more difficult post-fracture than pre-fracture, such as putting on socks and getting in and out of bed. Some were also able to identify specific improvements in mobility post operation (see participants 9 and 15 in box 1). These participants described a process of recovery although it was very limited.

In contrast, for other participants, the fracture was just one part of a process of aging and decline. For example, participant 11 (see box 1) had been very limited in his activities before the fracture. Post fracture he needed adaptations to his home and increased care support post fracture to enable him to continue to manage at home. The mobility of participant 18 had declined and she had started using a wheelchair instead of her mobility scooter to get out of the house. However, it was unclear whether the decline was due to the concurrent heart

For peer review only

failure or the fracture. Those who were the most physically or cognitively impaired pre-fracture did not talk about regaining a recovered state but about a state of no change. They continued with their limited activities as before (for example: participants 23 and 26 in box 1). For one participant, the only change was her move to a new nursing home (participant 5 in box 1). Participants with cognitive impairment were often unaware of having experienced a fracture (Participant 1 box 1).

Recovery through adaptation

In the face of their physical limitations, most participants made adaptations that mitigated the effect of the fracture; for example employing a cleaner, moving to a nursing home or using a walking aid or other assistive device. For those who were active pre-fracture, adaptation was mostly considered temporary, although at 4 months there was some evidence that active patients had adapted to some limitations such as being unable to kneel for gardening or limiting time spent shopping to avoid exhaustion. For some participants who had been experiencing decline in their mobility pre-fracture, the fracture precipitated adaptations that they had not previously considered but made their life easier. These included using a wheelchair for shopping, having a new ramp built for getting in and out of the house in a wheelchair, using a walking aid or employing professional carers to assist with personal care. For some, their own or their carer's fear of further falls limited their mobility or at least limited how far they tested their ability to walk. Poor weather conditions exacerbated this fear, but adaptations to the environment such as walking aids or handrails lessened the fear.

Discussion

Following hip fracture, for those who had some pre-fracture mobility and able to articulate what they value during recovery, stable mobility, that is, mobility without the experience of or fear of falling, and mobility that that allows people to undertake valued activities are most valued. The ability to walk is important but so too are other leg movements needed for activities such as gardening or using transport. For some participants, maintaining mobility, however limited, was achieved by using assistive devices or working out new ways of doing an activity. Some participants adapted to their limitations, for example wearing different footwear or adjusting their expectations of what they could achieve. Others maintained their previous limited function through increased care provision.

For peer review only

Patients also consistently valued certain basic domains of health, such as pain (or lack of it), day-to-day activities, personal care and mental well-being. However, many participants in this study were unable to articulate what was important to them in terms of recovery from hip fracture. The hip fracture was just one part of their decline with age and its impact could not be disentangled from the impact of other health issues. The level of recovery perceived by a participant was influenced by their pre-fracture state and their ability to make adaptions during recovery.

Strengths and weaknesses of the study

When the mortality rate post operation is taken into account, including the higher mortality amongst older females, the study sample was broadly representative of the age profile and gender balance of the population of England, Wales and Northern Ireland experiencing hip fractures (2). We used a higher cut off for assessment of cognitive impairment (score of 8 on AMTS) compared to the NHFD (score of 6 on AMTS). This is likely to explain our higher proportion of participants with cognitive impairment compared to the average in the NHFD.

More research time was spent on recruitment than any other aspect of the study as it proved difficult. When contacted about the interview study, potential participants talked about other priorities or concerns that prevented them agreeing to interview, or they simply did not wish to be interviewed. It is possible that those not interviewed were struggling most with recovery. Our data is also limited by the difficulty some frail older adults have in giving a detailed account of their health experience (18). Interview data is jointly constructed by interviewer and interviewee (19) and our interviewer had no clinical knowledge of hip fractures. This reduced the likelihood of the interviewer influencing the data. A clinician undertaking the interviews would have the knowledge to help the patient tease out whether health problems were fracture related or not. However, this would have obscured the important finding, that participants often experienced their fracture as part of, rather than separate to, their other existing health problems. We relied on carer's accounts for some participants. We found they talked about the same themes as the participants. However, for those with cognitive impairment, some carers were unable to provide detailed data as they had limited day-to-day contact with the participant.

Comparison with other studies

For peer review only

There are similarities between our findings and other qualitative studies of similar populations. A Swedish team that explored engagement with rehabilitation post hip fracture found a similar spectrum of participants (20). They classified their participants as: those who were frail and in need of support but did not request it; those who were dependent and took no active part in rehabilitation and those who were self-sufficient. Another Swedish study, undertaken with people 12 months after their hip fracture found that mobility and a return to normal activities were key outcomes for patients (21). An Australian study of mobility post-fracture found that reduced level of mobility was associated with fear of falling, physical limitations from other illness and social/environmental factors (22). Our results also echo findings from across the research literature on the experience of health and illness. For example, the difficulty disentangling the impact of one health condition from other co-morbidities has been found for mental health conditions (23). The acceptance of an acute health problem as being part of the aging process has been found for conditions such as stroke (24). Recalibration to altered circumstances in response to a sudden injury has also been described (25), as have the adaptations- both physical and psychological- that people make in order to maintain their quality of life (26). Reduced expectations of health and acceptance of limited function have been described among elderly women (27). Fear of falling is common among older people generally (28). The consistency between our findings and other studies suggests that there is now sufficient qualitative evidence to inform policy decisions about the choice of appropriate PROMS for assessing recovery from hip fracture.

Implications for clinicians and policymakers

This study was undertaken in response to a potential policy change involving the use of a PROM to assess patient recovery from hip fracture, the results of which would form part of the evaluation of the quality of care provided for hip fracture. For the population experiencing fragility hip fractures, it is unlikely that a single PROM specific to hip fracture could be developed which is relevant to the whole spectrum of patients. Several of the themes described by interviewees - mobility, day-to-day activities, self-care, pain and mental wellbeing, are similar to the domains included in currently available generic measures including the EuroQoL EQ-5D (13), the Short Form 36-item Health Survey (SF-36) (29) and the WHOQoL-BREF (30). Both the EQ-5D (3L) and the SF-36 (version 1) have been widely used in trials of people sustaining hip fractures, but for both measures evidence of essential measurement and practical properties is limited (7). In the context of a clinical trial where patients are randomised to an intervention and control arm, these generic measures may be

For peer review only

appropriate but they may need to be supplemented by specific tools for selected groups, such as patients with high-levels of pre-injury function.

In the context of assessing quality of care for a patient population as diverse as those experiencing hip fracture, it may be impossible to devise a single PROM that will be appropriate for all patients. Although quality of care may be one factor that will influence recovery as perceived by a patient, their pre-fracture state, adaptations that they or their carers make to their reduced mobility, and their perception of whether or not they are at the stage in life where decline is inevitable will all influence how they answer questions contained within a PROM.

For peer review only

61 year old female social worker who lives with her husband. Before her fracture she was working full time and, for recreation, taking country walks, undertaking all types of gardening activities and playing with her grandchildren. Post fracture fixation (6 weeks) she described using crutches to get around the garden and shops, needing help with putting on socks and cutting toe nails, and was unable to climb stairs. She talked in terms of improvement and expectation of returning to work and full activity including cleaning and gardening. By the second interview she was frustrated that recovery was so slow but she could identify the ways in which she had continued to recover. (Participant 15, interviewed 6 weeks and 15 weeks post operation)

92 year old female who lives alone in her own flat within a sheltered housing complex. Prior to the hip fracture she looked after herself and did her own washing, but had a cleaner to undertake heavy household chores. She spent most of each day out and about at the shops, engaging in social activities, bingo and on outings. She had no other illnesses. Post-fracture fixation she talked about having some initial pain and problems lifting her leg after the operation but was now mobile about her home with a walking frame. The housing complex has a lift which she now used. She was intending to return to getting out and about as she was before her fracture. (Participant 20, interviewed 5 weeks post operation)

92 year old female lives alone with husband. Daughter visits several times a week to help. Poor hearing. Difficult to disentangle what was before and after fracture. Seems to have been able to walk around house, undertake self-care and microwave own meals pre-fracture. Post fixation of the hip fracture, patient slowly improved walking. Life seems very similar to before fracture except need for walking aid, inability to put on socks and husband now microwaves the meals. (Participant 9, interviewed 9 weeks post operation)

70 year old male retired painter and decorator who lives with his wife and enjoys almost daily visits from his grandchildren. Mobility restricted to 5-6 metres for more than two years prior to fracture due to knee pain and chronic obstructive pulmonary disease. When interviewed he describes struggling to get up the stairs, get in and out of bed, put his shoes and socks on, and bend down. Although his mobility was severely restricted prior to his fracture, he described being unable get around as much as he had done before the fracture. He noted some improvement over recent weeks, as he no longer needed two sticks for walking, only one.
(Participant 3, interviewed 15 weeks post operation)

84 year old male with dementia, who has some lucid moments and some recall of falling and hurting himself. He lives with his wife who looks after him and they have a cleaner to do heavy housework. Wife provided interview, involving the patient in the latter half when he woke up. Patient's walking was gradually slowing and he had a number of falls before his fracture. Fracture occurred while walking in shopping area with his wife. Since fixation of the fracture patient has required assistance with personal care, has professional carers four times a day, and the bathroom has been adapted for his limited mobility. The interviewee had difficulty distinguishing decline due to old age and change due to the fracture. The patient complained of some pain but it was unclear whether this was from the fracture or previously established osteoarthritis. Before the fracture both patient and wife had ceased all non-essential activities except for a weekly trip to the shops so daily life had changed little except for more care provision. (Participant 11, interviewed 7 weeks post operation)

For peer review only

74 year old female who lives with husband. Patient lived with severe rheumatoid arthritis for 30 years. Developed heart failure and admitted to hospital with shortness of breath and confusion. Fell while in hospital and fractured her hip. Mobility before hip fracture very limited – able to walk slowly in house and garden, undertake light chores, and use scooter to go shopping. Became worse with breathing difficulty. Mobility remained reduced after hospital admission. Able to take step slowly in house with support. Uses wheelchair to go out of house – a new ramp improved this by second interview. Unclear how much mobility change was due to the fracture and how much due to heart failure. (Participant 18, interviewed 6 weeks and 18 weeks post operation)

88 year old female retired teacher, who lives with her son and has a diagnosis of multiple sclerosis. The patient wove together pre and post injury experience in her account, making it difficult to disentangle. She said her son does the cooking and cleaning and her daughter assists with self-care. She has a close family, feels well supported and has lots of visitors – friends, grandchildren and great grandchildren. Her main interest beyond seeing friends and family is reading. She described being content with life. Prior to her fracture she was unwell with an infection and recounts using a frame for mobility which she still uses. (Participant 23, interviewed 5 weeks post operation)

85 year old female living in a nursing home. Her daughter visits alternate days. Her daughter provided the interview data. The patient has dementia but otherwise had been well before the fracture. Patient gets up and walks about herself, and takes herself to the toilet. She enjoys sitting and chatting. The patient does not remember the injury. Her life has not changed from how it was pre injury. The daughter did not mention any fracture-specific issues related to recovery. (Participant 26, interviewed 6 weeks post operation)

84 year old female with limited English language. Pre-injury she had carers to assist her with all her personal needs. The injury had occurred whilst being hoisted. Post injury her main concern was that at discharge from hospital, after a three month stay, she was sent to a nursing home where she knew no-one. The patient repeatedly expressed distress about being in the nursing home but did not talk about the fracture.
(Participant 5, interviewed 18 weeks post operation)

84 year old male who has dementia. He lives alone but received visits three times a day from his son who provides meals. Son was interviewed. Arthritis of knee limited mobility before the fracture. Spent most of the day sitting. At weekends prior to fracture patient went to neighbour's house for evening meal. Patient fell and sustained fracture while walking to neighbour's house. Patient does not recall fracture. At time of interview, the patient was as mobile as pre operation limited by pain and stiffness from arthritis. Not yet visiting neighbour but this was because family was discouraging this in case he falls again rather than due to mobility. (Participant 1, interviewed 16 weeks post operation)

Box 1 Summaries of the data about individual patients and their recovery from a hip fracture

For peer review only

Competing interest statement

All authors have completed the Unified Competing Interest form at www.icmje.org/coi_disclosure.pdf (available on request from the corresponding author) and declare: funding from the National Institute of Health Research, University of Warwick, and University Hospitals Coventry and Warwickshire NHS trust.

Authorship

MC, FG, JA, XG, KH and FB contributed to the conception and design of the study. FB, VM and KD conducted the interviews. All authors contributed to analysis and interpretation of data. FG, KD and VM drafted the article and all authors revised it critically for important intellectual content. All authors gave final approval of the version to be published.

Funding

This study was funded by a Programme Development Grant from the National Institute of Health Research (RP-DG-1210-10022). This trial was co-sponsored by the University of Warwick and University Hospitals Coventry and Warwickshire NHS trust. This manuscript presents independent research. The views expressed are those of the authors.

Data sharing: no additional data available

Copyright

The Corresponding Author has the right to grant on behalf of all authors and does grant on behalf of all authors, an exclusive licence (or non exclusive for government employees) on a worldwide basis to the BMJ Publishing Group Ltd to permit this article (if accepted) to be published in BMJ editions and any other BMJPGL products and sublicences such use and exploit all subsidiary rights, as set out in our licence.

Acknowledgements

We thank Katie McGuinness, Kate Dennison, Zoe Buckingham, Catherine Richmond, Rebecca McKeown, Hayley Rice, Mamta Rana, Filo Eales and Gail McCloskey for their assistance in recruitment and all the patients for their time and effort in participating in this study.

Ethical approval

Ethical approval was granted by NHS REC London - Camberwell and St Giles (11/LO/0927) on the 18th August 2011. Further approval was obtained from the research and development department of the University Hospitals Coventry and Warwickshire NHS Trust. This research complies with the Helsinki Declaration.

Access to study data

All authors had full access to all of the data in the study and can take responsibility for the integrity of the data and the accuracy of the data analysis

For peer review only

References

1. Johnell O, Kanis JA. An estimate of the worldwide prevalence, mortality and disability associated with hip fracture. . Osteoporosis international: a journal established as result of cooperation between the European Foundation for Osteoporosis and the National Osteoporosis Foundation of the USA. 2004;15:897-902.
2. Johansen A, Wakeman R, Boulton C, et al. National Hip Fracture Database National report 2013. Clinical Effectiveness and Evaluation Unit at the Royal College of Physicians, 2013.
3. Department of Health. Update to Best Practice Tariffs: Department of Health; 2011 [5th November 2012]. Available from: http://www.dh.gov.uk/health/2011/12/bpt-update/.
4. Devlin N, Appleby J. Getting the Most Out of PROMs. Putting health outcomes at the heart of NHS decision-making. London: The King's Fund, 2010.
5. Patrick DL, Burke LB, Powers JH, et al. Patient-reported outcomes to support medical product labeling claims: FDA perspective. Value Health. 2007;10:S125-S37.
6. Fitzpatrick R. Patient-reported outcome measures and performance measurement. In: Smith P, Mossialos E, Papnicolas I, Leatherman S, editors. Performance measurement for health system improvement: experiences, challenges and prospects. Cambridge: Cambridge University Press; 2009.
7. Haywood K, Brett J, Tutton E, et al. How well are we measuring patient-centred outcomes following traumatic hip fracture? A systematic review of patient-reported outcome measures (PROMs). Royal College of Nursing International Research Conference; 2012 23-25th April 2012; London: Royal College of Nursing.
8. Griffin X, Achten J, Parsons N, et al. The Warwick Hip Trauma Evaluation - an abridged protocol for the WHiTE Study. A multiple embedded randomised controlled trial cohort study. . Bone Joint Res. 2012;1(11):310-4.
9. Hodkinson HM. Evaluation of a mental test score for the assessment of mental impairment in the elderly. Age and Ageing. 1972;1(4):233-8.
10. MacKenzie DM, Copp P, Shaw RJ, et al. Brief cognitive screening of the elderly: a comparison of the Mini-Mental State Examination (MMSE), Abbreviated Mental Test (AMT) and Mental Status Questionnaire (MSQ). Psychol Med. 1996;26(2):427-30.
11. Mental Capacity Act 2005, (2005).
12. Currie C, Fleming S, Partridge M, et al. National Hip Fracture Database National Report. 2010.
13. The EuroQoL Group. EuroQoL: a new facility for the measurement of health-related quality of life. Health Policy. 1990;16(3):199-208.
14. McConnell-Henry T, Chapman Y, Francis K. Husserl and Heidegger: Exploring the disparity. International Journal of Nursing Practice. 2009;15(1):7-15.
15. Jacelon C. Older adults' participation in research. Nurse Researcher. 2007;14(4):64-73.
16. NVivo qualitative data analysis software. 10 ed. Melbourne, Australia: QSR International Pty Ltd; 2012.
17. Borkan J. In: Crabtree BF, Miller WL, editors. Doing Qualitative Research. Second ed. London: Sage Publications; 1999.
18. Kvigne K, Gjengedal E, Kirkevold M. Gaining access to the life-world of women suffering from stroke: methodological issues in empirical phenomenological studies. Journal of Advanced Nursing. 2002;40(1):61-8.
19. Mishler E. Research interviewing: Context and narrative. Cambridge, MA: Harvard University Press; 1986.
20. Olsson LE, Nystrom AE, Karlsson J, et al. Admitted with a hip fracture: patient perceptions of rehabilitation. J Clin Nurs. 2007;16(5):853-9.
21. Ziden L, Scherman MH, Wenestam CG. The break remains - elderly people's experiences of a hip fracture 1 year after discharge. Disabil Rehabil. 2010;32(2):103-13.

For peer review only

22. Taylor NF, Barelli C, Harding KE. Community ambulation before and after hip fracture: a qualitative analysis. Disabil Rehabil. 2010;32(15):1281-90.
23. Connell J, Brazier J, O'Cathain A, et al. Quality of life of people with mental health problems: a synthesis of qualitative research. Health and Quality of Life Outcomes. 2012;10:138.
24. Faircloth CA, Boylstein C, Rittman M, et al. Sudden illness and biographical flow in narratives of stroke recovery. Sociology of Health and Illness. 2004;26(2):242-61.
25. Bury M. Chronic illness as biographical disruption. Sociology of Health and Illness. 1982;4(2):169-82.
26. Clarke P, Black S. Quality of Life Following Stroke: Negotiating Disability, Identity, and Resources. Journal of Applied Gerontology. 2005;24:319-36.
27. Chambers P. Getting Beyond 'Mustn't Grumble' and 'What Can I Expect At My Age': A Narrative Approach to Older Women's Health Stories. In: Narrative, Memory and Health. Huddersfield: University of Huddersfield; 2003. p. 127-39.
28. Scheffer AC, Schuurmans MJ, van Dijk N, et al. Fear of falling: measurement strategy, prevalence, risk factors and consequences among older persons. Age and Ageing. 2008;37(1):19-24.
29. Ware Jr. J, Gandek B. Overview of the SF-36 Health Survey and the International Quality of Life Assessment (IQOLA) Project. Journal of clinical epidemiology. 1998;51(11):1203-14.
30. Molzahn A, Kalfoss M, Schick Makaroff K, et al. Comparing the importance of different aspects of quality of life to older adults across diverse cultures. Age Ageing. 2011;40(2):192-9.

For peer review only

Evaluating recovery following hip fracture: a qualitative interview study of what is important to patients

Frances Griffiths, Professor of Medicine in Society, Warwick Medical School, University of Warwick, Coventry, CV4 7AL, UK

Victoria Mason, Research Fellow, Warwick Medical School, University of Warwick, Coventry, CV4 7AL, UK

Felicity Boardman, Mildred Blaxter Post-Doctoral Fellow, Warwick Medical School, University of Warwick, Coventry, CV4 7AL, UK

Kathyrn Dennick, Research Associate, Florence Nightingale School of Nursing and Midwifery, King's College London, SE1 8WA

Kirstie Haywood, Senior Research Fellow, Royal College of Nursing Research Institute, University of Warwick, CV4 7AL, UK

Juul Achten, Research Manager Warwick Orthopaedics, Warwick Medical School, University of Warwick, Coventry, CV4 7AL, UK

Nick Parsons, Senior Research Fellow, Statistics and Epidemiology, Warwick Medical School, University of Warwick, Coventry, CV4 7AL, UK

Xavier Griffin, Specialist Trainee Trauma and Orthopaedic Surgery, Warwick Medical School, University of Warwick, Clinical Sciences Building, Clifford Bridge Road, Coventry, CV2 2DX, UK

Matt Costa, Professor Trauma and Orthopaedics, Warwick Clinical Trials Unit, Warwick Medical School, University of Warwick, Coventry, CV4 7AL, UK

Correspondence to: F.E.Griffiths@warwick.ac.uk

For peer review only

Copyright

The Corresponding Author has the right to grant on behalf of all authors and does grant on behalf of all authors, an exclusive licence (or non exclusive for government employees) on a worldwide basis to the BMJ Publishing Group Ltd to permit this article (if accepted) to be published in BMJ editions and any other BMJPGL products and sublicences such use and exploit all subsidiary rights, as set out in our licence.

Competing interest statement

All authors have completed the Unified Competing Interest form at www.icmje.org/coi_disclosure.pdf (available on request from the corresponding author) and declare: funding from the National Institute of Health Research, University of Warwick, and University Hospitals Coventry and Warwickshire NHS trust.

Authorship

MC, FG, JA, XG, KH and FB contributed to the conception and design of the study. FB, VM and KD conducted the interviews. All authors contributed to analysis and interpretation of data. FG, KD and VM drafted the article and all authors revised it critically for important intellectual content. All authors gave final approval of the version to be published.

Acknowledgements

We thank Katie McGuinness, Kate Dennison, Zoe Buckingham, Catherine Richmond, Rebecca McKeown, Hayley Rice, Mamta Rana, Filo Eales and Gail McCloskey for their assistance in recruitment and all the patients for their time and effort in participating in this study.

Funding

This study was funded by a Programme Development Grant from the National Institute of Health Research (RP-DG-1210-10022). This trial was co-sponsored by the University of Warwick and University Hospitals Coventry and Warwickshire NHS trust. This manuscript presents independent research. The views expressed are those of the authors.

Ethical approval

Ethical approval was granted by NHS REC London - Camberwell and St Giles (11/LO/0927) on the 18th August 2011. Further approval was obtained from the research and development department of the University Hospitals Coventry and Warwickshire NHS Trust. This research complies with the Helsinki Declaration.

Access to study data

All authors had full access to all of the data in the study and can take responsibility for the integrity of the data and the accuracy of the data analysis

Data sharing: no additional data available

For peer review only

Abstract

Objective

To explore what patients consider important when evaluating their recovery from hip fracture and to consider how these priorities could be used in the evaluation of the quality of hip fracture services.

Design

Semi-structured interviews exploring the experience of recovery from hip fracture at two time points - four weeks and four months post-operative hip fixation. Two approaches to analysis: thematic analysis of data specifically related to recovery from hip fracture; ~~extraction of data into template to capture~~summarising the participant's experience overall.

Participants

31 participants recruited, of whom 20 were female and 12 were cognitively impaired. Mean age 81.5 years. Interviews provided by 19 patients, 14 carers, and 8 patient/carer dyad; 10 participants were interviewed twice.

Setting

Single major trauma centre in the West Midlands of the UK.

Results

Stable mobility (without falls or fear of falls), for valued activities was considered most important by participants who had some pre-fracture mobility and were able to articulate what they valued during recovery. Mobility was important for managing personal care, for day-to-day activities such as shopping and gardening, and maintenance of mental well-being. Some participants used assistive mobility devices or adapted to their limitations. Others maintained their previous limited function through increased care provision. Many participants were unable to articulate what they valued as hip fracture was perceived as part of their decline with age. The fracture and problems from other health conditions were an inseparable part of one health experience.

Conclusions

Patients consistently valued stable mobility and its role in other basic health domains. ~~While~~For evaluating service quality, no one patient-reported outcome measure (PROM) could consistently evaluate recovery for ~~all~~ patients with hip fracture~~, general~~. General health-related quality of life tools may provide useful information ~~for the majority of patients.~~

For peer review only

~~These~~within clinical trials but may need to be supplemented by specific tools for selected groups, especially those patients with high-levels of pre-injury function.

Key words: Hip fractures, Outcome assessment (Health Care), Interview, Frail older adults

Article summary

Article focus

- The UK NHS has identified the need to evaluate service provision for patients with a hip fracture
- There is increasing expectation that patient-reported outcome measures (PROM) are used within health service evaluation
- We asked the question: what do patients who have recently experienced a hip fracture consider important when evaluating their recovery?

Key messages

- Patients active before their fracture value ~~stable~~ mobility without falls or fear of falls, to undertake valued activities but many patients consider fracture to be part of their decline with age.
- While no one PROM could evaluate all aspects of recovery for patients with hip fracture, general health-related quality of life tools may provide useful information for the majority of patients.

Strengths and limitations

- The study sample was representative of the age profile, gender balance and dementia levels of NHS patients experiencing hip fractures
- It is possible that those not agreeing to be interviewed were struggling most with recovery.
- The data is limited by the difficulty the more physically and cognitively impaired patients had in giving a detailed account of their health experience.

For peer review only

Introduction

Fragility fracture of the proximal femur (hip fracture) is one of the greatest challenges facing the healthcare community. In 1990, a global incidence of 1.31 million was reported and was associated with 740,000 deaths ~~(1)~~(1). Hip fractures constitute a heavy socioeconomic burden worldwide. The cost of this clinical problem is estimated at 1.75 million disability adjusted life years lost; 1.4% of the total healthcare burden in established market economies ~~(1)~~(1). Among those experiencing fragility hip fracture in England, Wales and Northern Ireland, 70% are aged 80 years or older, 73% are female and 34% are cognitively impaired pre-operation. The mortality rate within 30 days of operation was 8.2% in 2013 (2).

The NHS has identified the need to evaluate the quality of service provision for patients with a hip fracture; this evaluation is conducted through the National Hip Fracture Audit Database (NHFD)(2). Currently, aspects of care such as time to surgery, length of patient stay and patient mortality in hospital and 30 day and 120 day follow up are recorded in the NHFD. These data are now used to guide payments to healthcare providers; the payment being increased if the provider supplies 'best practice' care ~~(3)~~(3). However, while important, there is interest from policy makers in the potential to enhance these currently reported data fields by including and an assessment of outcome as reported by patients. It is increasingly expected that healthcare evaluations should include domains of health that are important to patients ~~(4)~~(4), captured by well-developed patient-reported outcome measures (PROMs) which aim to assess how patients function and feel in relation to a health condition or associated treatment (5). PROMS capture information that cannot be obtained by other means (5, 6) complementing more traditional performance or process-based measures.

Our aim was to establish whether or not one PROM could be used with all patients who experience a fragility hip fracture as part of the evaluation of the quality of health care for hip fracture delivered by the NHS. For this patient group we were unable to identify a PROM specific to the assessment of hip fracture, and robust evidence of the quality and acceptability of non-hip fracture specific PROMs following completion by patients sustaining a hip fracture is limited ~~(7)~~(7). Moreover, clarity with regards to the outcomes of healthcare that these patients considers relevant and important does not exist. Appropriate and relevant PROM-based assessment should be underpinned by an understanding of what is important to patients in terms of the outcomes of healthcare. We therefore designed an interview study to

For peer review only - http://bmjopen.bmj.com/site/about/guidelines.xhtml

explore with patients and, where appropriate, their carers, what they consider to be important outcomes and to explore variation across this patient group. Our research questions were:

1. What do patients who have recently experienced a hip fracture consider important when evaluating their recovery?
2. Is there variation within this population ~~about~~of the experience of what is considered important in recovery from hip fracture?

These research questions are framed by the desire of policy makers to evaluate the quality of care for hip fracture through assessment of recovery from the perspective of the patient.

Method

Study Design

We conducted semi-structured interviews with patients and, where appropriate, their carers at two time points, at approximately four weeks and then again at four months after they had sustained a fragility hip fracture.

Identification of patients with a hip fracture

We recruited participants from an existing cohort study, the Warwick Hip Trauma Evaluation ~~(8)~~(8), that commenced January 2012. This is a cohort of all patients admitted with a hip fracture to a single major trauma centre in the West Midlands of the United Kingdom. As part of their pre-operative assessment, patients were assessed for their capacity to consent using clinical assessment and the Abbreviated Mental Test Score (AMTS) ~~(9)~~(9). The AMTS is a 10-item measure used to rapidly assess the possibility of cognitive impairment in elderly people. A score below 8 suggests cognitive impairment ~~(10)~~(10). Scores less than 8 were taken to indicate that a patient was unlikely to be able to consent for themselves. Those deemed to have capacity for consenting to surgery, based on clinical assessment and AMTS, were considered able to consent for this study. Following the emergency surgery for their fracture, those with capacity gave written consent to be approached for interview. For those deemed not to have capacity due to cognitive impairment, verbal consent was obtained from their consultee ~~(11).~~(11). Ethical approval was granted by NHS REC London - Camberwell and St Giles (11/LO/0927) on the 18th August 2011.

Sampling

For peer review only

During the data collection period for this study, February to August 2012, we purposefully sampled cohort participants who had reached 4 weeks or 4 months following their hip fracture and had consented to be approached for interview. The time points were chosen to be the same as those used for data collection for the NHFD ~~(12)~~(12). If a PROM were to be used with this patient population to assess quality of care, patients would be asked to complete the PROM at these time points. Our sampling strategy ensured a diverse mix of patients with respect to the following factors: age, gender, AMTS ~~(9)~~(9) and EQ-5D score (13).

Field Code Changed

Interview recruitment and consent process

We contacted eligible patients and carers by telephone just prior to 4 weeks and/or 4 months following hip fracture first to invite them to be interviewed, then to arrange an interview. If patients declined to participate, the reasons offered were recorded. Patients with capacity to consent were contacted directly. For those patients deemed not to have capacity, we contacted their consultee. Patients able to consent for themselves signed their own consent forms. For those unable to consent the consultee signed an agreement form and we aimed to interview a carer as well as the patient (patient/carer dyad). Carers who were interviewed signed a consent form. Recruitment continued to data saturation at the first time point. The study flow diagram is at Figure 1.

Formatted: Font: Not Italic

Interview process

Formatted: Font: Italic

Formatted: Space After: 10 pt, Line spacing: Multiple 1.15 li

We interviewed participants at their current residence (own home, residential or nursing home) or in hospital. The interviewer was trained in interviewing but did not have clinical knowledge of hip fracture, its treatment or prognosis. Where possible, patients and carers were interviewed alone, however where the carer and patient requested a joint interview (whether or not the patient had cognitive impairment), they were interviewed together. The aim of the interviews was to understand each participant's lived experience of hip fracture ~~(14)~~(14) and the influence of their social context and pre-fracture health. We use the following questions:

- What is a normal day like for you now?
- How bothersome are you finding your hip?
- What is different about your life now compared to just before your injury?
- Compared to just before your injury what has stayed the same?
- Which of these make the most difference to your life?

The interviewer encouraged participants to talk about the experience in whatever order they chose and using terms meaningful to them. Later in the interview we prompted, where necessary, for clarification about what in the patient experience was related to the hip

For peer review only

fracture. Towards the end of the interview we directly asked what was important to them in terms of recovery if this had not already been talked about by the participant~~.~~, using the following questions:

- What is important to you in terms of your recovery?
- Where would you like to see yourself in the future in relation to your recovery (i.e. the next few weeks and months)?
- If a friend or neighbour were asking you now about how well you are recovering – what has been important to you that you would tell them about?
- If a doctor or nurse was asking you now about how well you are recovering – what would be important for the doctor or nurse to ask about?

Consideration was given to the potential challenges associated with interviewing older adults, for example by giving potential participants sufficient time to decide whether or not to participate and minimising burden and fatigue through streamlining questions ~~(15)~~(15). The interview process, questions and prompts were refined by the study team during the initial stage of data collection. Questions were similar for both patient and carer. Interviews were audio recorded and transcribed verbatim. For one interview, audio recording was not feasible due to the noisy environment so extensive field notes were taken and transcribed. For all interviews the researcher made field notes to assist interpretation of the interview data.

Formatted: No Spacing

Analysis

Interview transcripts were checked, anonymised and uploaded into Nvivo software ~~(16).~~(16). Initial analysis involved data immersion, reading and re-reading each transcript~~,~~, and discussion of the interview transcripts by the research team. All team members read at least five transcripts~~. The key issues crystallised from this process~~ so all transcripts were read by at least two team members. From the data we identified and crystallised what was important for participants that was specific to hip fracture recovery ~~(17).~~(17). We found that the interviews at four weeks and four months covered very similar issues, although, as would be expected, what the participants reported about each issue four weeks and at four months ~~reporting of fracture specific~~was different, as recovery was more advanced~~. For~~ at four months. As our analysis aimed to identify what patients consider important when evaluating their recovery rather than the detail of recovery itself, we ~~therefore~~ treated all the interviews related to one participant as one set of data. During data interpretation we took account of the timing of the interview, whether the interview data was from a patient or carer or patient/carer dyad, and field notes. Two different approaches to analysis were then undertaken in response to our research questions.

For peer review only

To answer our first research question, we searched the transcripts for any mention by the participants of what was important to them during recovery from hip fracture and. These were discussed at team analysis meetings. Transcripts were then coded this textin NVivo. As coding proceeded, we reviewed these codes at our team analysis meetings and combined them into themes. After codingwe had read, discussed and then coded ten transcripts we found no additional themes were identified in the remaining data. Double coding was undertaken for one in four transcripts and coding compared and discussed to check consistency of final coding. During analysis we became aware that although the data from different participants could be coded under the same theme such as mobility, there was variation in the experience of recovery. This led us to our second research question and analysis approach.

To answer our second research question, from close reading of the first five interview transcripts, we developed and refined , from the data, a template for summarising the key issues of relevance to recovery from the experience of hip fracture recovery for each patient carer dyad. This involved considering each set of interviews as a whole, reading and rereading the text and writing a summary of the patient/carer journey and all that influenced it. We reviewed the summaries at our data analysis meetings and from these initial summaries we developed a draft template. We refined the template as we summarised and discussed further transcripts. The template included: current and recent past living arrangements and environment, day-to-day life now and in the recent past, the impact of the hip fracture and its management, what was changing in day-to-day life as they recovered, the extent to which the patient referred specifically to the fracture and their ability to engage in the interview. The data from each patient or patient/carer dyad was summarised with a second research team member reviewing each summary against the data. The summaries were then compared.To qualitatively understand the variation in the experience of what was considered important for recovery, we compared these summaries.

Results

Twenty one patients were interviewed on one occasion and 10 were interviewed twice giving a total of 31 patient participants and 41 interviews. Of the 31 patient participants, 20 (64.5%) were female, the mean age was 81.5 years (SD 9.2, range 61-96) and 12 (39%) scored less than eight on the AMTS. Of the 41 interviews, 24 were conducted three to nine weeks, and

For peer review only - http://bmjopen.bmj.com/site/about/guidelines.xhtml

17 were conducted 14 to 23 weeks after the hip fracture. Nineteen interviews were with the patient only, 14 with carer only, and eight with patient/carer dyads. Interviews lasted between 20 and 90 minutes. Despite framing the interview for interviewees as exploring the experience of hip fracture, many interviewees talked about general health issues. Although we prompted to clarify what was related to their fracture, in many interviews it was difficult to disentangle the impact of the fracture from the impact of other health problems. Some interviews contained almost no data that was clearly related to the fracture. From the perspective of the patient, all their health problems were part of one experience. The absence of data clearly related to the fracture was more marked in the four month compared to ~~two month~~four week interviews. We therefore decided not to attempt interviews at 12 months post fracture as originally planned ~~(8)~~(8). The following sections report our analysis. Illustrative quotations from data are labelled with the age and gender of the patient, time since hip fracture and whether the quotation was from the patient or carer.

What is important to patients when evaluating their recovery?

From our systematic search of the interviews for data related to recovery from the hip fracture we identified the following themes: mobility, valued day-to-day activities, self-care, pain, mental wellbeing, fear of falling and leg shortening. When talking about mobility, day-to-day activities or self-care participants also talked about their level of independence.

Mobility

This was the most prominent theme, although when talking about mobility the interviewees often mentioned other themes. Mobile participants reported limited mobility in the weeks post operation and valued any improvement.

> I'm walking with a walking stick at the moment. I've been down the park and back…I can usually get around [the house] without the walking stick, and I can get up and down stairs no problem. I get upstairs with my good leg and downstairs with my bad leg. (Participant 6, male, age 78, 5 weeks post operation)

By four months, for many participants mobility had improved, and they were happy that they were returning to normal mobility.

> I can't rush round like I did, but eventually that will come…I mean it's pretty normal now, but I think it's going to be a while before I can actually walk as I did and I probably won't walk as I did… when I came home [from hospital] I was still hobbling... but now I'm more or less…walking normal, especially with the stick (Participant 10, female, age 83, 18 weeks post operation)

For peer review only

For those with limited mobility before hip fracture any unaided improvement was limited to the pre-fracture level but also valued.

> The operation was successful and got him back to normal right from the start, right from the very first day that he had it done. He was able to then walk pain free with a Zimmer frame to the toilet. The staff were all saying it was amazing how well he was walking and he would soon be back to normal, but what they didn't realise was that he was walking normally. (Carer of participant 1, male, age 84, 16 weeks post operation)

Other participants were using mobility aids that they had not been using regularly before the fracture. For some, the addition of mobility aids enabled greater security of mobility than prior to their fracture.

> Her mobility's getting better. I think she'll cope with the frame. She's had a couple of falls in the home, earlier when she was forgetting that she had to use the frame. She'd get out of bed and not use the frame and consequently fall. But she's got in the habit of using it now… she's not falling, which is a bonus. (Carer of participant 13, female, age 87, 14 weeks post operation)

Valued day-to-day activities

Those who were active prior to their fracture talked about the frustration of the restriction in their activities particularly in the weeks following the fracture.

> I'm back on what I call domestic duties – washing up! But the thing that is frustrating is that I can't get outside and do any gardening. (Participant 12, male, age 78, 6 weeks post operation)

> I just miss getting up and getting out. I never stayed in. I'd go out in the morning and come back and then I'd go out again, I just used to go out looking round the shops. I just get these crossword books and I do those. (Participant 20, female, age 92, 5 weeks post operation)

Participants who were active before their fracture were usually able to resume valued activities but had some limitations which remained a frustration.

> I can do little (gardening) jobs but because I haven't got as much movement in the hip joints, I find it difficult to go down on my hands and knees…If I go down on one knee it's difficult to get up again so that's not possible but I can do things that are higher up, I can trim. (Participant 15, female, age 61, 15 weeks post operation)

> I'm tackling a little bit of cooking now. I started to cook myself some nice lunches and I haven't got round to the… scones … I made one lot when I came home and I thought, I can't be bothered anymore. (Participant 10, female, age 83, 18 weeks post operation)

For peer review only

Some participants returned to valued activities through adapting how they did them, this participant using a wheelchair for the first time.

> Over the last three weeks, when we go out shopping now, I can't go down the aisles, so [daughter] gets me a (wheel)chair and I can sit in the chair and then say what shopping I need, that is very good. (Participant 9, female, age 92, 18 weeks post operation)

Participants who no longer undertook valued activities that involved significant mobility were content to continue as they were, for example, occupying themselves with visits from family and reading.

Personal care

Washing, dressing and getting to the toilet was talked about in interviews, but in many cases it was not clear whether difficulties with personal care were specifically due to the fracture. A few interviewees talked about problems with incontinence but again it was unclear whether this was specific to the fracture. Most patients had a commode or had arranged to sleep near the bathroom in the weeks immediately after the fracture. Some participants were able to describe problems with self-care specific to the hip fracture.

> I'm …not able to put a sock or anything on my injured leg. I can manage now with my trouser leg and throw these jogging trousers and hook my leg into them but I have to ask my husband if I need to put a sock or a shoe, or my slipper on that foot. (Participant 15, female, age 61, 6 weeks post operation)

At the second interview this participant was pleased to report that she now needed very little help with self-care, at least in part through wearing alternative footwear.

> I still have to throw my clothes and hook them onto the foot to get dressed. I couldn't wear lace-up shoes or anything like that because I couldn't tie them up, but things like slip-ons and sandals I can get on quite easily, so I'm fairly independent – I am independent really, I just need help with cutting my toenails and that – those on the right foot that's all. (Participant 15, female, age 61, 15 weeks post operation)

Pain

Although pain was talked about by some interviewees it was not considered a major problem.

> So here I am, four or five weeks [post operation], I get a little bit of pain, not a lot. (Participant 7, female, age 70, 5 weeks post operation)

> The pain was so bad before I had it done, and I just couldn't believe the relief after the operation when I was walking in the hospital and I had one of those pushers you know. And there was no pain. And I kept thinking, I can't believe this, and that's how it's been. I've never had any pain, not at all.

For peer review only

> (Participant 10, female, age 83, 18 weeks post operation)

> There's several times, like when I have got to get up those steps. I put my right foot first and bring my left foot up, and once or twice… you step on your left, and it's still there, lets you know it's still tender. (Participant 12, male, age 78, 16 weeks post operation)

Mental wellbeing

Low mood or depression associated with the reduced mobility due to the fracture was reported by a few interviewees, emphasising the great value placed by interviewees on being independently mobile.

> He can't walk and that, to him he'd rather die. I'll be honest with you he's said it once or twice, "Let me go". And I said, "No you're not going no-where". And then the other day for the first time, but he hasn't said it since, "I'm going to commit suicide", I said, "No you're not, you're not". (Carer of participant 31, male, age 84, 5 week post operation)

> For me it was a massive problem and caused me depression. To me is the most important thing, the mental aspect of taking away somebody's freedom to be able to move around and go to the shops and do all that sort of thing.
> (Participant 7, female, age 70, 23 weeks post operation)

Fear of falling

The experience of the fracture left a few participants with a fear of falling and sustaining a further fracture.

> I think it frightened him more than anything else. He's frightened he'll fall over again and do it again, that bothers him more than anything else. Because now when he stands up at all to try and walk he's frightened he's going to fall over and the same thing will happen all over again. (Carer of participant 11, male, age 84, 7 weeks post operation)

> I've got to watch what I'm doing. If I catch my foot on [paving stone], I can go over again. (Participant 12, male, age 78, 16 weeks post operation)

The fear of falling was sometimes expressed by a family member. When talking about his frustration at not being able to work in the garden, participant 6 added

> All the rain has made it very slippery, and [wife] says, "No way do you go out there."
> (Participant 12, male, age 78, 6 weeks post operation)

This emphasises the value given to ~~stable~~ mobility without falls or fear of falls by interviewees.

Leg shortening

For peer review only

This is a problem that is common following extra-capsular fracture of the proximal femur. One interviewee described her concerns about this.

> One leg is now shorter than the other so that makes walking a bit difficult because it gives me back pain. (Participant 15, female, age 61, 15 weeks post operation)

~~Variation~~Is there variation within this population of the experience of what is considered important in ~~how patients talk about~~ recovery from ~~a~~ hip fracture?

Formatted: Font: Not Italic, Underline

Our sample included patients from across a spectrum that extended from those who were physically and mentally active prior to their fracture through to those who, pre-fracture, had been immobile due to conditions such as multiple sclerosis, chronic obstructive airways disease and arthritis, and those with severe cognitive impairment. Although when talking about what was important to them when evaluating their recovery from hip fracture, patients from across this spectrum talked about similar themes, their experiences of what was important varied. In Box 1 we present condensed versions of the interview summaries developed during our second analysis approach, for participants chosen to represent the whole spectrum of patients. We indicate whether the data was provided by patient, carer or both.

Recovery as a return to pre-fracture state or as part of aging and decline

Every patient interviewed had experienced a hip fracture and surgery, so in physical terms all of them had, for a period of time, been somewhat impaired compared to their pre-fracture state. Four weeks post-operation, those who were active pre-fracture talked in terms of regaining a recovered state that was similar to their pre-fracture state although with some minor adaptations (participants 15 and 20 in box 1). Whilst these participants expressed worry about how well they might function in the future, there was, nevertheless, determination to progress to as full a recovery as possible. Four months post-operation many of these participants had all but regained their pre-fracture level of activity. Among participants with severely limited mobility pre-fracture, some were able to identify specific activities which were more difficult post-fracture than pre-fracture, such as putting on socks and getting in and out of bed. Some were also able to identify specific improvements in mobility post operation (see participants 9 and 15 in box 1). These participants described a process of recovery although it was very limited.

For peer review only

In contrast, for other participants, the fracture was just one part of a process of aging and decline. For example, participant 11 (see box 1) had been very limited in his activities before the fracture. Post fracture he needed adaptations to his home and increased care support post fracture to enable him to continue to manage at home. The mobility of participant 18 had declined and she had started using a wheelchair instead of her mobility scooter to get out of the house. However, it was unclear whether the decline was due to the concurrent heart failure or the fracture. Those who were the most physically or cognitively impaired pre-fracture did not talk about regaining a recovered state but about a state of no change. They continued with their limited activities as before (for example: participants 23 and 26 in box 1). For one participant, the only change was her move to a new nursing home (participant 5 in box 1). Participants with cognitive impairment were often unaware of having experienced a fracture (Participant 1 box 1).

Recovery through adaptation

In the face of their physical limitations, most participants made adaptations that mitigated the effect of the fracture; for example employing a cleaner, moving to a nursing home or using a walking aid or other assistive device. For those who were active pre-fracture, adaptation was mostly considered temporary, although at 4 months there was some evidence that active patients had adapted to some limitations such as being unable to kneel for gardening or limiting time spent shopping to avoid exhaustion. For some participants who had been experiencing decline in their mobility pre-fracture, the fracture precipitated adaptations that they had not previously considered but made their life easier. These included using a wheelchair for shopping, having a new ramp built for getting in and out of the house in a wheelchair, using a walking aid or employing professional carers to assist with personal care. For some, their own or their carer's fear of further falls limited their mobility or at least limited how far they tested their ability to walk. Poor weather conditions exacerbated this fear, but adaptations to the environment such as walking aids or handrails lessened the fear.

Discussion

Following hip fracture, for those who had some pre-fracture mobility and able to articulate what they value during recovery, stable mobility, that is, mobility without the experience of or fear of falling, and mobility that that allows people to undertake valued activities are most valued. The ability to walk is important but so too are other leg movements needed for activities such as gardening or using transport. For some participants, maintaining mobility,

For peer review only

however limited, was achieved by using assistive devices or working out new ways of doing an activity. Some participants adapted to their limitations, for example wearing different footwear or adjusting their expectations of what they could achieve. Others maintained their previous limited function through increased care provision.

Patients also consistently valued certain basic domains of health, such as pain (or lack of it), day-to-day activities, personal care and mental well-being. However, many participants in this study were unable to articulate what was important to them in terms of recovery from hip fracture. The hip fracture was just one part of their decline with age and its impact could not be disentangled from the impact of other health issues... The level of recovery perceived by a participant was influenced by their pre-fracture state and their ability to make adaptions during recovery.

Strengths and weaknesses of the study

When the mortality rate post operation is taken into account, including the higher mortality amongst older females, the study sample was broadly representative of the age profile and gender balance of the population of England, Wales and Northern Ireland experiencing hip fractures (2). We used a higher cut off for assessment of cognitive impairment (score of 8 on AMTS) compared to the NHFD (score of 6 on AMTS). This is likely to explain our higher proportion of participants with cognitive impairment compared to the average in the NHFD.

More research time was spent on recruitment than any other aspect of the study as it proved difficult. When contacted about the interview study, potential participants talked about other priorities or concerns that prevented them agreeing to interview, or they simply did not wish to be interviewed. It is possible that those not interviewed were struggling most with recovery. ~~Our data is also limited by the difficulty some frail older adults have in giving a detailed account of their health experience (18).~~Our data is also limited by the difficulty some frail older adults have in giving a detailed account of their health experience (18). Interview data is jointly constructed by interviewer and interviewee ~~(19)~~(19) and our interviewer had no clinical knowledge of hip fractures. This reduced the likelihood of the interviewer influencing the data. A clinician undertaking the interviews would have the knowledge to help the patient tease out whether health problems were fracture related or not. However, this would have obscured the important finding, that participants often experienced their fracture as part of, rather than separate to, their other existing health problems. ~~For~~We relied on carer's accounts

For peer review only

for some participants. We found they talked about the same themes as the participants. However, for those with cognitive impairment, some carers were unable to ~~give a~~provide detailed ~~account of recovery due to~~data as they had limited day-to-day contact with the participant.

Comparison with other studies

There are similarities between our findings and other qualitative studies of similar populations. A Swedish team that explored engagement with rehabilitation post hip fracture found a similar spectrum of participants ~~(20)~~(20). They classified their participants as: those who were frail and in need of support but did not request it; those who were dependent and took no active part in rehabilitation and those who were self-sufficient. Another Swedish study, undertaken with people 12 months after their hip fracture found that mobility and a return to normal activities were key outcomes for patients ~~(21).~~(21). An Australian study of mobility post-fracture found that reduced level of mobility was associated with fear of falling, physical limitations from other illness and social/environmental factors ~~(22)~~(22). Our results also echo findings from across the research literature on the experience of health and illness. For example, the difficulty disentangling the impact of one health condition from other co-morbidities has been found for mental health conditions ~~(23)~~(23). The acceptance of an acute health problem as being part of the aging process has been found for conditions such as stroke (24). Recalibration to altered circumstances in response to a sudden injury has also been described ~~(25)~~(25), as have the adaptations- both physical and psychological- that people make in order to maintain their quality of life ~~(26).~~(26). Reduced expectations of health and acceptance of limited function have been described among elderly women ~~(27).~~(27). Fear of falling is common among older people generally ~~(28).~~(28). The consistency between our findings and other studies suggests that ~~we~~there is now ~~have~~ sufficient qualitative evidence to inform policy decisions about the choice of appropriate PROMS for assessing recovery from hip fracture.

Implications for clinicians and policymakers

This study was undertaken in response to a potential policy change involving the use of a PROM to assess patient recovery from hip fracture, the results of which would form part of the evaluation of the quality of care provided for hip fracture. For the population experiencing fragility hip fractures, it is unlikely that a single PROM specific to hip fracture could be developed which is relevant to the whole spectrum of patients. Several of the themes

For peer review only

described by our more active interviewees - mobility, day-to-day activities, self-care, pain and mental wellbeing, are similar to the domains included in currently available generic measures including the EuroQoL EQ-5D (13)(13), the Short Form 36-item Health Survey (SF-36) (29)(29) and the WHOQoL-BREF (30).(30). Both the EQ-5D (3L) and the SF-36 (version 1) have been widely used in trials of people sustaining hip fractures, but for both measures evidence of essential measurement and practical properties is limited (7).(7). In the context of a clinical trial where patients are randomised to an intervention and control arm, these generic measures may be appropriate but they may need to be supplemented by specific tools for selected groups, especially thosesuch as patients with high-levels of pre-injury function,.

In the context of assessing quality of care for a patient population as diverse as those experiencing hip fracture, it may be impossible to devise a single PROM that will be appropriate for all patients. Although quality of care may be one factor that will influence recovery as perceived by a patient, their pre-fracture state, adaptations that they or their carers make to their reduced mobility, and their perception of whether or not they are at the stage in life where decline is inevitable will all influence how they answer questions contained within a PROM.

For peer review only

Figure 1 Flow chart of study recruitment

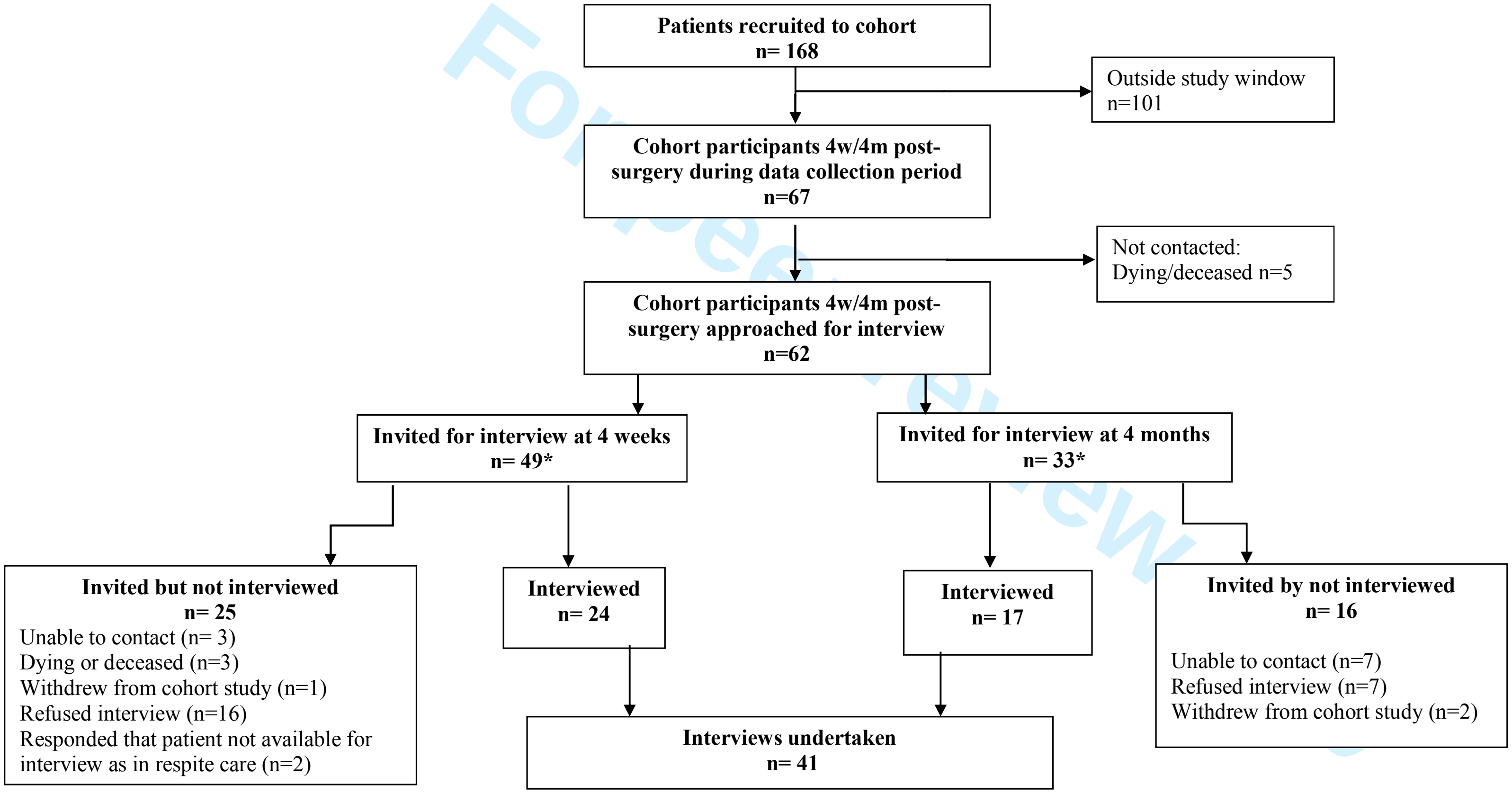

Notes: * 20 participants were invited for interview at both 4 weeks and 4 months post operation

For peer review only

Formatted Table

61 year old female social worker who lives with her husband. Before her fracture she was working full time and, for recreation, taking country walks, undertaking all types of gardening activities and playing with her grandchildren. Post fracture fixation (6 weeks) she described using crutches to get around the garden and shops, needing help with putting on socks and cutting toe nails, and was unable to climb stairs. She talked in terms of improvement and expectation of returning to work and full activity including cleaning and gardening. By the second interview she was frustrated that recovery was so slow but she could identify the ways in which she had continued to recover. (Participant 15, interviewed 6 weeks and 15 weeks post operation)
92 year old female who lives alone in her own flat within a sheltered housing complex. Prior to the hip fracture she looked after herself and did her own washing, but had a cleaner to undertake heavy household chores. She spent most of each day out and about at the shops, engaging in social activities, bingo and on outings. She had no other illnesses. Post-fracture fixation she talked about having some initial pain and problems lifting her leg after the operation but was now mobile about her home with a walking frame. The housing complex has a lift which she now used. She was intending to return to getting out and about as she was before her fracture. (Participant 20, interviewed 5 weeks post operation)
92 year old female lives alone with husband. Daughter visits several times a week to help. Poor hearing. Difficult to disentangle what was before and after fracture. Seems to have been able to walk around house, undertake self-care and microwave own meals pre-fracture. Post fixation of the hip fracture, patient slowly improved walking. Life seems very similar to before fracture except need for walking aid, inability to put on socks and husband now microwaves the meals. (Participant 9, interviewed 9 weeks post operation)
70 year old male retired painter and decorator who lives with his wife and enjoys almost daily visits from his grandchildren. Mobility restricted to 5-6 metres for more than two years prior to fracture due to knee pain and chronic obstructive pulmonary disease. When interviewed he describes struggling to get up the stairs, get in and out of bed, put his shoes and socks on, and bend down. Although his mobility was severely restricted prior to his fracture, he described being unable get around as much as he had done before the fracture. He noted some improvement over recent weeks, as he no longer needed two sticks for walking, only one. (Participant 3, interviewed 15 weeks post operation)
84 year old male with dementia, who has some lucid moments and some recall of falling and hurting himself. He lives with his wife who looks after him and they have a cleaner to do heavy housework. Wife provided interview, involving the patient in the latter half when he woke up. Patient's walking was gradually slowing and he had a number of falls before his fracture. Fracture occurred while walking in shopping area with his wife. Since fixation of the fracture patient has required assistance with personal care, has professional carers four times a day, and the bathroom has been adapted for his limited mobility. The interviewee had difficulty distinguishing decline due to old age and change due to the fracture. The patient complained of some pain but it was unclear whether this was from the fracture or previously established osteoarthritis. Before the fracture both patient and wife had ceased all non-essential activities except for a weekly trip to the shops so daily life had changed little except for more care provision. (Participant 11, interviewed 7 weeks post operation)
74 year old female who lives with husband. Patient lived with severe rheumatoid arthritis for

For peer review only

30 years. Developed heart failure and admitted to hospital with shortness of breath and confusion. Fell while in hospital and fractured her hip. Mobility before hip fracture very limited – able to walk slowly in house and garden, undertake light chores, and use scooter to go shopping. Became worse with breathing difficulty. Mobility remained reduced after hospital admission. Able to take step slowly in house with support. Uses wheelchair to go out of house – a new ramp improved this by second interview. Unclear how much mobility change was due to the fracture and how much due to heart failure. (Participant 18, interviewed 6 weeks and 18 weeks post operation)

88 year old female retired teacher, who lives with her son and has a diagnosis of multiple sclerosis. The patient wove together pre and post injury experience in her account, making it difficult to disentangle. She said her son does the cooking and cleaning and her daughter assists with self-care. She has a close family, feels well supported and has lots of visitors – friends, grandchildren and great grandchildren. Her main interest beyond seeing friends and family is reading. She described being content with life. Prior to her fracture she was unwell with an infection and recounts using a frame for mobility which she still uses. (Participant 23, interviewed 5 weeks post operation)

85 year old female living in a nursing home. Her daughter visits alternate days. Her daughter provided the interview data. The patient has dementia but otherwise had been well before the fracture. Patient gets up and walks about herself, and takes herself to the toilet. She enjoys sitting and chatting. The patient does not remember the injury. Her life has not changed from how it was pre injury. The daughter did not mention any fracture-specific issues related to recovery. (Participant 26, interviewed 6 weeks post operation)

84 year old female with limited English language. Pre-injury she had carers to assist her with all her personal needs. The injury had occurred whilst being hoisted. Post injury her main concern was that at discharge from hospital, after a three month stay, she was sent to a nursing home where she knew no-one. The patient repeatedly expressed distress about being in the nursing home but did not talk about the fracture.
(Participant 5, interviewed 18 weeks post operation)

84 year old male who has dementia. He lives alone but received visits three times a day from his son who provides meals. Son was interviewed. Arthritis of knee limited mobility before the fracture. Spent most of the day sitting. At weekends prior to fracture patient went to neighbour's house for evening meal. Patient fell and sustained fracture while walking to neighbour's house. Patient does not recall fracture. At time of interview, the patient was as mobile as pre operation limited by pain and stiffness from arthritis. Not yet visiting neighbour but this was because family was discouraging this in case he falls again rather than due to mobility. (Participant 1, interviewed 16 weeks post operation)

Box 1 Summaries of the data about individual patients and their recovery from a hip fracture

For peer review only

References

1. Johnell O, Kanis JA. An estimate of the worldwide prevalence, mortality and disability associated with hip fracture. . Osteoporosis international: a journal established as result of cooperation between the European Foundation for Osteoporosis and the National Osteoporosis Foundation of the USA. 2004;15:897-902.
2. Johansen A, Wakeman R, Boulton C, Plant F, Roberts J, Williams A. National Hip Fracture Database National report 2013. Clinical Effectiveness and Evaluation Unit at the Royal College of Physicians, 2013.
3. Department of Health. Update to Best Practice Tariffs: Department of Health; 2011 [5th November 2012]. Available from: http://www.dh.gov.uk/health/2011/12/bpt-update/.
4. Devlin N, Appleby J. Getting the Most Out of PROMs. Putting health outcomes at the heart of NHS decision-making. London: The King's Fund, 2010.
5. Patrick DL, Burke LB, Powers JH, Scott JA, Rock EP, Dawisha S, et al. Patient-reported outcomes to support medical product labeling claims: FDA perspective. Value Health. 2007;10:S125-S37.
6. Fitzpatrick R. Patient-reported outcome measures and performance measurement. In: Smith P, Mossialos E, Papnicolas I, Leatherman S, editors. Performance measurement for health system improvement: experiences, challenges and prospects. Cambridge: Cambridge University Press; 2009.
7. Haywood K, Brett J, Tutton E, Staniszewska S, editors. How well are we measuring patient-centred outcomes following traumatic hip fracture? A systematic review of patient-reported outcome measures (PROMs). Royal College of Nursing International Research Conference; 2012 23-25th April 2012; London: Royal College of Nursing.
8. Griffin X, Achten J, Parsons N, Boardman F, Griffiths F, Costa M. The Warwick Hip Trauma Evaluation - an abridged protocol for the WHiTE Study. A multiple embedded randomised controlled trial cohort study. . Bone Joint Res. 2012;1(11):310-4.
9. Hodkinson HM. Evaluation of a mental test score for the assessment of mental impairment in the elderly. Age and Ageing. 1972;1(4):233-8.
10. MacKenzie DM, Copp P, Shaw RJ, Goodwin GM. Brief cognitive screening of the elderly: a comparison of the Mini-Mental State Examination (MMSE), Abbreviated Mental Test (AMT) and Mental Status Questionnaire (MSQ). Psychol Med. 1996;26(2):427-30.
11. Mental Capacity Act 2005, (2005).
12. Currie C, Fleming S, Partridge M, Plant F, Wakeman R, Williams A. National Hip Fracture Database National Report. 2010.
13. The EuroQoL Group. EuroQoL: a new facility for the measurement of health-related quality of life. Health Policy. 1990;16(3):199-208.
14. McConnell-Henry T, Chapman Y, Francis K. Husserl and Heidegger: Exploring the disparity. International Journal of Nursing Practice. 2009;15(1):7-15.
15. Jacelon C. Older adults' participation in research. Nurse Researcher. 2007;14(4):64-73.
16. NVivo qualitative data analysis software. 10 ed. Melbourne, Australia: QSR International Pty Ltd; 2012.
17. Borkan J. In: Crabtree BF, Miller WL, editors. Doing Qualitative Research. Second ed. London: Sage Publications; 1999.
18. Kvigne K, Gjengedal E, Kirkevold M. Gaining access to the life-world of women suffering from stroke: methodological issues in empirical phenomenological studies. Journal of Advanced Nursing. 2002;40(1):61-8.
19. Mishler E. Research interviewing: Context and narrative. Cambridge, MA: Harvard University Press; 1986.

For peer review only

20. Olsson LE, Nystrom AE, Karlsson J, Ekman I. Admitted with a hip fracture: patient perceptions of rehabilitation. J Clin Nurs. 2007;16(5):853-9.
21. Ziden L, Scherman MH, Wenestam CG. The break remains - elderly people's experiences of a hip fracture 1 year after discharge. Disabil Rehabil. 2010;32(2):103-13.
22. Taylor NF, Barelli C, Harding KE. Community ambulation before and after hip fracture: a qualitative analysis. Disabil Rehabil. 2010;32(15):1281-90.
23. Connell J, Brazier J, O'Cathain A, Lloyd-Jones M, Paisley S. Quality of life of people with mental health problems: a synthesis of qualitative research. Health and Quality of Life Outcomes. 2012;10:138.
24. Faircloth CA, Boylstein C, Rittman M, Young ME, Gubrium J. Sudden illness and biographical flow in narratives of stroke recovery. Sociology of Health and Illness. 2004;26(2):242-61.
25. Bury M. Chronic illness as biographical disruption. Sociology of Health and Illness. 1982;4(2):169-82.
26. Clarke P, Black S. Quality of Life Following Stroke: Negotiating Disability, Identity, and Resources. Journal of Applied Gerontology. 2005;24:319-36.
27. Chambers P. Getting Beyond 'Mustn't Grumble' and 'What Can I Expect At My Age': A Narrative Approach to Older Women's Health Stories. In: Narrative, Memory and Health. Huddersfield: University of Huddersfield; 2003. p. 127-39.
28. Scheffer AC, Schuurmans MJ, van Dijk N, van der Hooft T, de Rooij SE. Fear of falling: measurement strategy, prevalence, risk factors and consequences among older persons. Age and Ageing. 2008;37(1):19-24.
29. Ware Jr. J, Gandek B. Overview of the SF-36 Health Survey and the International Quality of Life Assessment (IQOLA) Project. Journal of clinical epidemiology. 1998;51(11):1203-14.
30. Molzahn A, Kalfoss M, Schick Makaroff K, Skevington S. Comparing the importance of different aspects of quality of life to older adults across diverse cultures. Age Ageing. 2011;40(2):192-9.

For peer review only

Figure 1 Flow chart of study recruitment

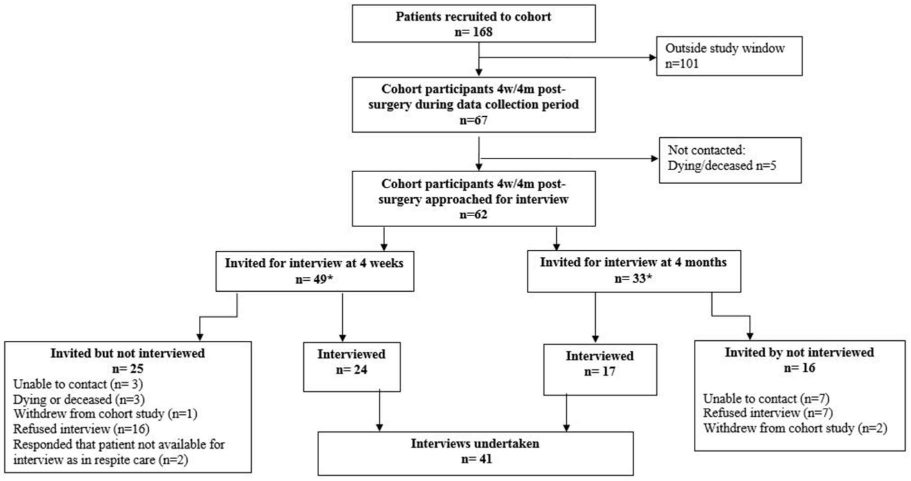

Notes: * 20 participants were invited for interview at both 4 weeks and 4 months post operation

93x63mm (300 x 300 DPI)

Evaluating recovery following hip fracture: a qualitative interview study of what is important to patients

No	Item	Guide questions/description	Included in manuscript?
**Domain 1: Research team and reflexivity**			
Personal Characteristics			
1.	Interviewer/facilitator	Which author/s conducted the interview or focus group?	yes
2.	Credentials	What were the researcher's credentials? *E.g. PhD, MD*	No place to provide this in submission process
3.	Occupation	What was their occupation at the time of the study?	yes
4.	Gender	Was the researcher male or female?	Names are ones that are usually gender specific
5.	Experience and training	What experience or training did the researcher have?	Job title provided
Relationship with participants			
6.	Relationship established	Was a relationship established prior to study commencement?	Yes
7.	Participant knowledge of the interviewer	What did the participants know about the researcher? e.*g. personal goals, reasons for doing the research*	The participant information sheet followed standard UK ethics guidelines and was approved by an ethics committee (details in submission form)
8.	Interviewer characteristics	What characteristics were reported about the interviewer/facilitator? e.g. *Bias, assumptions, reasons*	Job titles provided

For peer review only

No	Item	Guide questions/description	Included in manuscript?
		and interests in the research topic	
**Domain 2: study design**			
Theoretical framework			
9.	Methodological orientation and Theory	What methodological orientation was stated to underpin the study? *e.g. grounded theory, discourse analysis, ethnography, phenomenology, content analysis*	The description of our analysis process makes it clear that we were using a modified grounded theory approach. We describe the approach in detail rather than giving a label which could mislead.
Participant selection			
10.	Sampling	How were participants selected? *e.g. purposive, convenience, consecutive, snowball*	Yes
11.	Method of approach	How were participants approached? e.*g. face-to-face, telephone, mail, email*	Yes
12.	Sample size	How many participants were in the study?	Yes
13.	Non-participation	How many people refused to participate or dropped out? Reasons?	Yes
Setting			
14.	Setting of data collection	Where was the data collected? e.*g. home, clinic, workplace*	Yes
15.	Presence of non-participants	Was anyone else present besides the participants and researchers?	Yes
16.	Description of sample	What are the important	Yes

For peer review only

No	Item	Guide questions/description	Included in manuscript?
		characteristics of the sample? *e.g. demographic data, date*	
Data collection			
17.	Interview guide	Were questions, prompts, guides provided by the authors? Was it pilot tested?	Yes
18.	Repeat interviews	Were repeat interviews carried out? If yes, how many?	Yes
19.	Audio/visual recording	Did the research use audio or visual recording to collect the data?	Yes
20.	Field notes	Were field notes made during and/or after the interview or focus group?	Yes
21.	Duration	What was the duration of the interviews or focus group?	Yes
22.	Data saturation	Was data saturation discussed?	Yes
23.	Transcripts returned	Were transcripts returned to participants for comment and/or correction?	Not mentioned as not done with this population
**Domain 3: analysis and findings**z			
Data analysis			
24.	Number of data coders	How many data coders coded the data?	yes
25.	Description of the coding tree	Did authors provide a description of the coding tree?	First analysis approach involved thematic coding. No sub codes were used.
26.	Derivation of themes	Were themes identified in advance or derived from the data?	Yes
27.	Software	What software, if applicable, was used to manage the data?	Yes
28.	Participant checking	Did participants provide feedback on the findings?	Not mentioned as not done with this population
Reporting			
29.	Quotations presented	Were participant quotations	Yes

For peer review only

No	Item	Guide questions/description	Included in manuscript?
		presented to illustrate the themes / findings? Was each quotation identified? e*.g. participant number*	
30.	Data and findings consistent	Was there consistency between the data presented and the findings?	Yes
31.	Clarity of major themes	Were major themes clearly presented in the findings?	Yes
32.	Clarity of minor themes	Is there a description of diverse cases or discussion of minor themes?	Yes

For peer review only

BMJ Open

Evaluating recovery following hip fracture: a qualitative interview study of what is important to patients

Journal:	*BMJ Open*
Manuscript ID:	bmjopen-2014-005406.R2
Article Type:	Research
Date Submitted by the Author:	17-Oct-2014
Complete List of Authors:	Griffiths, Frances; University of Warwick, Warwick Medical School<br>Mason, Victoria; University of Warwick, Warwick Medical School<br>Boardman, Felicity; University of Warwick, Warwick Medical School<br>Dennick, Katherine; Kings College, London, Florence Nightingale School of Nursing and Midwifery<br>Haywood, Kirstie; University of Warwick, Warwick Medical School<br>Achten, Juul; University of Warwick, Warwick Medical School<br>Parsons, Nicholas; University of Warwick, Warwick Medical School<br>Griffin, Xavier; University of Warwick, Warwick Medical School<br>Costa, Matthew; University of Warwick, Warwick Medical School
**Primary Subject Heading**:	Health services research
Secondary Subject Heading:	Qualitative research, Surgery
Keywords:	Hip < ORTHOPAEDIC & TRAUMA SURGERY, Quality in health care < HEALTH SERVICES ADMINISTRATION & MANAGEMENT, QUALITATIVE RESEARCH

SCHOLARONE™
Manuscripts

For peer review only

Evaluating recovery following hip fracture: a qualitative interview study of what is important to patients

Frances Griffiths, Professor of Medicine in Society, Warwick Medical School, University of Warwick, Coventry, CV4 7AL, UK

Victoria Mason, Research Fellow, Warwick Medical School, University of Warwick, Coventry, CV4 7AL, UK

Felicity Boardman, Mildred Blaxter Post-Doctoral Fellow, Warwick Medical School, University of Warwick, Coventry, CV4 7AL, UK

Kathyrn Dennick, Research Associate, Florence Nightingale School of Nursing and Midwifery, King's College London, SE1 8WA

Kirstie Haywood, Senior Research Fellow, Royal College of Nursing Research Institute, University of Warwick, CV4 7AL, UK

Juul Achten, Research Manager Warwick Orthopaedics, Warwick Medical School, University of Warwick, Coventry, CV4 7AL, UK

Nick Parsons, Senior Research Fellow, Statistics and Epidemiology, Warwick Medical School, University of Warwick, Coventry, CV4 7AL, UK

Xavier Griffin, Specialist Trainee Trauma and Orthopaedic Surgery, Warwick Medical School, University of Warwick, Clinical Sciences Building, Clifford Bridge Road, Coventry, CV2 2DX, UK

Matt Costa, Professor Trauma and Orthopaedics, Warwick Clinical Trials Unit, Warwick Medical School, University of Warwick, Coventry, CV4 7AL, UK

Correspondence to: F.E.Griffiths@warwick.ac.uk

For peer review only

Copyright

The Corresponding Author has the right to grant on behalf of all authors and does grant on behalf of all authors, an exclusive licence (or non exclusive for government employees) on a worldwide basis to the BMJ Publishing Group Ltd to permit this article (if accepted) to be published in BMJ editions and any other BMJPGL products and sublicences such use and exploit all subsidiary rights, as set out in our licence.

Acknowledgements

We thank Katie McGuinness, Kate Dennison, Zoe Buckingham, Catherine Richmond, Rebecca McKeown, Hayley Rice, Mamta Rana, Filo Eales and Gail McCloskey for their assistance in recruitment and all the patients for their time and effort in participating in this study.

Ethical approval

Ethical approval was granted by NHS REC London - Camberwell and St Giles (11/LO/0927) on the 18th August 2011. Further approval was obtained from the research and development department of the University Hospitals Coventry and Warwickshire NHS Trust. This research complies with the Helsinki Declaration.

Access to study data

All authors had full access to all of the data in the study and can take responsibility for the integrity of the data and the accuracy of the data analysis

For peer review only

Abstract

Objective

To explore what patients consider important when evaluating their recovery from hip fracture and to consider how these priorities could be used in the evaluation of the quality of hip fracture services.

Design

Semi-structured interviews exploring the experience of recovery from hip fracture at two time points - four weeks and four months post-operative hip fixation. Two approaches to analysis: thematic analysis of data specifically related to recovery from hip fracture; summarising the participant's experience overall.

Participants

31 participants recruited, of whom 20 were female and 12 were cognitively impaired. Mean age 81.5 years. Interviews provided by 19 patients, 14 carers, and 8 patient/carer dyad; 10 participants were interviewed twice.

Setting

Single major trauma centre in the West Midlands of the UK.

Results

Stable mobility (without falls or fear of falls), for valued activities was considered most important by participants who had some pre-fracture mobility and were able to articulate what they valued during recovery. Mobility was important for managing personal care, for day-to-day activities such as shopping and gardening, and maintenance of mental well-being. Some participants used assistive mobility devices or adapted to their limitations. Others maintained their previous limited function through increased care provision. Many participants were unable to articulate what they valued as hip fracture was perceived as part of their decline with age. The fracture and problems from other health conditions were an inseparable part of one health experience.

Conclusion

Pre-fracture mobility, adaptations to reduced mobility before or after fracture, and whether or not patients' perceive themselves to be declining with age, influence what patients consider important during recovery from hip fracture. No one patient reported outcome measure (PROM) could evaluate quality of care for all patients following hip fracture. General health-related quality of life tools may provide useful information within clinical trials.

For peer review only

Key words: Hip fractures, Outcome assessment (Health Care), Interview, Frail older adults

Article summary

Article focus

- The UK NHS has identified the need to evaluate service provision for patients with a hip fracture
- There is increasing expectation that patient-reported outcome measures (PROM) are used within health service evaluation
- We asked the question: what do patients who have recently experienced a hip fracture consider important when evaluating their recovery?

Key messages

- Patients active before their fracture value mobility without falls or fear of falls, to undertake valued activities
- Many patients consider fracture to be part of their decline with age and adapt to reduced mobility or had already adapted pre-fracture
- No one patient reported outcome measure (PROM) could evaluate quality of care for all patients following hip fracture

Strengths and limitations

- The study sample was representative of the age profile, gender balance and dementia levels of NHS patients experiencing hip fractures
- It is possible that those not agreeing to be interviewed were struggling most with recovery.
- The data is limited by the difficulty the more physically and cognitively impaired patients had in giving a detailed account of their health experience.

For peer review only

Introduction

Fragility fracture of the proximal femur (hip fracture) is one of the greatest challenges facing the healthcare community. In 1990, a global incidence of 1.31 million was reported and was associated with 740,000 deaths (1). Hip fractures constitute a heavy socioeconomic burden worldwide. The cost of this clinical problem is estimated at 1.75 million disability adjusted life years lost; 1.4% of the total healthcare burden in established market economies (1). Among those experiencing fragility hip fracture in England, Wales and Northern Ireland, 70% are aged 80 years or older, 73% are female and 34% are cognitively impaired pre-operation. The mortality rate within 30 days of operation was 8.2% in 2013 (2).

The NHS has identified the need to evaluate the quality of service provision for patients with a hip fracture; this evaluation is conducted through the National Hip Fracture Audit Database (NHFD)(2). Currently, aspects of care such as time to surgery, length of patient stay and patient mortality in hospital and 30 day and 120 day follow up are recorded in the NHFD. These data are now used to guide payments to healthcare providers; the payment being increased if the provider supplies 'best practice' care (3). However, while important, there is interest from policy makers in the potential to enhance these currently reported data fields by including and an assessment of outcome as reported by patients. It is increasingly expected that healthcare evaluations should include domains of health that are important to patients (4), captured by well-developed patient-reported outcome measures (PROMs) which aim to assess how patients function and feel in relation to a health condition or associated treatment (5). PROMS capture information that cannot be obtained by other means (5, 6) complementing more traditional performance or process-based measures.

Our aim was to establish whether or not one PROM could be used with all patients who experience a fragility hip fracture as part of the evaluation of the quality of health care for hip fracture delivered by the NHS. For this patient group we were unable to identify a PROM specific to the assessment of hip fracture, and robust evidence of the quality and acceptability of non-hip fracture specific PROMs following completion by patients sustaining a hip fracture is limited (7). Moreover, clarity with regards to the outcomes of healthcare that these patients considers relevant and important does not exist. Appropriate and relevant PROM-based assessment should be underpinned by an understanding of what is important to patients in terms of the outcomes of healthcare. Further, we were concerned to understand whether,

For peer review only

for people with different pre-fracture health and social context, what was important to them during recovery was different. For example, we hypothesised that what is important to a younger, otherwise healthy person experiencing hip fracture may be different from what is important to a person who perceives themselves as nearing the end of life. Good quality care would, as far as possible, enable each patient to achieve what is important to them in terms of recovery. If a PROM is to be used to assess quality of care the measure needs to capture this. We therefore designed an interview study to explore with patients and, where appropriate, their carers, what they consider to be important outcomes and to explore variation across this patient group. Our research questions were:

1. What do patients who have recently experienced a hip fracture consider important when evaluating their recovery?
2. Is there variation between people within this population of the experience of what is considered important in recovery from hip fracture and why?

These research questions are framed by the desire of policy makers to evaluate the quality of care for hip fracture through assessment of recovery from the perspective of the patient.

Method

Study Design

We conducted semi-structured interviews with patients and, where appropriate, their carers at two time points, at approximately four weeks and then again at four months after they had sustained a fragility hip fracture.

Identification of patients with a hip fracture

We recruited participants from an existing cohort study, the Warwick Hip Trauma Evaluation (8), that commenced January 2012. This is a cohort of all patients admitted with a hip fracture to a single major trauma centre in the West Midlands of the United Kingdom. As part of their pre-operative assessment, patients were assessed for their capacity to consent using clinical assessment and the Abbreviated Mental Test Score (AMTS) (9). The AMTS is a 10-item measure used to rapidly assess the possibility of cognitive impairment in elderly people. A score below 8 suggests cognitive impairment (10). Scores less than 8 were taken to indicate that a patient was unlikely to be able to consent for themselves. Those deemed to have capacity for consenting to surgery, based on clinical assessment and AMTS, were considered able to consent for this study. Following the emergency surgery for their fracture, those with

For peer review only

capacity gave written consent to be approached for interview. For those deemed not to have capacity due to cognitive impairment, verbal consent was obtained from their consultee (11). Ethical approval was granted by NHS REC London - Camberwell and St Giles (11/LO/0927) on the 18th August 2011.

Sampling

During the data collection period for this study, February to August 2012, we purposefully sampled cohort participants who had reached 4 weeks or 4 months following their hip fracture and had consented to be approached for interview. The time points were chosen to be the same as those used for data collection for the NHFD (12). If a PROM were to be used with this patient population to assess quality of care, patients would be asked to complete the PROM at these time points. Our sampling strategy ensured a diverse mix of patients with respect to the following factors: age, gender, AMTS (9) and EQ-5D score (13).

Interview recruitment and consent process

We contacted eligible patients and carers by telephone just prior to 4 weeks and/or 4 months following hip fracture first to invite them to be interviewed, then to arrange an interview. If patients declined to participate, the reasons offered were recorded. Patients with capacity to consent were contacted directly. For those patients deemed not to have capacity, we contacted their consultee. Patients able to consent for themselves signed their own consent forms. For those unable to consent the consultee signed an agreement form and we aimed to interview a carer as well as the patient (patient/carer dyad). Carers who were interviewed signed a consent form. Initial analysis commenced during recruitment phase; recruitment continued until data saturation at the first time point. The study flow diagram is at Figure 1.

Interview process

We interviewed participants at their current residence (own home, residential or nursing home) or in hospital. The interviewer was trained in interviewing but did not have clinical knowledge of hip fracture, its treatment or prognosis. Where possible, patients and carers were interviewed alone, however where the carer and patient requested a joint interview (whether or not the patient had cognitive impairment), they were interviewed together. The aim of the interviews was to understand each participant's lived experience of hip fracture (14) and the influence of their social context and pre-fracture health. We use the following questions:

- What is a normal day like for you now?

For peer review only

- How bothersome are you finding your hip?
- What is different about your life now compared to just before your injury?
- Compared to just before your injury what has stayed the same?
- Which of these make the most difference to your life?

The interviewer encouraged participants to talk about the experience in whatever order they chose and using terms meaningful to them. Later in the interview we prompted, where necessary, for clarification about what in the patient experience was related to the hip fracture. Towards the end of the interview we directly asked what was important to them in terms of recovery if this had not already been talked about by the participant, using the following questions:

- What is important to you in terms of your recovery?
- Where would you like to see yourself in the future in relation to your recovery (i.e. the next few weeks and months)?
- If a friend or neighbour were asking you now about how well you are recovering – what has been important to you that you would tell them about?
- If a doctor or nurse was asking you now about how well you are recovering – what would be important for the doctor or nurse to ask about?

Consideration was given to the potential challenges associated with interviewing older adults, for example by giving potential participants sufficient time to decide whether or not to participate and minimising burden and fatigue through streamlining questions (15). The interview process, questions and prompts were refined by the study team during the initial stage of data collection, particularly adding questions and prompts to focus the participant on recovery from their hip fracture. Questions were similar for both patient and carer. Interviews were audio-recorded. For one interview, audio recording was not feasible due to the noisy environment so extensive field notes were taken. For all interviews the researcher made reflective field notes to assist interpretation of the interview data.

Analysis

Interviews and field notes were transcribed and transcripts checked, anonymised and uploaded into Nvivo software (16). Initial analysis involved data immersion, reading and re-reading each transcript and discussion of the interview transcripts by the research team. Our research team was multi-disciplinary: social science, behavioural science, health science, orthopaedic surgery and statistics. All team members read at least five transcripts so all transcripts were read by at least two team members. From the data we identified and crystallised what was important for participants that was specific to hip fracture recovery (17). We found that the interviews at four weeks and four months covered very similar issues,

For peer review only

although, as would be expected, what the participants reported about each issue four weeks and at four months was different, as recovery was more advanced at four months. As our analysis aimed to identify what patients consider important when evaluating their recovery rather than the detail of recovery itself, we treated all the interviews related to one participant as one set of data. During data interpretation we took account of the timing of the interview, whether the interview data was from a patient or carer or patient/carer dyad, and field notes. (17). For data collection and analysis we took a phenomenological approach in that we sought to understand participant's experience of hip fracture recovery and the influence of their context on this (14, 18) and concurrently we took a selective realist position (19) in that we recognised hip fracture as an event identifiable by means other than through the participant's account.

We used two different approaches to analysis to answer our research questions. For the first research question, which is concerned with the whole groups of participants, we used thematic analysis (20). We searched the transcripts for any mention by the participants of what was important to them during recovery from hip fracture. These were discussed at team analysis meetings. Transcripts were then coded in NVivo. As coding proceeded, we reviewed these codes at our team analysis meetings and combined them into themes. After we had read, discussed and then coded ten transcripts we found no additional themes in the remaining data. Double coding was undertaken for one in four transcripts and coding compared and discussed to check consistency of final coding. During analysis we became aware that although the data from different participants could be coded under the same theme such as mobility, the experience of recovery was very different for different people. This led us to our second research question and analysis approach.

To answer our second research question we used cross case analysis (21). We considered each participant as an individual 'case' living within their particular context (22, 23) and through comparison of cases sought to understand how they varied. To develop our matrix for the cross case analysis (21), we closely read five participant data sets then developed, from the data, a template for summarising the experience of hip fracture recovery for each patient carer dyad. This involved considering each set of interviews as a whole, reading and rereading the text and writing a summary of the patient/carer journey and all that influenced it. We reviewed the summaries at our data analysis meetings and from these initial summaries we developed a draft template. We refined the template based on the data as we summarised

For peer review only

and discussed further transcripts. The template included: current and recent past living arrangements and environment, day-to-day life now and in the recent past, the impact of the hip fracture and its management, what was changing in day-to-day life as they recovered, the extent to which the patient referred specifically to the fracture and their ability to engage in the interview. Each of these formed a data row in our matrix with a column for each participant. The data about each patient was summarised into the template with a second research team member reviewing each summary against the data. To qualitatively understand the variation in the experience of what was considered important for recovery, we compared these summaries.

Results

Twenty one patients were interviewed on one occasion and 10 were interviewed twice giving a total of 31 patient participants and 41 interviews. Of the 31 patient participants, 20 (64.5%) were female, the mean age was 81.5 years (SD 9.2, range 61-96) and 12 (39%) scored less than eight on the AMTS. Of the 41 interviews, 24 were conducted three to nine weeks, and 17 were conducted 14 to 23 weeks after the hip fracture. Nineteen interviews were with the patient only, 14 with carer only, and eight with patient/carer dyads. Interviews lasted between 20 and 90 minutes. Despite framing the interview for interviewees as exploring the experience of hip fracture, many interviewees talked about general health issues. Although we prompted to clarify what was related to their fracture, in many interviews it was difficult to disentangle the impact of the fracture from the impact of other health problems. Some interviews contained almost no data that was clearly related to the fracture. From the perspective of the patient, all their health problems were part of one experience. The absence of data clearly related to the fracture was more marked in the four month compared to four week interviews. We therefore decided not to attempt interviews at 12 months post fracture as originally planned (8). The following sections report our analysis. Illustrative quotations from data are labelled with the age and gender of the patient, time since hip fracture and whether the quotation was from the patient or carer.

<u>What is important to patients when evaluating their recovery?</u>

From our systematic search of the interviews for data related to recovery from the hip fracture we identified the following themes: mobility, valued day-to-day activities, self-care, pain, mental wellbeing, fear of falling and leg shortening. When talking about mobility, day-to-day activities or self-care participants also talked about their level of independence.

For peer review only

Mobility

This was the most prominent theme, although when talking about mobility the interviewees often mentioned other themes. Mobile participants reported limited mobility in the weeks post operation and valued any improvement.

> I'm walking with a walking stick at the moment. I've been down the park and back…I can usually get around [the house] without the walking stick, and I can get up and down stairs no problem. I get upstairs with my good leg and downstairs with my bad leg. (Participant 6, male, age 78, 5 weeks post operation)

By four months, for many participants mobility had improved, and they were happy that they were returning to normal mobility.

> I can't rush round like I did, but eventually that will come…I mean it's pretty normal now, but I think it's going to be a while before I can actually walk as I did and I probably won't walk as I did… when I came home [from hospital] I was still hobbling... but now I'm more or less…walking normal, especially with the stick (Participant 10, female, age 83, 18 weeks post operation)

For those with limited mobility before hip fracture any unaided improvement was limited to the pre-fracture level but also valued.

> The operation was successful and got him back to normal right from the start, right from the very first day that he had it done. He was able to then walk pain free with a Zimmer frame to the toilet. The staff were all saying it was amazing how well he was walking and he would soon be back to normal, but what they didn't realise was that he was walking normally. (Carer of participant 1, male, age 84, 16 weeks post operation)

Other participants were using mobility aids that they had not been using regularly before the fracture. For some, the addition of mobility aids enabled greater security of mobility than prior to their fracture.

> Her mobility's getting better. I think she'll cope with the frame. She's had a couple of falls in the home, earlier when she was forgetting that she had to use the frame. She'd get out of bed and not use the frame and consequently fall. But she's got in the habit of using it now… she's not falling, which is a bonus. (Carer of participant 13, female, age 87, 14 weeks post operation)

Valued day-to-day activities

Those who were active prior to their fracture talked about the frustration of the restriction in their activities particularly in the weeks following the fracture.

For peer review only

> I'm back on what I call domestic duties – washing up! But the thing that is frustrating is that I can't get outside and do any gardening. (Participant 12, male, age 78, 6 weeks post operation)

> I just miss getting up and getting out. I never stayed in. I'd go out in the morning and come back and then I'd go out again, I just used to go out looking round the shops. I just get these crossword books and I do those. (Participant 20, female, age 92, 5 weeks post operation)

Participants who were active before their fracture were usually able to resume valued activities but had some limitations which remained a frustration.

> I can do little (gardening) jobs but because I haven't got as much movement in the hip joints, I find it difficult to go down on my hands and knees…If I go down on one knee it's difficult to get up again so that's not possible but I can do things that are higher up, I can trim. (Participant 15, female, age 61, 15 weeks post operation)

> I'm tackling a little bit of cooking now. I started to cook myself some nice lunches and I haven't got round to the… scones … I made one lot when I came home and I thought, I can't be bothered anymore. (Participant 10, female, age 83, 18 weeks post operation)

Some participants returned to valued activities through adapting how they did them, this participant using a wheelchair for the first time.

> Over the last three weeks, when we go out shopping now, I can't go down the aisles, so [daughter] gets me a (wheel)chair and I can sit in the chair and then say what shopping I need, that is very good. (Participant 9, female, age 92, 18 weeks post operation)

Participants who no longer undertook valued activities that involved significant mobility were content to continue as they were, for example, occupying themselves with visits from family and reading.

Personal care

Washing, dressing and getting to the toilet was talked about in interviews, but in many cases it was not clear whether difficulties with personal care were specifically due to the fracture. A few interviewees talked about problems with incontinence but again it was unclear whether this was specific to the fracture. Most patients had a commode or had arranged to sleep near the bathroom in the weeks immediately after the fracture. Some participants were able to describe problems with self-care specific to the hip fracture.

> I'm …not able to put a sock or anything on my injured leg. I can manage now with

For peer review only

> my trouser leg and throw these jogging trousers and hook my leg into them but I have to ask my husband if I need to put a sock or a shoe, or my slipper on that foot. (Participant 15, female, age 61, 6 weeks post operation)

At the second interview this participant was pleased to report that she now needed very little help with self-care, at least in part through wearing alternative footwear.

> I still have to throw my clothes and hook them onto the foot to get dressed. I couldn't wear lace-up shoes or anything like that because I couldn't tie them up, but things like slip-ons and sandals I can get on quite easily, so I'm fairly independent – I am independent really, I just need help with cutting my toenails and that – those on the right foot that's all. (Participant 15, female, age 61, 15 weeks post operation)

Pain

Although pain was talked about by some interviewees it was not considered a major problem.

> So here I am, four or five weeks [post operation], I get a little bit of pain, not a lot. (Participant 7, female, age 70, 5 weeks post operation)

> The pain was so bad before I had it done, and I just couldn't believe the relief after the operation when I was walking in the hospital and I had one of those pushers you know. And there was no pain. And I kept thinking, I can't believe this, and that's how it's been. I've never had any pain, not at all.
> (Participant 10, female, age 83, 18 weeks post operation)

> There's several times, like when I have got to get up those steps. I put my right foot first and bring my left foot up, and once or twice… you step on your left, and it's still there, lets you know it's still tender. (Participant 12, male, age 78, 16 weeks post operation)

Mental wellbeing

Low mood or depression associated with the reduced mobility due to the fracture was reported by a few interviewees, emphasising the great value placed by interviewees on being independently mobile.

> He can't walk and that, to him he'd rather die. I'll be honest with you he's said it once or twice, "Let me go". And I said, "No you're not going no-where". And then the other day for the first time, but he hasn't said it since, "I'm going to commit suicide", I said, "No you're not, you're not". (Carer of participant 31, male, age 84, 5 week post operation)

> For me it was a massive problem and caused me depression. To me is the most important thing, the mental aspect of taking away somebody's freedom to be able to move around and go to the shops and do all that sort of thing.
> (Participant 7, female, age 70, 23 weeks post operation)

Fear of falling

For peer review only

The experience of the fracture left a few participants with a fear of falling and sustaining a further fracture.

> I think it frightened him more than anything else. He's frightened he'll fall over again and do it again, that bothers him more than anything else. Because now when he stands up at all to try and walk he's frightened he's going to fall over and the same thing will happen all over again. (Carer of participant 11, male, age 84, 7 weeks post operation)

> I've got to watch what I'm doing. If I catch my foot on [paving stone], I can go over again. (Participant 12, male, age 78, 16 weeks post operation)

The fear of falling was sometimes expressed by a family member. When talking about his frustration at not being able to work in the garden, participant 6 added

> All the rain has made it very slippery, and [wife] says, "No way do you go out there." (Participant 12, male, age 78, 6 weeks post operation)

This emphasises the value given to mobility without falls or fear of falls by interviewees.

Leg shortening

This is a problem that is common following extra-capsular fracture of the proximal femur. One interviewee described her concerns about this.

> One leg is now shorter than the other so that makes walking a bit difficult because it gives me back pain. (Participant 15, female, age 61, 15 weeks post operation)

Is there variation within this population of the experience of what is considered important in recovery from hip fracture?

Our sample included patients from across a spectrum that extended from those who were physically and mentally active prior to their fracture through to those who, pre-fracture, had been immobile due to conditions such as multiple sclerosis, chronic obstructive airways disease and arthritis, and those with severe cognitive impairment. Although when talking about what was important to them when evaluating their recovery from hip fracture, patients from across this spectrum talked about similar themes, their experiences of what was important was different for different people. In Box 1 we present condensed versions of the interview summaries developed during our second analysis approach, for participants chosen to represent the whole spectrum of patients. We indicate whether the data was provided by patient, carer or both.

Recovery as a return to pre-fracture state or as part of aging and decline

For peer review only

Every patient interviewed had experienced a hip fracture and surgery, so in physical terms all of them had, for a period of time, been somewhat impaired compared to their pre-fracture state. Four weeks post-operation, those who were active pre-fracture talked in terms of regaining a recovered state that was similar to their pre-fracture state although with some minor adaptations (participants 15 and 20 in box 1). Whilst these participants expressed worry about how well they might function in the future, there was, nevertheless, determination to progress to as full a recovery as possible. Four months post-operation many of these participants had all but regained their pre-fracture level of activity. Among participants with severely limited mobility pre-fracture, some were able to identify specific activities which were more difficult post-fracture than pre-fracture, such as putting on socks and getting in and out of bed. Some were also able to identify specific improvements in mobility post operation (see participants 9 and 15 in box 1). These participants described a process of recovery although it was very limited.

In contrast, for other participants, the fracture was just one part of a process of aging and decline. For example, participant 11 (see box 1) had been very limited in his activities before the fracture. Post fracture he needed adaptations to his home and increased care support post fracture to enable him to continue to manage at home. The mobility of participant 18 had declined and she had started using a wheelchair instead of her mobility scooter to get out of the house. However, it was unclear whether the decline was due to the concurrent heart failure or the fracture. Those who were the most physically or cognitively impaired pre-fracture did not talk about regaining a recovered state but about a state of no change. They continued with their limited activities as before (for example: participants 23 and 26 in box 1). For one participant, the only change was her move to a new nursing home (participant 5 in box 1). Participants with cognitive impairment were often unaware of having experienced a fracture (Participant 1 box 1).

Recovery through adaptation

In the face of their physical limitations, most participants made adaptations that mitigated the effect of the fracture; for example employing a cleaner, moving to a nursing home or using a walking aid or other assistive device. For those who were active pre-fracture, adaptation was mostly considered temporary, although at 4 months there was some evidence that active patients had adapted to some limitations such as being unable to kneel for gardening or limiting time spent shopping to avoid exhaustion. For some participants who had been

For peer review only

experiencing decline in their mobility pre-fracture, the fracture precipitated adaptations that they had not previously considered but made their life easier. These included using a wheelchair for shopping, having a new ramp built for getting in and out of the house in a wheelchair, using a walking aid or employing professional carers to assist with personal care. For some, their own or their carer's fear of further falls limited their mobility or at least limited how far they tested their ability to walk. Poor weather conditions exacerbated this fear, but adaptations to the environment such as walking aids or handrails lessened the fear.

Discussion

Following hip fracture, for those who had some pre-fracture mobility and able to articulate what they value during recovery, stable mobility, that is, mobility without the experience of or fear of falling, and mobility that that allows people to undertake valued activities are most valued. The ability to walk is important but so too are other leg movements needed for activities such as gardening or using transport. For some participants, maintaining mobility, however limited, was achieved by using assistive devices or working out new ways of doing an activity. Some participants adapted to their limitations, for example wearing different footwear or adjusting their expectations of what they could achieve. Others maintained their previous limited function through increased care provision.

Patients also consistently valued certain basic domains of health, such as pain (or lack of it), day-to-day activities, personal care and mental well-being. However, many participants in this study were unable to articulate what was important to them in terms of recovery from hip fracture. The hip fracture was just one part of their decline with age and its impact could not be disentangled from the impact of other health issues. The level of recovery perceived by a participant was influenced by their pre-fracture state and their ability to make adaptions during recovery.

Strengths and weaknesses of the study

When the mortality rate post operation is taken into account, including the higher mortality amongst older females, the study sample was broadly representative of the age profile and gender balance of the population of England, Wales and Northern Ireland experiencing hip fractures (2). We used a higher cut off for assessment of cognitive impairment (score of 8 on AMTS) compared to the NHFD (score of 6 on AMTS). This is likely to explain our higher proportion of participants with cognitive impairment compared to the average in the NHFD.

For peer review only

More research time was spent on recruitment than any other aspect of the study as it proved difficult. When contacted about the interview study, potential participants talked about other priorities or concerns that prevented them agreeing to interview, or they simply did not wish to be interviewed. It is possible that those not interviewed were struggling most with recovery. Our data is also limited by the difficulty some frail older adults have in giving a detailed account of their health experience (24). Interview data is jointly constructed by interviewer and interviewee (25) and our interviewer had no clinical knowledge of hip fractures. This reduced the likelihood of the interviewer influencing the data. A clinician undertaking the interviews would have the knowledge to help the patient tease out whether health problems were fracture related or not. However, this would have obscured the important finding, that participants often experienced their fracture as part of, rather than separate to, their other existing health problems. We relied on carer's accounts for some participants. We found they talked about the same themes as the participants. However, for those with cognitive impairment, some carers were unable to provide detailed data as they had limited day-to-day contact with the participant. We did not attempt to check with participants about our interpretation of the data to avoid further burden for them.

Comparison with other studies

There are similarities between our findings and other qualitative studies of similar populations. A Swedish team that explored engagement with rehabilitation post hip fracture found a similar spectrum of participants (26). They classified their participants as: those who were frail and in need of support but did not request it; those who were dependent and took no active part in rehabilitation and those who were self-sufficient. Another Swedish study, undertaken with people 12 months after their hip fracture found that mobility and a return to normal activities were key outcomes for patients (27). An Australian study of mobility post-fracture found that reduced level of mobility was associated with fear of falling, physical limitations from other illness and social/environmental factors (28). Our results also echo findings from across the research literature on the experience of health and illness. For example, the difficulty disentangling the impact of one health condition from other co-morbidities has been found for mental health conditions (29). The acceptance of an acute health problem as being part of the aging process has been found for conditions such as stroke (30). Recalibration to altered circumstances in response to a sudden injury has also been described (31), as have the adaptations- both physical and psychological- that people

For peer review only

make in order to maintain their quality of life (32). Reduced expectations of health and acceptance of limited function have been described among elderly women (33). Fear of falling is common among older people generally (34). The consistency between our findings and other studies suggests that there is now sufficient qualitative evidence to inform policy decisions about the choice of appropriate PROMS for assessing recovery from hip fracture.

Implications for clinicians and policymakers

This study was undertaken in response to a potential policy change involving the use of a PROM to assess patient recovery from hip fracture, the results of which would form part of the evaluation of the quality of care provided for hip fracture. We conclude that for the population experiencing fragility hip fractures, it is unlikely that a single PROM specific to hip fracture could be developed which is relevant to the whole spectrum of patients. An assessment that focuses on mobility of the hip would be relevant for many patients, and mobility impacts on other health domains. However, with any form of assessment of mobility, pre-fracture status would have to be taken into account. Some patients had limited pre-fracture mobility at the hip so a lack of mobility during recovery may not reflect the quality of care. In addition there are other factors that influence the perception of recovery by patients. These include adaptations that they or their carers make to compensate for their reduced mobility, and patient perception of whether or not they are at the stage in life where decline is inevitable. Quality of care is only one of a number of interrelated factors that influence the patient's perception of recovery from hip fracture.

Several of the themes described by interviewees - mobility, day-to-day activities, self-care, pain and mental wellbeing, are similar to the domains included in currently available generic measures including the EuroQoL EQ-5D (13), the Short Form 36-item Health Survey (SF-36) (35) and the WHOQoL-BREF (36). Both the EQ-5D (3L) and the SF-36 (version 1) have been widely used in trials of people sustaining hip fractures, but for both measures evidence of essential measurement and practical properties is limited (7). In the context of a clinical trial where patients are randomised to an intervention and control arm, these generic measures may be appropriate but they may need to be supplemented by specific tools for selected groups, such as patients with high-levels of pre-injury function.

For peer review only

61 year old female social worker who lives with her husband. Before her fracture she was working full time and, for recreation, taking country walks, undertaking all types of gardening activities and playing with her grandchildren. Post fracture fixation (6 weeks) she described using crutches to get around the garden and shops, needing help with putting on socks and cutting toe nails, and was unable to climb stairs. She talked in terms of improvement and expectation of returning to work and full activity including cleaning and gardening. By the second interview she was frustrated that recovery was so slow but she could identify the ways in which she had continued to recover. (Participant 15, interviewed 6 weeks and 15 weeks post operation)

92 year old female who lives alone in her own flat within a sheltered housing complex. Prior to the hip fracture she looked after herself and did her own washing, but had a cleaner to undertake heavy household chores. She spent most of each day out and about at the shops, engaging in social activities, bingo and on outings. She had no other illnesses. Post-fracture fixation she talked about having some initial pain and problems lifting her leg after the operation but was now mobile about her home with a walking frame. The housing complex has a lift which she now used. She was intending to return to getting out and about as she was before her fracture. (Participant 20, interviewed 5 weeks post operation)

92 year old female lives alone with husband. Daughter visits several times a week to help. Poor hearing. Difficult to disentangle what was before and after fracture. Seems to have been able to walk around house, undertake self-care and microwave own meals pre-fracture. Post fixation of the hip fracture, patient slowly improved walking. Life seems very similar to before fracture except need for walking aid, inability to put on socks and husband now microwaves the meals. (Participant 9, interviewed 9 weeks post operation)

70 year old male retired painter and decorator who lives with his wife and enjoys almost daily visits from his grandchildren. Mobility restricted to 5-6 metres for more than two years prior to fracture due to knee pain and chronic obstructive pulmonary disease. When interviewed he describes struggling to get up the stairs, get in and out of bed, put his shoes and socks on, and bend down. Although his mobility was severely restricted prior to his fracture, he described being unable get around as much as he had done before the fracture. He noted some improvement over recent weeks, as he no longer needed two sticks for walking, only one.
(Participant 3, interviewed 15 weeks post operation)

84 year old male with dementia, who has some lucid moments and some recall of falling and hurting himself. He lives with his wife who looks after him and they have a cleaner to do heavy housework. Wife provided interview, involving the patient in the latter half when he woke up. Patient's walking was gradually slowing and he had a number of falls before his fracture. Fracture occurred while walking in shopping area with his wife. Since fixation of the fracture patient has required assistance with personal care, has professional carers four times a day, and the bathroom has been adapted for his limited mobility. The interviewee had difficulty distinguishing decline due to old age and change due to the fracture. The patient complained of some pain but it was unclear whether this was from the fracture or previously established osteoarthritis. Before the fracture both patient and wife had ceased all non-essential activities except for a weekly trip to the shops so daily life had changed little except for more care provision. (Participant 11, interviewed 7 weeks post operation)

For peer review only

74 year old female who lives with husband. Patient lived with severe rheumatoid arthritis for 30 years. Developed heart failure and admitted to hospital with shortness of breath and confusion. Fell while in hospital and fractured her hip. Mobility before hip fracture very limited – able to walk slowly in house and garden, undertake light chores, and use scooter to go shopping. Became worse with breathing difficulty. Mobility remained reduced after hospital admission. Able to take step slowly in house with support. Uses wheelchair to go out of house – a new ramp improved this by second interview. Unclear how much mobility change was due to the fracture and how much due to heart failure. (Participant 18, interviewed 6 weeks and 18 weeks post operation)

88 year old female retired teacher, who lives with her son and has a diagnosis of multiple sclerosis. The patient wove together pre and post injury experience in her account, making it difficult to disentangle. She said her son does the cooking and cleaning and her daughter assists with self-care. She has a close family, feels well supported and has lots of visitors – friends, grandchildren and great grandchildren. Her main interest beyond seeing friends and family is reading. She described being content with life. Prior to her fracture she was unwell with an infection and recounts using a frame for mobility which she still uses. (Participant 23, interviewed 5 weeks post operation)

85 year old female living in a nursing home. Her daughter visits alternate days. Her daughter provided the interview data. The patient has dementia but otherwise had been well before the fracture. Patient gets up and walks about herself, and takes herself to the toilet. She enjoys sitting and chatting. The patient does not remember the injury. Her life has not changed from how it was pre injury. The daughter did not mention any fracture-specific issues related to recovery. (Participant 26, interviewed 6 weeks post operation)

84 year old female with limited English language. Pre-injury she had carers to assist her with all her personal needs. The injury had occurred whilst being hoisted. Post injury her main concern was that at discharge from hospital, after a three month stay, she was sent to a nursing home where she knew no-one. The patient repeatedly expressed distress about being in the nursing home but did not talk about the fracture.
(Participant 5, interviewed 18 weeks post operation)

84 year old male who has dementia. He lives alone but received visits three times a day from his son who provides meals. Son was interviewed. Arthritis of knee limited mobility before the fracture. Spent most of the day sitting. At weekends prior to fracture patient went to neighbour's house for evening meal. Patient fell and sustained fracture while walking to neighbour's house. Patient does not recall fracture. At time of interview, the patient was as mobile as pre operation limited by pain and stiffness from arthritis. Not yet visiting neighbour but this was because family was discouraging this in case he falls again rather than due to mobility. (Participant 1, interviewed 16 weeks post operation)

Box 1 Summaries of the data about individual patients and their recovery from a hip fracture

For peer review only

Competing interest statement
All authors have completed the Unified Competing Interest form at www.icmje.org/coi_disclosure.pdf (available on request from the corresponding author) and declare: funding from the National Institute of Health Research, University of Warwick, and University Hospitals Coventry and Warwickshire NHS trust.

Authorship
MC, FG, JA, XG, KH and FB contributed to the conception and design of the study. FB, VM and KD conducted the interviews. All authors contributed to analysis and interpretation of data. FG, KD and VM drafted the article and all authors revised it critically for important intellectual content. All authors gave final approval of the version to be published.

Funding
This study was funded by a Programme Development Grant from the National Institute of Health Research (RP-DG-1210-10022). This trial was co-sponsored by the University of Warwick and University Hospitals Coventry and Warwickshire NHS trust. This manuscript presents independent research. The views expressed are those of the authors.

Data sharing: no additional data available

References

1. Johnell O, Kanis JA. An estimate of the worldwide prevalence, mortality and disability associated with hip fracture. . Osteoporosis international: a journal established as result of cooperation between the European Foundation for Osteoporosis and the National Osteoporosis Foundation of the USA. 2004;15:897-902.
2. Johansen A, Wakeman R, Boulton C, Plant F, Roberts J, Williams A. National Hip Fracture Database National report 2013. Clinical Effectiveness and Evaluation Unit at the Royal College of Physicians, 2013.
3. Department of Health. Update to Best Practice Tariffs: Department of Health; 2011 [5th November 2012]. Available from: http://www.dh.gov.uk/health/2011/12/bpt-update/.
4. Devlin N, Appleby J. Getting the Most Out of PROMs. Putting health outcomes at the heart of NHS decision-making. London: The King's Fund, 2010.
5. Patrick DL, Burke LB, Powers JH, Scott JA, Rock EP, Dawisha S, et al. Patient-reported outcomes to support medical product labeling claims: FDA perspective. Value Health. 2007;10:S125-S37.
6. Fitzpatrick R. Patient-reported outcome measures and performance measurement. In: Smith P, Mossialos E, Papnicolas I, Leatherman S, editors. Performance measurement for health system improvement: experiences, challenges and prospects. Cambridge: Cambridge University Press; 2009.
7. Haywood K, Brett J, Tutton E, Staniszewska S, editors. How well are we measuring patient-centred outcomes following traumatic hip fracture? A systematic review of patient-reported outcome measures (PROMs). Royal College of Nursing International Research Conference; 2012 23-25th April 2012; London: Royal College of Nursing.
8. Griffin X, Achten J, Parsons N, Boardman F, Griffiths F, Costa M. The Warwick Hip Trauma Evaluation - an abridged protocol for the WHiTE Study. A multiple embedded randomised controlled trial cohort study. . Bone Joint Res. 2012;1(11):310-4.
9. Hodkinson HM. Evaluation of a mental test score for the assessment of mental impairment in the elderly. Age and Ageing. 1972;1(4):233-8.

For peer review only

10. MacKenzie DM, Copp P, Shaw RJ, Goodwin GM. Brief cognitive screening of the elderly: a comparison of the Mini-Mental State Examination (MMSE), Abbreviated Mental Test (AMT) and Mental Status Questionnaire (MSQ). Psychol Med. 1996;26(2):427-30.
11. Mental Capacity Act 2005, (2005).
12. Currie C, Fleming S, Partridge M, Plant F, Wakeman R, Williams A. National Hip Fracture Database National Report. 2010.
13. The EuroQoL Group. EuroQoL: a new facility for the measurement of health-related quality of life. Health Policy. 1990;16(3):199-208.
14. McConnell-Henry T, Chapman Y, Francis K. Husserl and Heidegger: Exploring the disparity. International Journal of Nursing Practice. 2009;15(1):7-15.
15. Jacelon C. Older adults' participation in research. Nurse Researcher. 2007;14(4):64-73.
16. NVivo qualitative data analysis software. 10 ed. Melbourne, Australia: QSR International Pty Ltd; 2012.
17. Borkan J. In: Crabtree BF, Miller WL, editors. Doing Qualitative Research. Second ed. London: Sage Publications; 1999.
18. Leder D. A tale of two bodies: the cartesian corpse and the lived body. The body in medical thought and practice. Dordrecht: Klewer Academic Publishers; 1992. p. 17-36.
19. Miller A. Realism. In: Zalta EN, editor. The Stanford Encyclopedia of Philosophy: Stanford University; 2012.
20. Green J, Thorogood N. Qualitative Methods for Health Research. London: Sage; 2004.
21. Miles MB, Huberman AM. Qualitative data analysis. Thousand Oaks, CA: Sage; 1994.
22. Ragin CC. 'Casing' and the process of social inquiry. In: Becker CCRaH, editor. What is a case? Exploring the foundations of social inquiry. Cambridge: Cambridge University Press; 1992.
23. Griffiths F, Borkan J, Byrne D, Crabtree B, Dowrick C, Gunn J, et al. Developing evidence for how to tailor medical interventions for the individual patient. Qualitative Health Research. 2010;20(12):1629-41.
24. Kvigne K, Gjengedal E, Kirkevold M. Gaining access to the life-world of women suffering from stroke: methodological issues in empirical phenomenological studies. Journal of Advanced Nursing. 2002;40(1):61-8.
25. Mishler E. Research interviewing: Context and narrative. Cambridge, MA: Harvard University Press; 1986.
26. Olsson LE, Nystrom AE, Karlsson J, Ekman I. Admitted with a hip fracture: patient perceptions of rehabilitation. J Clin Nurs. 2007;16(5):853-9.
27. Ziden L, Scherman MH, Wenestam CG. The break remains - elderly people's experiences of a hip fracture 1 year after discharge. Disabil Rehabil. 2010;32(2):103-13.
28. Taylor NF, Barelli C, Harding KE. Community ambulation before and after hip fracture: a qualitative analysis. Disabil Rehabil. 2010;32(15):1281-90.
29. Connell J, Brazier J, O'Cathain A, Lloyd-Jones M, Paisley S. Quality of life of people with mental health problems: a synthesis of qualitative research. Health and Quality of Life Outcomes. 2012;10:138.
30. Faircloth CA, Boylstein C, Rittman M, Young ME, Gubrium J. Sudden illness and biographical flow in narratives of stroke recovery. Sociology of Health and Illness. 2004;26(2):242-61.
31. Bury M. Chronic illness as biographical disruption. Sociology of Health and Illness. 1982;4(2):169-82.
32. Clarke P, Black S. Quality of Life Following Stroke: Negotiating Disability, Identity, and Resources. Journal of Applied Gerontology. 2005;24:319-36.
33. Chambers P. Getting Beyond 'Mustn't Grumble' and 'What Can I Expect At My Age': A Narrative Approach to Older Women's Health Stories. In: Narrative, Memory and Health. Huddersfield: University of Huddersfield; 2003. p. 127-39.

For peer review only

34. Scheffer AC, Schuurmans MJ, van Dijk N, van der Hooft T, de Rooij SE. Fear of falling: measurement strategy, prevalence, risk factors and consequences among older persons. Age and Ageing. 2008;37(1):19-24.
35. Ware Jr. J, Gandek B. Overview of the SF-36 Health Survey and the International Quality of Life Assessment (IQOLA) Project. Journal of clinical epidemiology. 1998;51(11):1203-14.
36. Molzahn A, Kalfoss M, Schick Makaroff K, Skevington S. Comparing the importance of different aspects of quality of life to older adults across diverse cultures. Age Ageing. 2011;40(2):192-9.

For peer review only

Figure 1 Flow chart of study recruitment

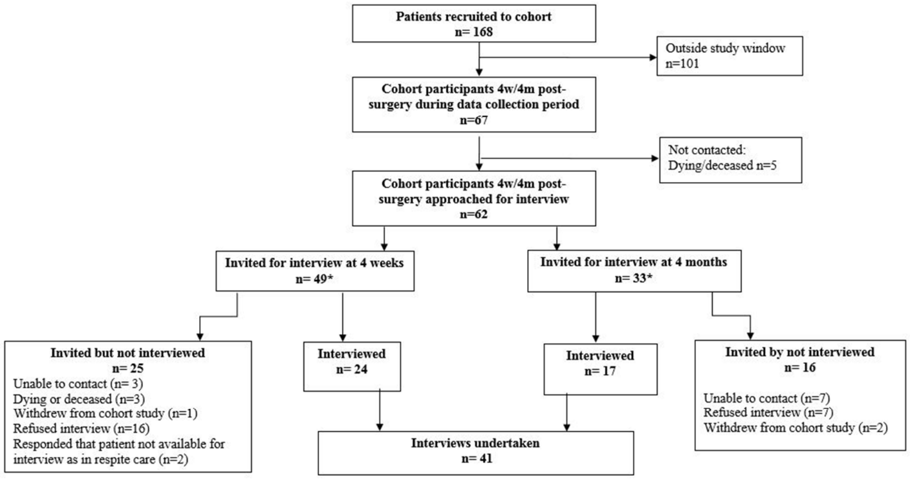

Notes: * 20 participants were invited for interview at both 4 weeks and 4 months post operation

93x63mm (300 x 300 DPI)

view only

Evaluating recovery following hip fracture: a qualitative interview study of what is important to patients

Frances Griffiths, Professor of Medicine in Society, Warwick Medical School, University of Warwick, Coventry, CV4 7AL, UK

Victoria Mason, Research Fellow, Warwick Medical School, University of Warwick, Coventry, CV4 7AL, UK

Felicity Boardman, Mildred Blaxter Post-Doctoral Fellow, Warwick Medical School, University of Warwick, Coventry, CV4 7AL, UK

Kathyrn Dennick, Research Associate, Florence Nightingale School of Nursing and Midwifery, King's College London, SE1 8WA

Kirstie Haywood, Senior Research Fellow, Royal College of Nursing Research Institute, University of Warwick, CV4 7AL, UK

Juul Achten, Research Manager Warwick Orthopaedics, Warwick Medical School, University of Warwick, Coventry, CV4 7AL, UK

Nick Parsons, Senior Research Fellow, Statistics and Epidemiology, Warwick Medical School, University of Warwick, Coventry, CV4 7AL, UK

Xavier Griffin, Specialist Trainee Trauma and Orthopaedic Surgery, Warwick Medical School, University of Warwick, Clinical Sciences Building, Clifford Bridge Road, Coventry, CV2 2DX, UK

Matt Costa, Professor Trauma and Orthopaedics, Warwick Clinical Trials Unit, Warwick Medical School, University of Warwick, Coventry, CV4 7AL, UK

Correspondence to: F.E.Griffiths@warwick.ac.uk

For peer review only

Copyright
The Corresponding Author has the right to grant on behalf of all authors and does grant on behalf of all authors, an exclusive licence (or non exclusive for government employees) on a worldwide basis to the BMJ Publishing Group Ltd to permit this article (if accepted) to be published in BMJ editions and any other BMJPGL products and sublicences such use and exploit all subsidiary rights, as set out in our licence.

Acknowledgements
We thank Katie McGuinness, Kate Dennison, Zoe Buckingham, Catherine Richmond, Rebecca McKeown, Hayley Rice, Mamta Rana, Filo Eales and Gail McCloskey for their assistance in recruitment and all the patients for their time and effort in participating in this study.

Ethical approval
Ethical approval was granted by NHS REC London - Camberwell and St Giles (11/LO/0927) on the 18th August 2011. Further approval was obtained from the research and development department of the University Hospitals Coventry and Warwickshire NHS Trust. This research complies with the Helsinki Declaration.

Access to study data
All authors had full access to all of the data in the study and can take responsibility for the integrity of the data and the accuracy of the data analysis

For peer review only

Abstract

Objective

To explore what patients consider important when evaluating their recovery from hip fracture and to consider how these priorities could be used in the evaluation of the quality of hip fracture services.

Design

Semi-structured interviews exploring the experience of recovery from hip fracture at two time points - four weeks and four months post-operative hip fixation. Two approaches to analysis: thematic analysis of data specifically related to recovery from hip fracture; summarising the participant's experience overall.

Participants

31 participants recruited, of whom 20 were female and 12 were cognitively impaired. Mean age 81.5 years. Interviews provided by 19 patients, 14 carers, and 8 patient/carer dyad; 10 participants were interviewed twice.

Setting

Single major trauma centre in the West Midlands of the UK.

Results

Stable mobility (without falls or fear of falls), for valued activities was considered most important by participants who had some pre-fracture mobility and were able to articulate what they valued during recovery. Mobility was important for managing personal care, for day-to-day activities such as shopping and gardening, and maintenance of mental well-being. Some participants used assistive mobility devices or adapted to their limitations. Others maintained their previous limited function through increased care provision. Many participants were unable to articulate what they valued as hip fracture was perceived as part of their decline with age. The fracture and problems from other health conditions were an inseparable part of one health experience.

~~**Conclusions**~~

~~Patients consistently valued stable~~**Conclusion**

Pre-fracture, mobility, adaptations to reduced mobility before or after fracture, and ~~its role in other basic health domains. For evaluating service quality, no~~whether or not patients' perceive themselves to be declining with age, influence what patients consider important during recovery from hip fracture. No one patient- reported outcome measure (PROM) could

Formatted: Font Alignment: Auto

Formatted: Font color: Black

Formatted: Font color: Black

consistently evaluate recovery quality of care for all patients withfollowing hip fracture. General health-related quality of life tools may provide useful information within clinical trials but may need to be supplemented by specific tools for selected groups, especially those patients with high levels of pre-injury function. .

Formatted: Font color: Black

Key words: Hip fractures, Outcome assessment (Health Care), Interview, Frail older adults

Article summary

Article focus

- The UK NHS has identified the need to evaluate service provision for patients with a hip fracture
- There is increasing expectation that patient-reported outcome measures (PROM) are used within health service evaluation
- We asked the question: what do patients who have recently experienced a hip fracture consider important when evaluating their recovery?

Key messages

- Patients active before their fracture value mobility without falls or fear of falls, to undertake valued activities but many patients consider fracture to be part of their decline with age.
- While noMany patients consider fracture to be part of their decline with age and adapt to reduced mobility or had already adapted pre-fracture
- No one patient reported outcome measure (PROM) could evaluate all aspectsquality of recoverycare for all patients withfollowing hip fracture, general health-related quality of life tools may provide useful information for the majority of patients.

Formatted: Font color: Auto

Strengths and limitations

- The study sample was representative of the age profile, gender balance and dementia levels of NHS patients experiencing hip fractures
- It is possible that those not agreeing to be interviewed were struggling most with recovery.
- The data is limited by the difficulty the more physically and cognitively impaired patients had in giving a detailed account of their health experience.

For peer review only

Introduction

Fragility fracture of the proximal femur (hip fracture) is one of the greatest challenges facing the healthcare community. In 1990, a global incidence of 1.31 million was reported and was associated with 740,000 deaths (1)(1). Hip fractures constitute a heavy socioeconomic burden worldwide. The cost of this clinical problem is estimated at 1.75 million disability adjusted life years lost; 1.4% of the total healthcare burden in established market economies (1)(1). Among those experiencing fragility hip fracture in England, Wales and Northern Ireland, 70% are aged 80 years or older, 73% are female and 34% are cognitively impaired pre-operation. The mortality rate within 30 days of operation was 8.2% in 2013 (2).

The NHS has identified the need to evaluate the quality of service provision for patients with a hip fracture; this evaluation is conducted through the National Hip Fracture Audit Database (NHFD)(2). Currently, aspects of care such as time to surgery, length of patient stay and patient mortality in hospital and 30 day and 120 day follow up are recorded in the NHFD. These data are now used to guide payments to healthcare providers; the payment being increased if the provider supplies 'best practice' care (3)(3). However, while important, there is interest from policy makers in the potential to enhance these currently reported data fields by including and an assessment of outcome as reported by patients. It is increasingly expected that healthcare evaluations should include domains of health that are important to patients (4)(4), captured by well-developed patient-reported outcome measures (PROMs) which aim to assess how patients function and feel in relation to a health condition or associated treatment (5). PROMS capture information that cannot be obtained by other means (5, 6) complementing more traditional performance or process-based measures.

Our aim was to establish whether or not one PROM could be used with all patients who experience a fragility hip fracture as part of the evaluation of the quality of health care for hip fracture delivered by the NHS. For this patient group we were unable to identify a PROM specific to the assessment of hip fracture, and robust evidence of the quality and acceptability of non-hip fracture specific PROMs following completion by patients sustaining a hip fracture is limited (7)(7). Moreover, clarity with regards to the outcomes of healthcare that these patients considers relevant and important does not exist. Appropriate and relevant PROM-based assessment should be underpinned by an understanding of what is important to patients in terms of the outcomes of healthcare. Further, we were concerned to understand

For peer review only

whether, for people with different pre-fracture health and social context, what was important to them during recovery was different. For example, we hypothesised that what is important to a younger, otherwise healthy person experiencing hip fracture may be different from what is important to a person who perceives themselves as nearing the end of life. Good quality care would, as far as possible, enable each patient to achieve what is important to them in terms of recovery. If a PROM is to be used to assess quality of care the measure needs to capture this. We therefore designed an interview study to explore with patients and, where appropriate, their carers, what they consider to be important outcomes and to explore variation across this patient group. Our research questions were:

1. What do patients who have recently experienced a hip fracture consider important when evaluating their recovery?
2. Is there variation between people within this population of the experience of what is considered important in recovery from hip fracture and why?

These research questions are framed by the desire of policy makers to evaluate the quality of care for hip fracture through assessment of recovery from the perspective of the patient.

Method

Study Design

We conducted semi-structured interviews with patients and, where appropriate, their carers at two time points, at approximately four weeks and then again at four months after they had sustained a fragility hip fracture.

Identification of patients with a hip fracture

We recruited participants from an existing cohort study, the Warwick Hip Trauma Evaluation ~~(8)~~(8), that commenced January 2012. This is a cohort of all patients admitted with a hip fracture to a single major trauma centre in the West Midlands of the United Kingdom. As part of their pre-operative assessment, patients were assessed for their capacity to consent using clinical assessment and the Abbreviated Mental Test Score (AMTS) ~~(9)~~(9). The AMTS is a 10-item measure used to rapidly assess the possibility of cognitive impairment in elderly people. A score below 8 suggests cognitive impairment ~~(10)~~(10). Scores less than 8 were taken to indicate that a patient was unlikely to be able to consent for themselves. Those deemed to have capacity for consenting to surgery, based on clinical assessment and AMTS, were considered able to consent for this study. Following the emergency surgery for their

For peer review only

fracture, those with capacity gave written consent to be approached for interview. For those deemed not to have capacity due to cognitive impairment, verbal consent was obtained from their consultee ~~(11).~~(11). Ethical approval was granted by NHS REC London - Camberwell and St Giles (11/LO/0927) on the 18th August 2011.

Sampling

During the data collection period for this study, February to August 2012, we purposefully sampled cohort participants who had reached 4 weeks or 4 months following their hip fracture and had consented to be approached for interview. The time points were chosen to be the same as those used for data collection for the NHFD ~~(12)~~(12). If a PROM were to be used with this patient population to assess quality of care, patients would be asked to complete the PROM at these time points. Our sampling strategy ensured a diverse mix of patients with respect to the following factors: age, gender, AMTS ~~(9)~~(9) and EQ-5D score (13).

Field Code Changed

Interview recruitment and consent process

We contacted eligible patients and carers by telephone just prior to 4 weeks and/or 4 months following hip fracture first to invite them to be interviewed, then to arrange an interview. If patients declined to participate, the reasons offered were recorded. Patients with capacity to consent were contacted directly. For those patients deemed not to have capacity, we contacted their consultee. Patients able to consent for themselves signed their own consent forms. For those unable to consent the consultee signed an agreement form and we aimed to interview a carer as well as the patient (patient/carer dyad). Carers who were interviewed signed a consent form. ~~Recruitment~~Initial analysis commenced during recruitment phase; recruitment continued ~~to~~until data saturation at the first time point. The study flow diagram is at Figure 1.

Interview process

We interviewed participants at their current residence (own home, residential or nursing home) or in hospital. The interviewer was trained in interviewing but did not have clinical knowledge of hip fracture, its treatment or prognosis. Where possible, patients and carers were interviewed alone, however where the carer and patient requested a joint interview (whether or not the patient had cognitive impairment), they were interviewed together. The aim of the interviews was to understand each participant's lived experience of hip fracture ~~(14)~~(14) and the influence of their social context and pre-fracture health. We use the following questions:

For peer review only

- What is a normal day like for you now?
- How bothersome are you finding your hip?
- What is different about your life now compared to just before your injury?
- Compared to just before your injury what has stayed the same?
- Which of these make the most difference to your life?

The interviewer encouraged participants to talk about the experience in whatever order they chose and using terms meaningful to them. Later in the interview we prompted, where necessary, for clarification about what in the patient experience was related to the hip fracture. Towards the end of the interview we directly asked what was important to them in terms of recovery if this had not already been talked about by the participant, using the following questions:

- What is important to you in terms of your recovery?
- Where would you like to see yourself in the future in relation to your recovery (i.e. the next few weeks and months)?
- If a friend or neighbour were asking you now about how well you are recovering – what has been important to you that you would tell them about?
- If a doctor or nurse was asking you now about how well you are recovering – what would be important for the doctor or nurse to ask about?

Consideration was given to the potential challenges associated with interviewing older adults, for example by giving potential participants sufficient time to decide whether or not to participate and minimising burden and fatigue through streamlining questions ~~(15).~~(15). The interview process, questions and prompts were refined by the study team during the initial stage of data collection~~.~~, particularly adding questions and prompts to focus the participant on recovery from their hip fracture. Questions were similar for both patient and carer. Interviews were audio~~-~~-recorded~~ and transcribed verbatim~~. For one interview, audio recording was not feasible due to the noisy environment so extensive field notes were taken~~ and transcribed.~~. For all interviews the researcher made reflective field notes to assist interpretation of the interview data.

Analysis

~~Interview~~Interviews and field notes were transcribed and transcripts~~ were~~ checked, anonymised and uploaded into Nvivo software ~~(16)~~(16). Initial analysis involved data immersion, reading and re-reading each transcript and discussion of the interview transcripts by the research team. Our research team was multi-disciplinary: social science, behavioural science, health science, orthopaedic surgery and statistics. All team members read at least five transcripts so all transcripts were read by at least two team members. From the data we

For peer review only

identified and crystallised what was important for participants that was specific to hip fracture recovery ~~(17)~~(17). We found that the interviews at four weeks and four months covered very similar issues, although, as would be expected, what the participants reported about each issue four weeks and at four months was different, as recovery was more advanced at four months. As our analysis aimed to identify what patients consider important when evaluating their recovery rather than the detail of recovery itself, we treated all the interviews related to one participant as one set of data. During data interpretation we took account of the timing of the interview, whether the interview data was from a patient or carer or patient/carer dyad, and field notes. ~~Two different approaches to analysis were then undertaken in response to our research questions~~(17). For data collection and analysis we took a phenomenological approach in that we sought to understand participant's experience of hip fracture recovery and the influence of their context on this (14, 18) and concurrently we took a selective realist position (19) in that we recognised hip fracture as an event identifiable by means other than through the participant's account.

~~To answer our first research question, we~~We used two different approaches to analysis to answer our research questions. For the first research question, which is concerned with the whole groups of participants, we used thematic analysis (20). We searched the transcripts for any mention by the participants of what was important to them during recovery from hip fracture. These were discussed at team analysis meetings. Transcripts were then coded in NVivo. As coding proceeded, we reviewed these codes at our team analysis meetings and combined them into themes. After we had read, discussed and then coded ten transcripts we found no additional themes in the remaining data. Double coding was undertaken for one in four transcripts and coding compared and discussed to check consistency of final coding. During analysis we became aware that although the data from different participants could be coded under the same theme such as mobility, ~~there was variation in~~ the experience of recovery was very different for different people. This led us to our second research question and analysis approach.

To answer our second research question~~, from close reading of the first five interview transcripts we~~ we used cross case analysis (21). We considered each participant as an individual 'case' living within their particular context (22, 23) and through comparison of cases sought to understand how they varied. To develop our matrix for the cross case analysis (21), we closely read five participant data sets then developed, from the data, a template for

For peer review only

summarising the experience of hip fracture recovery for each patient carer dyad. This involved considering each set of interviews as a whole, reading and rereading the text and writing a summary of the patient/carer journey and all that influenced it. We reviewed the summaries at our data analysis meetings and from these initial summaries we developed a draft template. We refined the template based on the data as we summarised and discussed further transcripts. The template included: current and recent past living arrangements and environment, day-to-day life now and in the recent past, the impact of the hip fracture and its management, what was changing in day-to-day life as they recovered, the extent to which the patient referred specifically to the fracture and their ability to engage in the interview. Each of these formed a data row in our matrix with a column for each participant. The data fromabout each patient or patient/carer dyad was summarised into the template with a second research team member reviewing each summary against the data. To qualitatively understand the variation in the experience of what was considered important for recovery, we compared these summaries.

Results

Twenty one patients were interviewed on one occasion and 10 were interviewed twice giving a total of 31 patient participants and 41 interviews. Of the 31 patient participants, 20 (64.5%) were female, the mean age was 81.5 years (SD 9.2, range 61-96) and 12 (39%) scored less than eight on the AMTS. Of the 41 interviews, 24 were conducted three to nine weeks, and 17 were conducted 14 to 23 weeks after the hip fracture. Nineteen interviews were with the patient only, 14 with carer only, and eight with patient/carer dyads. Interviews lasted between 20 and 90 minutes. Despite framing the interview for interviewees as exploring the experience of hip fracture, many interviewees talked about general health issues. Although we prompted to clarify what was related to their fracture, in many interviews it was difficult to disentangle the impact of the fracture from the impact of other health problems. Some interviews contained almost no data that was clearly related to the fracture. From the perspective of the patient, all their health problems were part of one experience. The absence of data clearly related to the fracture was more marked in the four month compared to four week interviews. We therefore decided not to attempt interviews at 12 months post fracture as originally planned (8)We therefore decided not to attempt interviews at 12 months post fracture as originally planned (8). The following sections report our analysis. Illustrative quotations from data are labelled with the age and gender of the patient, time since hip fracture and whether the quotation was from the patient or carer.

For peer review only

What is important to patients when evaluating their recovery?

From our systematic search of the interviews for data related to recovery from the hip fracture we identified the following themes: mobility, valued day-to-day activities, self-care, pain, mental wellbeing, fear of falling and leg shortening. When talking about mobility, day-to-day activities or self-care participants also talked about their level of independence.

Mobility

This was the most prominent theme, although when talking about mobility the interviewees often mentioned other themes. Mobile participants reported limited mobility in the weeks post operation and valued any improvement.

> I'm walking with a walking stick at the moment. I've been down the park and back…I can usually get around [the house] without the walking stick, and I can get up and down stairs no problem. I get upstairs with my good leg and downstairs with my bad leg. (Participant 6, male, age 78, 5 weeks post operation)

By four months, for many participants mobility had improved, and they were happy that they were returning to normal mobility.

> I can't rush round like I did, but eventually that will come…I mean it's pretty normal now, but I think it's going to be a while before I can actually walk as I did and I probably won't walk as I did… when I came home [from hospital] I was still hobbling... but now I'm more or less…walking normal, especially with the stick (Participant 10, female, age 83, 18 weeks post operation)

For those with limited mobility before hip fracture any unaided improvement was limited to the pre-fracture level but also valued.

> The operation was successful and got him back to normal right from the start, right from the very first day that he had it done. He was able to then walk pain free with a Zimmer frame to the toilet. The staff were all saying it was amazing how well he was walking and he would soon be back to normal, but what they didn't realise was that he was walking normally. (Carer of participant 1, male, age 84, 16 weeks post operation)

Other participants were using mobility aids that they had not been using regularly before the fracture. For some, the addition of mobility aids enabled greater security of mobility than prior to their fracture.

> Her mobility's getting better. I think she'll cope with the frame. She's had a couple of falls in the home, earlier when she was forgetting that she had to use the frame. She'd get out of bed and not use the frame and consequently fall. But she's got in the habit

For peer review only

> of using it now… she's not falling, which is a bonus. (Carer of participant 13, female, age 87, 14 weeks post operation)

Valued day-to-day activities

Those who were active prior to their fracture talked about the frustration of the restriction in their activities particularly in the weeks following the fracture.

> I'm back on what I call domestic duties – washing up! But the thing that is frustrating is that I can't get outside and do any gardening. (Participant 12, male, age 78, 6 weeks post operation)

> I just miss getting up and getting out. I never stayed in. I'd go out in the morning and come back and then I'd go out again, I just used to go out looking round the shops. I just get these crossword books and I do those. (Participant 20, female, age 92, 5 weeks post operation)

Participants who were active before their fracture were usually able to resume valued activities but had some limitations which remained a frustration.

> I can do little (gardening) jobs but because I haven't got as much movement in the hip joints, I find it difficult to go down on my hands and knees…If I go down on one knee it's difficult to get up again so that's not possible but I can do things that are higher up, I can trim. (Participant 15, female, age 61, 15 weeks post operation)

> I'm tackling a little bit of cooking now. I started to cook myself some nice lunches and I haven't got round to the… scones … I made one lot when I came home and I thought, I can't be bothered anymore. (Participant 10, female, age 83, 18 weeks post operation)

Some participants returned to valued activities through adapting how they did them, this participant using a wheelchair for the first time.

> Over the last three weeks, when we go out shopping now, I can't go down the aisles, so [daughter] gets me a (wheel)chair and I can sit in the chair and then say what shopping I need, that is very good. (Participant 9, female, age 92, 18 weeks post operation)

Participants who no longer undertook valued activities that involved significant mobility were content to continue as they were, for example, occupying themselves with visits from family and reading.

Personal care

Washing, dressing and getting to the toilet was talked about in interviews, but in many cases it was not clear whether difficulties with personal care were specifically due to the fracture. A

For peer review only

few interviewees talked about problems with incontinence but again it was unclear whether this was specific to the fracture. Most patients had a commode or had arranged to sleep near the bathroom in the weeks immediately after the fracture. Some participants were able to describe problems with self-care specific to the hip fracture.

> I'm …not able to put a sock or anything on my injured leg. I can manage now with my trouser leg and throw these jogging trousers and hook my leg into them but I have to ask my husband if I need to put a sock or a shoe, or my slipper on that foot. (Participant 15, female, age 61, 6 weeks post operation)

At the second interview this participant was pleased to report that she now needed very little help with self-care, at least in part through wearing alternative footwear.

> I still have to throw my clothes and hook them onto the foot to get dressed. I couldn't wear lace-up shoes or anything like that because I couldn't tie them up, but things like slip-ons and sandals I can get on quite easily, so I'm fairly independent – I am independent really, I just need help with cutting my toenails and that – those on the right foot that's all. (Participant 15, female, age 61, 15 weeks post operation)

Pain

Although pain was talked about by some interviewees it was not considered a major problem.

> So here I am, four or five weeks [post operation], I get a little bit of pain, not a lot. (Participant 7, female, age 70, 5 weeks post operation)

> The pain was so bad before I had it done, and I just couldn't believe the relief after the operation when I was walking in the hospital and I had one of those pushers you know. And there was no pain. And I kept thinking, I can't believe this, and that's how it's been. I've never had any pain, not at all.
> (Participant 10, female, age 83, 18 weeks post operation)

> There's several times, like when I have got to get up those steps. I put my right foot first and bring my left foot up, and once or twice… you step on your left, and it's still there, lets you know it's still tender. (Participant 12, male, age 78, 16 weeks post operation)

Mental wellbeing

Low mood or depression associated with the reduced mobility due to the fracture was reported by a few interviewees, emphasising the great value placed by interviewees on being independently mobile.

> He can't walk and that, to him he'd rather die. I'll be honest with you he's said it once or twice, "Let me go". And I said, "No you're not going no-where". And then the other day for the first time, but he hasn't said it since, "I'm going to commit suicide", I said, "No you're not, you're not". (Carer of participant 31, male, age 84, 5 week post operation)

For peer review only

> For me it was a massive problem and caused me depression. To me is the most important thing, the mental aspect of taking away somebody's freedom to be able to move around and go to the shops and do all that sort of thing.
> (Participant 7, female, age 70, 23 weeks post operation)

Fear of falling

The experience of the fracture left a few participants with a fear of falling and sustaining a further fracture.

> I think it frightened him more than anything else. He's frightened he'll fall over again and do it again, that bothers him more than anything else. Because now when he stands up at all to try and walk he's frightened he's going to fall over and the same thing will happen all over again. (Carer of participant 11, male, age 84, 7 weeks post operation)

> I've got to watch what I'm doing. If I catch my foot on [paving stone], I can go over again. (Participant 12, male, age 78, 16 weeks post operation)

The fear of falling was sometimes expressed by a family member. When talking about his frustration at not being able to work in the garden, participant 6 added

> All the rain has made it very slippery, and [wife] says, "No way do you go out there."
> (Participant 12, male, age 78, 6 weeks post operation)

This emphasises the value given to mobility without falls or fear of falls by interviewees.

Leg shortening

This is a problem that is common following extra-capsular fracture of the proximal femur. One interviewee described her concerns about this.

> One leg is now shorter than the other so that makes walking a bit difficult because it gives me back pain. (Participant 15, female, age 61, 15 weeks post operation)

Is there variation within this population of the experience of what is considered important in recovery from hip fracture?

Our sample included patients from across a spectrum that extended from those who were physically and mentally active prior to their fracture through to those who, pre-fracture, had been immobile due to conditions such as multiple sclerosis, chronic obstructive airways disease and arthritis, and those with severe cognitive impairment. Although when talking about what was important to them when evaluating their recovery from hip fracture, patients from across this spectrum talked about similar themes, their experiences of what was important ~~varied.~~ was different for different people. In Box 1 we present condensed versions of the interview summaries developed during our second analysis approach, for participants

chosen to represent the whole spectrum of patients. We indicate whether the data was provided by patient, carer or both.

Recovery as a return to pre-fracture state or as part of aging and decline

Every patient interviewed had experienced a hip fracture and surgery, so in physical terms all of them had, for a period of time, been somewhat impaired compared to their pre-fracture state. Four weeks post-operation, those who were active pre-fracture talked in terms of regaining a recovered state that was similar to their pre-fracture state although with some minor adaptations (participants 15 and 20 in box 1). Whilst these participants expressed worry about how well they might function in the future, there was, nevertheless, determination to progress to as full a recovery as possible. Four months post-operation many of these participants had all but regained their pre-fracture level of activity. Among participants with severely limited mobility pre-fracture, some were able to identify specific activities which were more difficult post-fracture than pre-fracture, such as putting on socks and getting in and out of bed. Some were also able to identify specific improvements in mobility post operation (see participants 9 and 15 in box 1). These participants described a process of recovery although it was very limited.

In contrast, for other participants, the fracture was just one part of a process of aging and decline. For example, participant 11 (see box 1) had been very limited in his activities before the fracture. Post fracture he needed adaptations to his home and increased care support post fracture to enable him to continue to manage at home. The mobility of participant 18 had declined and she had started using a wheelchair instead of her mobility scooter to get out of the house. However, it was unclear whether the decline was due to the concurrent heart failure or the fracture. Those who were the most physically or cognitively impaired pre-fracture did not talk about regaining a recovered state but about a state of no change. They continued with their limited activities as before (for example: participants 23 and 26 in box 1). For one participant, the only change was her move to a new nursing home (participant 5 in box 1). Participants with cognitive impairment were often unaware of having experienced a fracture (Participant 1 box 1).

Recovery through adaptation

In the face of their physical limitations, most participants made adaptations that mitigated the effect of the fracture; for example employing a cleaner, moving to a nursing home or using a

walking aid or other assistive device. For those who were active pre-fracture, adaptation was mostly considered temporary, although at 4 months there was some evidence that active patients had adapted to some limitations such as being unable to kneel for gardening or limiting time spent shopping to avoid exhaustion. For some participants who had been experiencing decline in their mobility pre-fracture, the fracture precipitated adaptations that they had not previously considered but made their life easier. These included using a wheelchair for shopping, having a new ramp built for getting in and out of the house in a wheelchair, using a walking aid or employing professional carers to assist with personal care. For some, their own or their carer's fear of further falls limited their mobility or at least limited how far they tested their ability to walk. Poor weather conditions exacerbated this fear, but adaptations to the environment such as walking aids or handrails lessened the fear.

Discussion

Following hip fracture, for those who had some pre-fracture mobility and able to articulate what they value during recovery, stable mobility, that is, mobility without the experience of or fear of falling, and mobility that that allows people to undertake valued activities are most valued. The ability to walk is important but so too are other leg movements needed for activities such as gardening or using transport. For some participants, maintaining mobility, however limited, was achieved by using assistive devices or working out new ways of doing an activity. Some participants adapted to their limitations, for example wearing different footwear or adjusting their expectations of what they could achieve. Others maintained their previous limited function through increased care provision.

Patients also consistently valued certain basic domains of health, such as pain (or lack of it), day-to-day activities, personal care and mental well-being. However, many participants in this study were unable to articulate what was important to them in terms of recovery from hip fracture. The hip fracture was just one part of their decline with age and its impact could not be disentangled from the impact of other health issues. The level of recovery perceived by a participant was influenced by their pre-fracture state and their ability to make adaptions during recovery.

Strengths and weaknesses of the study

When the mortality rate post operation is taken into account, including the higher mortality amongst older females, the study sample was broadly representative of the age profile and

For peer review only

gender balance of the population of England, Wales and Northern Ireland experiencing hip fractures (2). We used a higher cut off for assessment of cognitive impairment (score of 8 on AMTS) compared to the NHFD (score of 6 on AMTS). This is likely to explain our higher proportion of participants with cognitive impairment compared to the average in the NHFD.

More research time was spent on recruitment than any other aspect of the study as it proved difficult. When contacted about the interview study, potential participants talked about other priorities or concerns that prevented them agreeing to interview, or they simply did not wish to be interviewed. It is possible that those not interviewed were struggling most with recovery. Our data is also limited by the difficulty some frail older adults have in giving a detailed account of their health experience (18).Our data is also limited by the difficulty some frail older adults have in giving a detailed account of their health experience (24). Interview data is jointly constructed by interviewer and interviewee (19) and our interviewer had no clinical knowledge of hip fractures.(25) and our interviewer had no clinical knowledge of hip fractures. This reduced the likelihood of the interviewer influencing the data. A clinician undertaking the interviews would have the knowledge to help the patient tease out whether health problems were fracture related or not. However, this would have obscured the important finding, that participants often experienced their fracture as part of, rather than separate to, their other existing health problems. We relied on carer's accounts for some participants. We found they talked about the same themes as the participants. However, for those with cognitive impairment, some carers were unable to provide detailed data as they had limited day-to-day contact with the participant. We did not attempt to check with participants about our interpretation of the data to avoid further burden for them.

Comparison with other studies

There are similarities between our findings and other qualitative studies of similar populations. A Swedish team that explored engagement with rehabilitation post hip fracture found a similar spectrum of participants (20).(26). They classified their participants as: those who were frail and in need of support but did not request it; those who were dependent and took no active part in rehabilitation and those who were self-sufficient. Another Swedish study, undertaken with people 12 months after their hip fracture found that mobility and a return to normal activities were key outcomes for patients (21).(27). An Australian study of mobility post-fracture found that reduced level of mobility was associated with fear of falling, physical limitations from other illness and social/environmental factors (22).(28). Our

results also echo findings from across the research literature on the experience of health and illness. For example, the difficulty disentangling the impact of one health condition from other co-morbidities has been found for mental health conditions (23).(29). The acceptance of an acute health problem as being part of the aging process has been found for conditions such as stroke (24)(30). Recalibration to altered circumstances in response to a sudden injury has also been described (25)(31), as have the adaptations- both physical and psychological- that people make in order to maintain their quality of life (26).(32). Reduced expectations of health and acceptance of limited function have been described among elderly women (27).(33). Fear of falling is common among older people generally (28).(34). The consistency between our findings and other studies suggests that there is now sufficient qualitative evidence to inform policy decisions about the choice of appropriate PROMS for assessing recovery from hip fracture.

Implications for clinicians and policymakers

This study was undertaken in response to a potential policy change involving the use of a PROM to assess patient recovery from hip fracture, the results of which would form part of the evaluation of the quality of care provided for hip fracture. ForWe conclude that for the population experiencing fragility hip fractures, it is unlikely that a single PROM specific to hip fracture could be developed which is relevant to the whole spectrum of patients. An assessment that focuses on mobility of the hip would be relevant for many patients, and mobility impacts on other health domains. However, with any form of assessment of mobility, pre-fracture status would have to be taken into account. Some patients had limited pre-fracture mobility at the hip so a lack of mobility during recovery may not reflect the quality of care. In addition there are other factors that influence the perception of recovery by patients. These include adaptations that they or their carers make to compensate for their reduced mobility, and patient perception of whether or not they are at the stage in life where decline is inevitable. Quality of care is only one of a number of interrelated factors that influence the patient's perception of recovery from hip fracture.

Several of the themes described by interviewees - mobility, day-to-day activities, self-care, pain and mental wellbeing, are similar to the domains included in currently available generic measures including the EuroQoL EQ-5D (13)(13), the Short Form 36-item Health Survey (SF-36) (29)(35) and the WHOQoL-BREF (30).(36). Both the EQ-5D (3L) and the SF-36 (version 1) have been widely used in trials of people sustaining hip fractures, but for both

For peer review only

measures evidence of essential measurement and practical properties is limited (7)(7). In the context of a clinical trial where patients are randomised to an intervention and control arm, these generic measures may be appropriate but they may need to be supplemented by specific tools for selected groups, such as patients with high-levels of pre-injury function.

In the context of assessing quality of care for a patient population as diverse as those experiencing hip fracture, it may be impossible to devise a single PROM that will be appropriate for all patients. Although quality of care may be one factor that will influence recovery as perceived by a patient, their pre-fracture state, adaptations that they or their carers make to their reduced mobility, and their perception of whether or not they are at the stage in life where decline is inevitable will all influence how they answer questions contained within a PROM.

For peer review only

61 year old female social worker who lives with her husband. Before her fracture she was working full time and, for recreation, taking country walks, undertaking all types of gardening activities and playing with her grandchildren. Post fracture fixation (6 weeks) she described using crutches to get around the garden and shops, needing help with putting on socks and cutting toe nails, and was unable to climb stairs. She talked in terms of improvement and expectation of returning to work and full activity including cleaning and gardening. By the second interview she was frustrated that recovery was so slow but she could identify the ways in which she had continued to recover. (Participant 15, interviewed 6 weeks and 15 weeks post operation)

92 year old female who lives alone in her own flat within a sheltered housing complex. Prior to the hip fracture she looked after herself and did her own washing, but had a cleaner to undertake heavy household chores. She spent most of each day out and about at the shops, engaging in social activities, bingo and on outings. She had no other illnesses. Post-fracture fixation she talked about having some initial pain and problems lifting her leg after the operation but was now mobile about her home with a walking frame. The housing complex has a lift which she now used. She was intending to return to getting out and about as she was before her fracture. (Participant 20, interviewed 5 weeks post operation)

92 year old female lives alone with husband. Daughter visits several times a week to help. Poor hearing. Difficult to disentangle what was before and after fracture. Seems to have been able to walk around house, undertake self-care and microwave own meals pre-fracture. Post fixation of the hip fracture, patient slowly improved walking. Life seems very similar to before fracture except need for walking aid, inability to put on socks and husband now microwaves the meals. (Participant 9, interviewed 9 weeks post operation)

70 year old male retired painter and decorator who lives with his wife and enjoys almost daily visits from his grandchildren. Mobility restricted to 5-6 metres for more than two years prior to fracture due to knee pain and chronic obstructive pulmonary disease. When interviewed he describes struggling to get up the stairs, get in and out of bed, put his shoes and socks on, and bend down. Although his mobility was severely restricted prior to his fracture, he described being unable get around as much as he had done before the fracture. He noted some improvement over recent weeks, as he no longer needed two sticks for walking, only one.
(Participant 3, interviewed 15 weeks post operation)

84 year old male with dementia, who has some lucid moments and some recall of falling and hurting himself. He lives with his wife who looks after him and they have a cleaner to do heavy housework. Wife provided interview, involving the patient in the latter half when he woke up. Patient's walking was gradually slowing and he had a number of falls before his fracture. Fracture occurred while walking in shopping area with his wife. Since fixation of the fracture patient has required assistance with personal care, has professional carers four times a day, and the bathroom has been adapted for his limited mobility. The interviewee had difficulty distinguishing decline due to old age and change due to the fracture. The patient complained of some pain but it was unclear whether this was from the fracture or previously established osteoarthritis. Before the fracture both patient and wife had ceased all non-essential activities except for a weekly trip to the shops so daily life had changed little except for more care provision. (Participant 11, interviewed 7 weeks post operation)

For peer review only

74 year old female who lives with husband. Patient lived with severe rheumatoid arthritis for 30 years. Developed heart failure and admitted to hospital with shortness of breath and confusion. Fell while in hospital and fractured her hip. Mobility before hip fracture very limited – able to walk slowly in house and garden, undertake light chores, and use scooter to go shopping. Became worse with breathing difficulty. Mobility remained reduced after hospital admission. Able to take step slowly in house with support. Uses wheelchair to go out of house – a new ramp improved this by second interview. Unclear how much mobility change was due to the fracture and how much due to heart failure. (Participant 18, interviewed 6 weeks and 18 weeks post operation)

88 year old female retired teacher, who lives with her son and has a diagnosis of multiple sclerosis. The patient wove together pre and post injury experience in her account, making it difficult to disentangle. She said her son does the cooking and cleaning and her daughter assists with self-care. She has a close family, feels well supported and has lots of visitors – friends, grandchildren and great grandchildren. Her main interest beyond seeing friends and family is reading. She described being content with life. Prior to her fracture she was unwell with an infection and recounts using a frame for mobility which she still uses. (Participant 23, interviewed 5 weeks post operation)

85 year old female living in a nursing home. Her daughter visits alternate days. Her daughter provided the interview data. The patient has dementia but otherwise had been well before the fracture. Patient gets up and walks about herself, and takes herself to the toilet. She enjoys sitting and chatting. The patient does not remember the injury. Her life has not changed from how it was pre injury. The daughter did not mention any fracture-specific issues related to recovery. (Participant 26, interviewed 6 weeks post operation)

84 year old female with limited English language. Pre-injury she had carers to assist her with all her personal needs. The injury had occurred whilst being hoisted. Post injury her main concern was that at discharge from hospital, after a three month stay, she was sent to a nursing home where she knew no-one. The patient repeatedly expressed distress about being in the nursing home but did not talk about the fracture.
(Participant 5, interviewed 18 weeks post operation)

84 year old male who has dementia. He lives alone but received visits three times a day from his son who provides meals. Son was interviewed. Arthritis of knee limited mobility before the fracture. Spent most of the day sitting. At weekends prior to fracture patient went to neighbour's house for evening meal. Patient fell and sustained fracture while walking to neighbour's house. Patient does not recall fracture. At time of interview, the patient was as mobile as pre operation limited by pain and stiffness from arthritis. Not yet visiting neighbour but this was because family was discouraging this in case he falls again rather than due to mobility. (Participant 1, interviewed 16 weeks post operation)

Box 1 Summaries of the data about individual patients and their recovery from a hip fracture

For peer review only

Competing interest statement
All authors have completed the Unified Competing Interest form at www.icmje.org/coi_disclosure.pdf (available on request from the corresponding author) and declare: funding from the National Institute of Health Research, University of Warwick, and University Hospitals Coventry and Warwickshire NHS trust.

Authorship
MC, FG, JA, XG, KH and FB contributed to the conception and design of the study. FB, VM and KD conducted the interviews. All authors contributed to analysis and interpretation of data. FG, KD and VM drafted the article and all authors revised it critically for important intellectual content. All authors gave final approval of the version to be published.

Funding
This study was funded by a Programme Development Grant from the National Institute of Health Research (RP-DG-1210-10022). This trial was co-sponsored by the University of Warwick and University Hospitals Coventry and Warwickshire NHS trust. This manuscript presents independent research. The views expressed are those of the authors.

Data sharing: no additional data available

References

1. Johnell O, Kanis JA. An estimate of the worldwide prevalence, mortality and disability associated with hip fracture. . Osteoporosis international: a journal established as result of cooperation between the European Foundation for Osteoporosis and the National Osteoporosis Foundation of the USA. 2004;15:897-902.
2. Johansen A, Wakeman R, Boulton C, Plant F, Roberts J, Williams A. National Hip Fracture Database National report 2013. Clinical Effectiveness and Evaluation Unit at the Royal College of Physicians, 2013.
3. Department of Health. Update to Best Practice Tariffs: Department of Health; 2011 [5th November 2012]. Available from: http://www.dh.gov.uk/health/2011/12/bpt-update/.
4. Devlin N, Appleby J. Getting the Most Out of PROMs. Putting health outcomes at the heart of NHS decision-making. London: The King's Fund, 2010.
5. Patrick DL, Burke LB, Powers JH, Scott JA, Rock EP, Dawisha S, et al. Patient-reported outcomes to support medical product labeling claims: FDA perspective. Value Health. 2007;10:S125-S37.
6. Fitzpatrick R. Patient-reported outcome measures and performance measurement. In: Smith P, Mossialos E, Papnicolas I, Leatherman S, editors. Performance measurement for health system improvement: experiences, challenges and prospects. Cambridge: Cambridge University Press; 2009.
7. Haywood K, Brett J, Tutton E, Staniszewska S, editors. How well are we measuring patient-centred outcomes following traumatic hip fracture? A systematic review of patient-reported outcome measures (PROMs). Royal College of Nursing International Research Conference; 2012 23-25th April 2012; London: Royal College of Nursing.
8. Griffin X, Achten J, Parsons N, Boardman F, Griffiths F, Costa M. The Warwick Hip Trauma Evaluation - an abridged protocol for the WHiTE Study. A multiple embedded randomised controlled trial cohort study. . Bone Joint Res. 2012;1(11):310-4.
9. Hodkinson HM. Evaluation of a mental test score for the assessment of mental impairment in the elderly. Age and Ageing. 1972;1(4):233-8.

For peer review only

10. MacKenzie DM, Copp P, Shaw RJ, Goodwin GM. Brief cognitive screening of the elderly: a comparison of the Mini-Mental State Examination (MMSE), Abbreviated Mental Test (AMT) and Mental Status Questionnaire (MSQ). Psychol Med. 1996;26(2):427-30.
11. Mental Capacity Act 2005, (2005).
12. Currie C, Fleming S, Partridge M, Plant F, Wakeman R, Williams A. National Hip Fracture Database National Report. 2010.
13. The EuroQoL Group. EuroQoL: a new facility for the measurement of health-related quality of life. Health Policy. 1990;16(3):199-208.
14. McConnell-Henry T, Chapman Y, Francis K. Husserl and Heidegger: Exploring the disparity. International Journal of Nursing Practice. 2009;15(1):7-15.
15. Jacelon C. Older adults' participation in research. Nurse Researcher. 2007;14(4):64-73.
16. NVivo qualitative data analysis software. 10 ed. Melbourne, Australia: QSR International Pty Ltd; 2012.
17. Borkan J. In: Crabtree BF, Miller WL, editors. Doing Qualitative Research. Second ed. London: Sage Publications; 1999.
18. Leder D. A tale of two bodies: the cartesian corpse and the lived body. The body in medical thought and practice. Dordrecht: Klewer Academic Publishers; 1992. p. 17-36.
19. Miller A. Realism. In: Zalta EN, editor. The Stanford Encyclopedia of Philosophy: Stanford University; 2012.
20. Green J, Thorogood N. Qualitative Methods for Health Research. London: Sage; 2004.
21. Miles MB, Huberman AM. Qualitative data analysis. Thousand Oaks, CA: Sage; 1994.
22. Ragin CC. 'Casing' and the process of social inquiry. In: Becker CCRaH, editor. What is a case? Exploring the foundations of social inquiry. Cambridge: Cambridge University Press; 1992.
23. Griffiths F, Borkan J, Byrne D, Crabtree B, Dowrick C, Gunn J, et al. Developing evidence for how to tailor medical interventions for the individual patient. Qualitative Health Research. 2010;20(12):1629-41.
24. Kvigne K, Gjengedal E, Kirkevold M. Gaining access to the life-world of women suffering from stroke: methodological issues in empirical phenomenological studies. Journal of Advanced Nursing. 2002;40(1):61-8.
~~19~~25. Mishler E. Research interviewing: Context and narrative. Cambridge, MA: Harvard University Press; 1986.
~~20~~26. Olsson LE, Nystrom AE, Karlsson J, Ekman I. Admitted with a hip fracture: patient perceptions of rehabilitation. J Clin Nurs. 2007;16(5):853-9.
~~21~~27. Ziden L, Scherman MH, Wenestam CG. The break remains - elderly people's experiences of a hip fracture 1 year after discharge. Disabil Rehabil. 2010;32(2):103-13.
~~22~~28. Taylor NF, Barelli C, Harding KE. Community ambulation before and after hip fracture: a qualitative analysis. Disabil Rehabil. 2010;32(15):1281-90.
~~23~~29. Connell J, Brazier J, O'Cathain A, Lloyd-Jones M, Paisley S. Quality of life of people with mental health problems: a synthesis of qualitative research. Health and Quality of Life Outcomes. 2012;10:138.
~~24~~30. Faircloth CA, Boylstein C, Rittman M, Young ME, Gubrium J. Sudden illness and biographical flow in narratives of stroke recovery. Sociology of Health and Illness. 2004;26(2):242-61.
~~25~~31. Bury M. Chronic illness as biographical disruption. Sociology of Health and Illness. 1982;4(2):169-82.
~~26~~32. Clarke P, Black S. Quality of Life Following Stroke: Negotiating Disability, Identity, and Resources. Journal of Applied Gerontology. 2005;24:319-36.
~~27~~33. Chambers P. Getting Beyond 'Mustn't Grumble' and 'What Can I Expect At My Age': A Narrative Approach to Older Women's Health Stories. In: Narrative, Memory and Health. Huddersfield: University of Huddersfield; 2003. p. 127-39.

For peer review only

~~28~~34. Scheffer AC, Schuurmans MJ, van Dijk N, van der Hooft T, de Rooij SE. Fear of falling: measurement strategy, prevalence, risk factors and consequences among older persons. Age and Ageing. 2008;37(1):19-24.

~~29~~35. Ware Jr. J, Gandek B. Overview of the SF-36 Health Survey and the International Quality of Life Assessment (IQOLA) Project. Journal of clinical epidemiology. 1998;51(11):1203-14.

~~30~~36. Molzahn A, Kalfoss M, Schick Makaroff K, Skevington S. Comparing the importance of different aspects of quality of life to older adults across diverse cultures. Age Ageing. 2011;40(2):192-9.

For peer review only

Evaluating recovery following hip fracture: a qualitative interview study of what is important to patients

No	Item	Guide questions/description	Included in manuscript?
**Domain 1: Research team and reflexivity**			
Personal Characteristics			
1.	Interviewer/facilitator	Which author/s conducted the interview or focus group?	yes
2.	Credentials	What were the researcher's credentials? *E.g. PhD, MD*	No place to provide this in submission process
3.	Occupation	What was their occupation at the time of the study?	yes
4.	Gender	Was the researcher male or female?	Names are ones that are usually gender specific
5.	Experience and training	What experience or training did the researcher have?	Job title provided
Relationship with participants			
6.	Relationship established	Was a relationship established prior to study commencement?	Yes
7.	Participant knowledge of the interviewer	What did the participants know about the researcher? e.*g. personal goals, reasons for doing the research*	The participant information sheet followed standard UK ethics guidelines and was approved by an ethics committee (details in submission form)
8.	Interviewer characteristics	What characteristics were reported about the interviewer/facilitator? e.g. *Bias, assumptions, reasons*	Job titles provided

For peer review only

No	Item	Guide questions/description	Included in manuscript?
		and interests in the research topic	
**Domain 2: study design**			
Theoretical framework			
9.	Methodological orientation and Theory	What methodological orientation was stated to underpin the study? *e.g. grounded theory, discourse analysis, ethnography, phenomenology, content analysis*	The description of our analysis process makes it clear that we were using a modified grounded theory approach. We describe the approach in detail rather than giving a label which could mislead.
Participant selection			
10.	Sampling	How were participants selected? *e.g. purposive, convenience, consecutive, snowball*	Yes
11.	Method of approach	How were participants approached? e.*g. face-to-face, telephone, mail, email*	Yes
12.	Sample size	How many participants were in the study?	Yes
13.	Non-participation	How many people refused to participate or dropped out? Reasons?	Yes
Setting			
14.	Setting of data collection	Where was the data collected? e.*g. home, clinic, workplace*	Yes
15.	Presence of non-participants	Was anyone else present besides the participants and researchers?	Yes
16.	Description of sample	What are the important	Yes

For peer review only

No	Item	Guide questions/description	Included in manuscript?
		characteristics of the sample? *e.g. demographic data, date*	
Data collection			
17.	Interview guide	Were questions, prompts, guides provided by the authors? Was it pilot tested?	Yes
18.	Repeat interviews	Were repeat interviews carried out? If yes, how many?	Yes
19.	Audio/visual recording	Did the research use audio or visual recording to collect the data?	Yes
20.	Field notes	Were field notes made during and/or after the interview or focus group?	Yes
21.	Duration	What was the duration of the interviews or focus group?	Yes
22.	Data saturation	Was data saturation discussed?	Yes
23.	Transcripts returned	Were transcripts returned to participants for comment and/or correction?	Not mentioned as not done with this population
**Domain 3: analysis and findings**z			
Data analysis			
24.	Number of data coders	How many data coders coded the data?	yes
25.	Description of the coding tree	Did authors provide a description of the coding tree?	First analysis approach involved thematic coding. No sub codes were used.
26.	Derivation of themes	Were themes identified in advance or derived from the data?	Yes
27.	Software	What software, if applicable, was used to manage the data?	Yes
28.	Participant checking	Did participants provide feedback on the findings?	Not mentioned as not done with this population
Reporting			
29.	Quotations presented	Were participant quotations	Yes

For peer review only

No	Item	Guide questions/description	Included in manuscript?
		presented to illustrate the themes / findings? Was each quotation identified? e*.g. participant number*	
30.	Data and findings consistent	Was there consistency between the data presented and the findings?	Yes
31.	Clarity of major themes	Were major themes clearly presented in the findings?	Yes
32.	Clarity of minor themes	Is there a description of diverse cases or discussion of minor themes?	Yes

For peer review only
